# On the use of simulation in robotics: Opportunities, challenges, and suggestions for moving forward

**DOI:** 10.1073/pnas.1907856118

**Published:** 2020-12-28

**Authors:** HeeSun Choi, Cindy Crump, Christian Duriez, Asher Elmquist, Gregory Hager, David Han, Frank Hearl, Jessica Hodgins, Abhinandan Jain, Frederick Leve, Chen Li, Franziska Meier, Dan Negrut, Ludovic Righetti, Alberto Rodriguez, Jie Tan, Jeff Trinkle

**Affiliations:** ^a^Department of Psychological Sciences, Texas Tech University, Lubbock, TX 79409;; ^b^Medical Simulation and Information Sciences Research Program, US Army Medical Research and Materiel Command, Fort Detrick, MD 21702;; ^c^INRIA (Institut National de Recherche en Informatique et en Automatique)–Lille–Nord-Europe, 59650 Villeneuve d’Ascq, France;; ^d^Department of Mechanical Engineering, University of Wisconsin-Madison, Madison, WI 53706;; ^e^Department of Computer Science, Johns Hopkins University, Baltimore, MD 21218-2686;; ^f^US Army Research Laboratory, Adelphi, MD 20783-1138;; ^g^Office of the Director, National Institute for Occupational Safety and Health, Washington, DC 20201;; ^h^Robotics Institute, Computer Science Department, Carnegie Mellon University, Pittsburgh, PA 15213-3890;; ^i^Mobility and Robotic Systems Section, Jet Propulsion Laboratory, California Institute of Technology, Pasadena, CA 91109;; ^j^Dynamical Systems and Control Theory Division, Air Force Office of Scientific Research, Arlington, VA 22203;; ^k^Department of Mechanical Engineering, Johns Hopkins University, Baltimore, MD 21218-2682;; ^l^Autonomous Motion Division, Max Planck Institute for Intelligent Systems, 72076 Tübingen, Germany;; ^m^Electrical & Computer Engineering Department, New York University, Brooklyn, NY 11201;; ^n^Mechanical & Aerospace Engineering Department, New York University, Brooklyn, NY 11201;; ^o^Department of Mechanical Engineering, Massachusetts Institute of Technology, Cambridge, MA 02139;; ^p^Robot Locomotion Team, Google AI, Mountain View, CA 94043;; ^q^Department of Computer Science and Engineering, Lehigh University, Bethlehem, PA 18015

**Keywords:** robotics, simulation, design, controls, machine learning

## Abstract

The last five years marked a surge in interest for and use of smart robots, which operate in dynamic and unstructured environments and might interact with humans. We posit that well-validated computer simulation can provide a virtual proving ground that in many cases is instrumental in understanding safely, faster, at lower costs, and more thoroughly how the robots of the future should be designed and controlled for safe operation and improved performance. Against this backdrop, we discuss how simulation can help in robotics, barriers that currently prevent its broad adoption, and potential steps that can eliminate some of these barriers. The points and recommendations made concern the following simulation-in-robotics aspects: simulation of the dynamics of the robot; simulation of the virtual world; simulation of the sensing of this virtual world; simulation of the interaction between the human and the robot; and, in less depth, simulation of the communication between robots. This Perspectives contribution summarizes the points of view that coalesced during a 2018 National Science Foundation/Department of Defense/National Institute for Standards and Technology workshop dedicated to the topic at hand. The meeting brought together participants from a range of organizations, disciplines, and application fields, with expertise at the intersection of robotics, machine learning, and physics-based simulation.

Robots are no longer stiff/rigid implements operating in the structured environment of assembly lines and performing scripted and limited sets of operations. Emerging artificial intelligence techniques will endow the next generation of robots with mobility and decision-making skills. These robots will be flexible and reconfigurable; interact with humans; and operate in environments that are unstructured, uncertain, and rapidly changing in time. It is expected of them to assume new roles such as operating on highways as autonomous vehicles, in nursing homes assisting social workers, in schools tutoring young learners, underwater managing oil spills, and in the adverse and cluttered environments of search-and-rescue missions or remotely performing surgeries. While physically testing these robots before deployment is mandatory, through simulation, the engineering design process can be accelerated, made more cost effective, and benefit from more thorough testing.

This Perspective is a reflection of ideas, observations, and suggestions that emerged during a 1-d workshop on the topic of using simulation in robotics (1). This participant input, in raw form and organized in anonymized decks of slides, is available online (2). The hope is that this Perspective, which provides a synthesis of the input contributed by the workshop participants, becomes a useful reference, resource, and call to arms for colleagues from the simulation community interested in applying their expertise in the rapidly evolving field of smart robotics. To that end, this contribution seeks to identify opportunities, point out challenges, and suggest possible next steps vis-à-vis the goal of increasing the role that computer simulation plays in smart robotics.

This Perspective is organized in three parts. The first part summarizes benefits that simulation could deliver upon its use in designing robots over the next decade, i.e., near term. A subsequent section points out hurdles that hinder the wide use of simulation in practice. Finally, we identify steps believed to accelerate the near-term role that simulation plays in robotics. The intent was to generate a snapshot that captures this group’s perspective on the potential, hurdles, and path forward for the simulation-in-robotics idea. This contribution is not a review of the simulation-in-robotics literature; references were used sparingly to point the reader to contributions that further elaborate on ideas that were only briefly stated in this Perspective and, where necessary, to provide a historical perspective on the topic at hand. The purposely short reference list was dictated by publication length constraints and the stated purpose of this Perspective—to provide a “perspectives” opinion rather than a review of the state of the art.

## How Simulation Is or Could Be Useful in Robotics

Ideas embedded in preworkshop contributions (2) and further discussions during the joint meeting brought into focus several simulation-in-robotics opportunities: 1) Generate, expeditiously and at low cost, large amounts of training data for machine learning; 2) accelerate the engineering design cycle, and reduce its costs; 3) provide an accelerated, safe, and fully controlled virtual testing and verification environment; 4) facilitate the development of more intelligent robots; and 5) facilitate the understanding of human–robot interactions (HRI).

### Opportunity 1: Generate, Expeditiously and at Low Cost, Large Amounts of Training Data for Machine Learning.

The recent surge of machine-learning use in defining control policies (3), and the associated need for a wealth of training data, provided a major impetus to the use of simulation in robotics. A validated simulation platform becomes an ideal proving ground for developing systems that can both learn from their mistakes and be verifiable. Simulation can be used to optimize new behaviors and to carry out tasks never known to be possible. The advantage of using simulation to generate training data becomes even more compelling when the software used can draw on ubiquitous high-throughput computing resources, i.e., using multiple nodes to carry out batches of simulations in parallel and perhaps in the cloud. Using simulation to generate training data for control policies is not a silver bullet owing to the so-called simulation-to-reality gap (4), which is touched upon later in this article. Enhancing the transferability attribute of simulation-learned control policies represents an area of active research (5–7).

### Opportunity 2: Accelerate the Engineering Design Cycle, and Reduce Its Costs.

There are two time-consuming stages in the design of a new robot: the mechanical design and the control policy design. The former is concerned with producing a solution that can execute a predefined set of tasks. The latter is concerned with endowing the robot with smarts to actually carry out the tasks that it has the potential to execute. For both mechanical and control policy design, one typically produces prototypes that iteratively improve on previous versions until a certain prototype is acceptable; this prototype becomes a candidate solution. The iterative process to produce the candidate solution is time consuming. Additionally, it can be expensive, unsafe (for humans or the robot hardware), and sometimes impractical (if designing a rover for Mars, testing cannot be done in Martian conditions). Moving from a model-free to a model-based design approach, i.e., carrying out the iterative loop in simulation (8), can reduce the time associated with the design process. Indeed, changing a rover suspension design to assess trafficability in simulation can be as fast as modifying a handful of parameters in a template file that defines the geometry of the vehicle (9). By comparison, physically modifying the suspension of a prototype is significantly more time consuming. Additional time and cost savings are incurred in the subsequent stage, the testing of the candidate design. Before it becomes the solution, the candidate design is subjected to extensive additional testing in which a limited collection of candidate design clones is assessed via a predefined evaluation process. Physically building the collection of candidate designs is time consuming and costly since there is no assembly line ready yet and each clone is handcrafted. In simulation, testing the candidate might be as simple as copying the model files to different folders and conducting the predefined evaluation process using high-throughput, parallel computing.

### Opportunity 3: Provide an Accelerated, Safe, and Fully Controlled Virtual Testing and Verification Environment.

Approaches to verification of autonomous systems are in their infancy (10). Approaches to verification and “debugging” of autonomous robotic systems that learn online are essentially nonexistent. Repeatability, particularly with respect to stress/corner cases; full control of the experiment insofar as the “environment” is concerned; and the lack of risk to human and hardware damage are three attributes that can make simulation instrumental in establishing principled protocols for autonomous system verification and, by extension, industry standards and guidelines. Against this backdrop, as autonomous systems cannot foresee unknowns that they may encounter, novel formal verification schemes can be developed to verify specifications such as safety in real time under assumed uncertainty in the system and its environment (11).

Simulation can play an important role in providing insights into multirobot, collaborative scenarios (12, 13). Collaborative, multirobot systems can exclusively comprise robots interacting with each other based on their own local decision-making algorithms that factor in sensed and/or shared information or can include human interaction as in, for instance, search and rescue scenarios. As the number of robot–robot and/or human–robot interactions increases, so does the complexity of designing and verifying these systems. Physical testing and verification are daunting as the collection of scenarios to probe increases quadratically with the number of agents in the system. Moreover, it is difficult to systematically test in real conditions multirobot systems used, for instance, in environmental monitoring, off-road mobility/survivability, surveillance, or infrastructure management, etc., due in part to the stiff challenges posed by operating groups of agents in such environments. Simulation is very convenient in such scenarios, given that it can also be used to probe for interagent connectivity failure or hostile network penetration. Hostile/adversarial attacks aim at more than multirobot network penetration—the robot’s control stack and sensing system are two other targets (14). One of the most compelling cases for use of simulation in robotics can be made in conjunction with the need to thoroughly check the consequence of such attacks. Do the checks designed to guard against adversarial attacks work as expected? What is a safe way to counteract an adversarial attack? The answer to these and similar questions is critical for safety and regulatory purposes yet direct hardware testing is costly and potentially unsafe (bulky robots gone awry can become outright dangerous). Simulating the response of the robotic system in cases of failure, while potentially performing a task, permits the investigation of fail–safe strategies for the system as well as the design of security, antibreach policies. Without simulation, one is limited in what and how many scenarios can be tested.

### Opportunity 4: Facilitate the Development of More Intelligent Robots.

In this argument, the intelligence metric is tied to the breadth of scenarios in which the robot is expected to successfully fulfill a set mission. By this metric, industrial robots are at one end of the spectrum. They operate in very structured and static environments (known ahead of time) and are trained to perform a narrow and a priori established catalog of operations. The opportunity to develop more intelligent robots is a consequence of several technological advances: Machine-learning techniques opened the door to new control policies (3); the compute speeds available on an affordable power budget have increased substantially; sensing is sharper and more affordable; actuation is gradually becoming more sophisticated; and low latency communication/networking and edge computing allow for real-time, just-in-time off-loading of heavy-duty computation during operation. Against this backdrop, simulation can be a catalyst for the next stage in the evolution of smart robots, one in which they operate in unstructured environments and engage in decision-making activities. Just as the concepts of morality (set rules), prior experience, and ability to predict the consequence of one’s actions shape the decision-making process in humans, a set of rules (including ethical) and an ability to predict consequences/outcomes through online simulation can shape the decision-making process of robots through introspection (15). A rule that states “plates shall not drop on the floor” combined with the ability to run thousands of parallel simulation scenarios faster than real time should determine the robot to place on a table the plate at the top of the stack that it carries rather than one from somewhere in the middle of the stack. The ability to foresee online the outcome of a possible action through the lens of simulation is anchored by spectacular gains in computation speed. For instance, recent graphics processing unit (GPU) cards can manage the simultaneous execution of more than 150,000 computing threads (16). With simple enough simulation kernels, this translates into peeking into the future via as many possible scenarios, leading to introspective control policies that complement the established model predictive control approach and more recent techniques that rely on on/offline machine-learning solutions (17).

### Opportunity 5: Facilitate the Understanding of HRIs.

The ability to simulate the interaction between the robot and the human opens the door to experimentation with tele-surgical robotics in semiautonomous or autonomous operation, reducing risks to individuals in dangerous work environments or working in cooperation with collaborative and mobile robots in a shared workplace, eliminating or reducing repetitive motion trauma and musculoskeletal overload, and reducing fatalities and injuries from motor vehicle incidents as autonomous vehicles are yet another form in which robots interact with humans. The human–robot interplay can project specialized medical expertise and care to any point of need, at any time. This is of particular interest for teams operating remotely, e.g., small teams of first responders, teams of astronauts, etc. Finally, one important facet of the science of HRI pertains to the aspect of establishing trust between human and robot in shared decision making (18). Development of simulation tools that better represent the psycho-social nature of HRI and enable a common operating “picture” of possible solution sets for decision making may foster trust and establish a baseline for more effective collaboration (19).

## Barriers to the Use of Simulation in Robotics

This section summarizes issues that, to various degrees, limit the role played by simulation in robotics. A noncomprehensive list of open problems vis-á-vis the topic at hand includes the following: 1) In a scarcity of solutions, producing a simulation platform for robotics requires broad multidisciplinary expertise and sustained software development commitment; 2) existing modeling languages are immature at a time when a robotics simulation ontology is only slowly emerging; 3) model composability needs further improvements; 4) simulation is not fast enough; 5) uncertainty is marginally handled; 6) model calibration can be tedious; 7) data-driven approaches (surrogate models; replacing simulation with an oracle) are only slowly emerging; 8) difficulties in gauging the needed level of model complexity; and 9) handling the human–robot interaction.

### Issue 1: In a Scarcity of Solutions, Producing a Simulation Platform for Robotics Requires Broad Multidisciplinary Expertise and Sustained Software Development Commitment.

After an evolution that spanned decades, computer-aided engineering (CAE), i.e., the use of computer simulation in customary engineering, has become a linchpin in the design process. For instance, one can carry out a highly predictive finite-element analysis of an engine crank shaft or of an airplane wing to accurately identify vibration/flutter modes. Likewise, a vehicle ride analysis can be performed via a dynamics simulation in which a vehicle operating on a flat, rigid surface follows a sequence of driving maneuvers to collect information for a subsequent noise/vibration/harshness analysis.

Compared to customary CAE, simulation in robotics is less structured and vastly more multidisciplinary. The “less structured” attribute is tied to the observation that a relatively simple set of boundary conditions and load functions is enough to define the backdrop for a CAE-type analysis. In robotics, the backdrop is more complex and uncertain. For instance, the robot can operate in and interact with an unstructured and evolving world; or a human can be part of the equation. The “multidisciplinary” attribute is a consequence of the fact that for simulation in robotics, in addition to producing models for robot dynamics, which is in the purview of CAE, one has to engage in non-CAE tasks; e.g., simulate perception; represent in high fidelity the unstructured environment surrounding the robot (this virtual world might have to be itself simulated since its state can be influenced by the robot dynamics, e.g., rover changing the world by creating deep ruts that must be captured by the camera sensor of a following rover); simulate the human dimension in HRI; and simulate agent-to-agent or agent-to-infrastructure communication. These simulator subcomponents, which are tied to the dynamics, sensing, virtual world, human participation, and communication facets, render the simulation-in-robotics task highly multidisciplinary. As an example, consider perception simulation—it alone calls for knowledge in several disciplines as robotics applications rely on multiple classes of sensors, e.g., Global Positioning System (GPS), inertial measurement unit (IMU), Light Detection and Ranging (LiDAR), camera, radar, thermal, etc., each drawing on vastly different physics for characterizing the sensing paradigms that come into play. Finally, one has to integrate these subcomponents and turn the sum of the parts into a useable simulation platform, a process that adds a software engineering facet to the multidisciplinary nature of the task at hand.

### Issue 2: Existing Modeling Languages Are Immature at a Time When a Robotics Simulation Ontology Is Only Slowly Emerging.

Specifying/defining/setting up a scenario model for simulation in robotics can be daunting. Indeed, one has to specify a slew of parameters that define the geometry of the robot, its kinematics, loads, actuators, sensor package information, the virtual world it operates in (weather, time of day, satellite position, terrain properties, etc.), communication types and protocols, control policy type, etc. The scenario modeling abstractions that are needed should be conducive to yielding models with hierarchical structures that accommodate the user’s requirements vis-à-vis the level of fidelity rendered in simulation—from less accurate but expeditious to highly detailed/accurate but computationally more demanding. Gazebo (20, 21) has made good strides in this direction, continuing in the tradition of previous tools like USARSim (22). However, the ontology associated with Gazebo’s modeling approach and other similar efforts (23) is limited in scope. Dwelling on the Gazebo example, the platform exposes one simulation interface to several engines: ODE (24), Bullet (25), DART (26), and Simbody (27). Presently, the Gazebo simulation-in-robotics ontology does not include concepts such as fluid viscosity, material dissipation model, soil plasticity, Drucker–Prager yield, etc., since none of the Gazebo-exposed physics engines supports fluid dynamics, plasticity in soils, finite-element analysis, etc. There are simulation engines that have started to support multiphysics with various degrees of success (28, 29) yet these engines presently come short on exposing robotics-focused modeling abstractions. In moving forward, lessons in scenario modeling can be learned from software platforms used to create computer games (28, 30, 31). These platforms are used with good success by several robotics simulation engines (22, 32, 33) to create complex virtual worlds with visually appealing graphics. However, the gaming platforms are not designed to accurately simulate the physics governing the behavior of robots and their interactions with the environment. Consequently, their predictive attributes are limited owing to an emphasis on plausibility rather than accuracy.

### Issue 3: Model Composability Needs Further Improvements.

A rich universe opens up when multiple agents participate in the same scenario (34). Composability refers to the ability to combine, LEGO style, basic constructs/submodels into complex simulation scenarios. In this approach, complex scenarios are investigated via composed models that draw on validated, discipline-oriented, submodels. This calls for more than just the ability to build on a hierarchical representation of a model. Indeed, there is an interplay at runtime between these subcomponents that takes place in the presence of dynamic (evolving) environments and human participation. For instance, the submodels communicate with each other (agent to agent, agent to infrastructure, etc.) and interact with each other through contact, friction, visual, or auditory cues, etc. These aspects need to be supported in a physics-based fashion by the simulation engine, and this is what makes this composability problem more than a “model representation,” i.e., a preprocessing, problem. One additional layer of complexity comes into play if a real-time constraint must be met for scenarios that include numerous robots. Composability with scalability typically calls for simulators that rely on parallel computing, which further complicates the problem at hand due to time and space coherence requirements—robots should propagate their dynamics in the same world time and be able to sense each other although they might run on different hardware assets linked through a fast interconnect fabric. There are several sources of inspiration for handling composability, e.g., V-REP (23), which embraces composability yet has other limitations in terms of breadth of physics simulated. Similarly, there are computer-aided design (CAD)-to-dynamics modeling pipelines (which leverage, for instance, SolidWorks or MATLAB) and video gaming-based solutions that can lighten the burden of reaching composability with scalability. Indeed, the game community has established sophisticated game authoring tools that implicitly handle model composability. On the down side, they are massive chunks of code; cumbersome to work with; and, as pointed out in issue 2, only partially cover the robotics community’s needs owing to emphasizing credibility in appearance rather than accuracy in results.

### Issue 4: Simulation Is Not Fast Enough.

Robotics simulation is customarily used for mechanical design or control purposes. In controls, the simulations should run fast, in fact, faster than real time for model predictive control or online policy inference (while not ideal, slower than real-time performance is acceptable for offline policy inference oftentimes employed in machine learning). Presently, by and large, robot models that use only rigid bodies run in real time as long as the number of bodies is in the neighborhood of 10 to 15 and the number of collisions with friction and contact is low (25, 35, 36). However, the real-time constraint is daunting for models that contain flexible/soft/deformable components and/or numerous contacts with friction. An additional and sizable simulation slowdown is associated with scenarios that include unstructured and changing environments, e.g., deformable terrain, cluttered environments, fluid–solid interaction, etc. Bringing this type of physics into the model is important when robots venture outside controlled environments and operate in complex real-world scenarios. Depending on the goal pursued, i.e., mechanical design vs. controls, managing the simulation time calls for compromises, e.g., reduced number of iterations in a numerical algorithm, a coarser mesh for collision detection, use of rigid instead of deformable terrain, reliance on more rudimentary sensing, use of rigid instead of compliant elements, two-dimensional instead of three-dimensionbal dynamics, model reduction, etc. Note that the nature of this compromise also depends on the computing power available, which is markedly different for offline and online simulation. Indeed, a complex model can run real time in a mechanical design analysis, when one relies on power-hungry hardware such as GPUs, only to run too slowly in an online setup where weight and/or power constraints limit the compute hardware options.

### Issue 5: Uncertainty Is Marginally Handled.

Friction, impact, contact, actuator noise, wear and tear, uncertain external loads, complex and unstructured environments, etc., are a few of the many sources of uncertainty that mar the operation of a robot. These sources of uncertainty must also be present in simulation. The uncertainties encountered are both aleatory (due, for instance, to the complexity of the dynamic response in the presence of friction and contact phenomena) (37) and epistemic (e.g., unknown simulation parameters for a selected model for friction, e.g., Coulomb friction) (38). Accounting for uncertainty must be done during both model generation and simulation phases. In model setup, one has to provide mechanisms to inject uncertainty in the model: e.g., allowing the friction coefficient to change in a deformable and heterogeneous terrain, the geometry of various components to be less than perfect, the actuation forces/torques to display delays, etc. In running simulations, one has to robustly handle lack of smoothness associated with the solution of the robot dynamics: e.g., stick–slip conditions, impact, control policies that are nonsmooth, event-triggered changes that occur sporadically to suddenly change loads or boundary conditions, etc. A perspective that might be useful is a statistical one, in which confidence bounds gauge the extent to which one can rely on simulation results (39). This uncertainty quantification mindset is pivotal, since robots will oftentimes have limited knowledge about the environment they operate in and stimuli they are subject to.

### Issue 6: Model Calibration Can Be Tedious.

Populating a model with correct parameters is time consuming and most often an ad hoc, one-off, process. Pragmatically speaking, the goal is to do predictive analysis, not to find parameters for a model. This observation explains why this topic receives less attention than the task of generating the model, which nonetheless is populated with these very parameters that ultimately dictate its quality. When experimental data for the robot are available, classic identification methods are effective provided the parameter space is not large (40). Unfortunately, at design phase, robots exist only as CAD drafts yet to be instantiated in reality; as such, experimental data are unavailable. Even when the robots exist, experimental data for calibration might be hard to procure. For instance, friction and contact simulation for rigid-wheel/deformable-terrain interaction requires more than 10 parameters, which are typically determined through a bevameter test carried out in the field (41). Although the rover might exist, experimental data for Martian soil is very limited and a bevameter test is out of the question. Recent efforts aimed at simplifying calibration rely on machine learning and other statistical approaches to automate a labor- and time-intensive process (6, 42, 43). For large parameter sets, brute force approaches can leverage powerful processors to perform statistically guided extensive searches for parameter values (44).

### Issue 7: Data-Driven Approaches (Surrogate Models; Replacing Simulation with an Oracle) Are Only Slowly Emerging.

The standardization of robotic platforms opens the possibility to collect large amounts of data (sometimes provided by the manufacturer) in real-world scenarios that could be leveraged by machine-learning techniques to build predictive models directly from data (8). The hope is that using statistical-learning techniques, one could bypass simulation-specific hurdles such as model generation and calibration by constructing “oracles” that are able to predict the next system state given its current one. The outcome of a data-driven approach might not be a complete elimination of the simulation. The approach could be used to methodically reduce model (and therefore computational) complexity through systematic dimensional reduction and/or model compression (45, 46). However, the data-driven approaches in robotics simulation are in their infancy and principled methodologies to design and validate data-driven models remain to be identified. In addressing the issue at hand, one is faced with the practical challenge of developing realistic use cases and generating enough data to represent “truth” or “reality.” This is not a trivial task. For instance, what types of data, problems, and level of authenticity are required to better capture and model the socio-physical characteristics of human–robot interaction scenarios? Note that in producing these surrogate models, one can substitute experimental data with simulator-generated data. Indeed, if a simulator and a model exist, the data produced by the simulator–model combination, just like experimental data, can be used to produce an “oracle” that within a controlled set of regimes can yield results close to those produced in simulation. In other words, the simulator provides the data that lead to its obsolescence.

### Issue 8: Difficulties in Gauging the Needed Level of Model Complexity.

Complex models set up to produce highly accurate results most often lead to long run times and require a wealth of model parameters. It is not uncommon that lack of good parameters in these models leads to worse results that take longer to produce than what can be obtained using simpler models. Likewise, debugging complex models is time consuming since it is difficult to discern whether subpar results are due to poor model parameters or to weaknesses in the modeling and numerical solution techniques anchoring the simulator. Against this backdrop, there is a lack of consensus regarding when complex models are needed, i.e., at what point a model confines our ability to discover through simulation. For instance, in machine learning, sophisticated models are expected to better negotiate the simulation-to-reality gap, i.e., to yield simulation-learned control policies that work well on the real robot (4). Recent results show, though, that this gap can be successfully negotiated learning with simple models. In a “domain randomization” approach, simple and therefore expeditious models are repeatedly used to canvas broad model parameter spaces via expeditious simulation to improve control policy robustness (5). Unfortunately, this example cannot be generalized and one should be cognizant of the fact that validated simulation tools serve multiple end goals—human-in-the-loop simulation, the designing of better control policies, improving mechanical performance, auditing for safety purposes, etc. This suggests that diversity in the level of model complexity is inevitable and the practitioner has to rely on past experience, understanding of the real-world problem, and knowledge of the simulation tool to make the right call. Choosing a simple model for a complex problem might produce misleading results; conversely, choosing too complex of a model for a simple problem might take an unreasonable amount of effort to set up and lead to prohibitively long simulation times. These are two outcomes that each can discredit the idea of using simulation in robotics; they can also be used to make a case that simulation in robotics is part science and part practice.

### Issue 9: Handling the Human–Robot Interaction.

One conspicuous challenge in HRI simulation is tied to the speed of execution: The robot simulation should be fast enough to support real-time interaction between the human and the robot model (human-in-the-loop simulation). This poses several challenges; see issue 4. Speed of execution ceases to be a matter of concern if there is no actual human in the loop; i.e., the human component is simulated as well. Indeed, in robot training, the human is replaced with an avatar endowed with the same traits that the human would display in interaction with the robot. Creating avatars is as difficult as humans are diverse, each person a unique and complex web of intertwined physical, social, emotional, cognitive, and psychological threads. Numerous empirical models have been created to abstract human behavior in robotics and virtual reality (47). Ideally, the wisdom and insights that these studies have yielded would be aggregated in priors for mathematical models. This would mark a departure from today’s empiricism in human modeling in robotics and represent a step toward the adoption of statistical models of the human in HRI tasks. While progress has been reported in capturing mechanistic traits in HRI, e.g., models for the mechanics of the human body insofar as simulating surgery procedures is concerned (48, 49), numerous questions remain unanswered in relation to abstracting in mathematical models the psychological underpinnings that trigger in humans states of anxiety, fear, comfort, stress, etc. In this context, the ability to control and display emotions in avatars represents a prerequisite for endowing smart robots with a sense of empathy in their interaction with humans (50).

## Suggested Steps for Making Simulation More Useful in Robotics

The next two subsections summarize, respectively, recommendations and high-vantage-point observations that emerged at the conclusion of the simulation-in-robotics workshop.

### “Nuts-and-Bolts” Recommendations.

The recommendations below are made for a generic agency that considers making investments in the simulation-in-robotics area while the agency is at the stage of assembling a call for proposals. The recommendations could also be informative to someone who contemplates becoming involved in the research and development effort associated with the simulation-in-robotics topic. The list is not comprehensive; it compiles only several initiatives deemed as of higher priority: 1) Foster the development and validation of open-source simulation platforms; 2) foster efforts that seek to establish open and community-curated libraries of validated models; 3) organize several simulation-in-robotics grand challenge competitions; 4) encourage efforts that seek to characterize the human–robot interaction; 5) encourage simulation solutions for soft robotics; and 6) emphasize use of emerging hardware architectures.

#### Recommendation 1: Foster the development and validation of open-source simulation platforms.

A robust and feature-rich set of four or five simulation tools available in the open-source domain is critical to advancing the state of the art in robotics. Validated open-source platforms democratize the simulation-in-robotics effort and inspire/inform future, more refined open-source or commercial efforts. Trusted open-source solutions are quickly embraced; support the idea of reproducibility/verifiability in science; and have the side effect of immediately raising the bar for the commercial tools, which must necessarily up the ante. Owing to the breadth of robotics applications, it is likely that no single platform will emerge as the solution of choice for all targeted simulation scenarios, which provides the rationale for the “four or five simulation platforms” recommendation above.

#### Recommendation 2: Foster efforts that seek to establish open and community-curated libraries of validated models.

Customarily, simulation in robotics calls for the interplay of three types of submodels: robots, synthetic worlds, and sensors. In some cases, the human component comes into play, and, for multirobot scenarios, one might need to simulate the communication layer. Any one of these submodels is complex, both to generate and to endow with meaningful parameters; see issue 6. With an eye toward scenario composability, it would be useful and impactful to establish a set of freely available, ready-to-go scenario models that build off existing robot/virtual world/sensor/human/communication submodels. Establishing model libraries is an arduous and long process that has one prerequisite: The community must coalesce around a modeling approach and ontology. The Unified Robot Description Format (URDF) (51) represents an early step in this direction, yet its modeling schema remains to be endowed with additional modeling elements. The existence of a rich and broadly adopted modeling language and model library will have the side benefit of providing an incentive for the manufacturers to augment these libraries with validated models associated with their robotics solutions. Finally, it is also imperative to identify new ways in which models can be validated. Fresh perspectives are needed in this regard, particularly when the robots simulated have not yet been realized (52).

#### Recommendation 3: Organize several simulation-in-robotics grand challenge competitions.

Over the next decade, the community would benefit from a sequence of four or five grand challenge competitions. Run as a sequence, perhaps 2 y apart and akin to the 2013 Defense Advanced Research Projects Agency’s (DARPA) Virtual Robotics Challenge (21), these competitions could pay dividends in several ways. First, carefully chosen challenge themes would focus the community effort. Without direction and clear purpose, the community can choose to focus limited resources along technical thrusts of secondary relevance. Second, a set of well-chosen challenge themes would be a catalyst for cross-discipline collaboration. The current robotics simulation gaps are best addressed by multidisciplinary teams, combining model developers and model users, particularly when the latter have expertise in physical testing. Third, such an effort will foster an ecosystem buildup effort to produce the methods, tools, models, and validation metrics of the trade. Fourth, this effort will likely produce a catalog of reliable simulation platforms available, as well as a community-sanctioned set of recommendations that indicates which tool works well in what scenarios. Finally, it is highly likely that these competitions will attract a lot of public interest, which bodes well for promoting the field and getting the attention of tomorrow’s roboticists.

#### Recommendation 4: Encourage efforts that seek to characterize the human–robot interaction.

The issue of establishing human models that capture mechanical attributes of the body and/or psychological and cognitive traits of human behavior is cross-cutting. Applications in which HRI will come into play include robotic surgery, which is relevant in surgery training and remotely treating patients in remote/disaster zones; assisting seniors with tasks such as dressing, personal hygiene, cleaning, and cooking; and assisting individuals with limited ambulatory ability with transportation needs, etc. The body of work in the area of HRI modeling is meager, which explains the limited knowledge vis-à-vis the issue of human cognitive performance in HRI. In this context, there is a very limited set of science-based requirements and thresholds for safe human–robot interaction.

#### Recommendation 5: Encourage simulation solutions for soft robotics.

To date, robotics simulation has almost exclusively drawn on rigid body dynamics. Indeed, the underlying modeling is simpler, the software implementation effort is more reasonable, and the simulation runtimes are shorter. Looking ahead, support for soft robotics is critical in several fields, e.g., HRI and biomimetic robots. It is anticipated that embracing compliance in the robotics models will elicit new approaches to handling frictional contact with the potential benefit of alleviating numerical artifacts/paradoxes brought to the fore by the rigid body model (53). Generating through simulation sensory-motor data that match the multiresolution dynamics, noise, softness, etc., of sensors and actuators during complex tasks that include both compliance and frictional contacts is poised to open the door to a systematic study of the sensory-motor space for robotic manipulation and locomotion.

#### Recommendation 6: Emphasize use of emerging hardware architectures.

Recent advances in high-bandwidth memory and large core/processor counts can partially alleviate increased computational loads associated with terramechanics, soft robotics, fluid–solid interaction, and real-time simulation. Looking beyond GPU and multicore computing, a federated approach (54) in which various components of a model are handled separately by different solvers in a cosimulation framework would facilitate a plug-and-play vision that enables scalability, open the door to contributions coming from multiple groups, and enable the adoption of best-in-class solvers for subproblems.

### “High-Vantage-Point” Aspects.

Unlike the previous subsection, which provided pointed recommendations, the focus below is on capstone observations: 1) Building a simulation-in-robotics ecosystem requires a sustained and multidisciplinary effort that poses numerous open questions in basic research; 2) simulation in robotics has a foundational and sizable software development component to it; and 3) simulation in robotics provides a grand challenge that has manifest societal impact.

#### High level 1: Building a simulation-in-robotics ecosystem requires a sustained and multidisciplinary effort that poses numerous open questions in basic research.

Several types of simulation techniques have to be combined to produce a simulation-in-robotics solution platform. A dynamics engine is needed to capture the time evolution of the robots in environments that can be unstructured and themselves be changing in time; sensor simulation is required in controlling the agents; meaningful sensor simulation is predicated on the ability to simulate the environment and model its unstructured and time-dependent nature, simulating the human dimension in HRI; and finally, communication simulation comes into play for agent-to-agent or agent-to-infrastructure information exchange. Addressing these open problems requires basic research in a variety of areas: physics-based modeling (friction, contact, impact, soft/compliant bodies, fluid–solid interaction, terramechanics), numerical methods (fast real-time solvers, linear solvers, optimization techniques, computational geometry, the numerical solution of differential algebraic and partial differential equations, nonlinear finite-element analysis, computational fluid dynamics, etc.), software development (hardware-aware software), and data analysis (uncertainty quantification). Serendipitously, several funded initiatives are ongoing, e.g., soft robotics (NSF) and terramechanics (Department of Defense), and bound to have an impact for the problem at hand. Nonetheless, the majority of the simulation-in-robotics multidisciplinary open issues, e.g., modeling the human component in HRI, sensor modeling, model composability, etc., linger and at best are tangentially addressed in a context that lacks synergy and is nonprogrammatic. Against this backdrop, we are of the opinion that the benefits of a broad multiagency initiative could be twofold: Stimulate fundamental research in relevant areas and foster its rapid translation into open-source simulation platforms. A broad initiative of this caliber would bring together, ideally in an international framework, research groups from academia and agile software development groups from academia, research laboratories, and industry.

#### High level 2: Simulation in robotics has a foundational and sizable software development component to it.

While posing significant challenges, software development is not a research activity insofar as simulation in robotics is concerned. Yet the process adopted for developing the necessary software infrastructure along with the terms under which the software is released play a role in how soon simulation makes a difference in robotics. An important aspect is whether or not the software that underlies a simulation-in-robotics initiative should be released as open source. Likewise, there are several licenses, some more permissive than others, vis-à-vis how open source can be used/modified/distributed. The salient point is that software that solves the problem at hand is desirable in any form. Perhaps, at the onset of a simulation-in-robotics initiative open source released under a permissive license such as MIT is more attractive only for the reason that a component of it, be it a graphical user interface, data input/output facilities, a particular algorithm, a collision detection implementation, etc., might be recycled by another effort that itself might be open or closed source. In our experience, the argument that an open-source code grows faster owing to contributions from volunteers has proved largely unsubstantial. Indeed, the level of skill and expertise required to make meaningful contributions to a project is prohibitive, particularly early on in the trail-blazing phase of the project. In fact, the point in time in the software life cycle when volunteers can help marks the moment when the project would ideally go commercial or be sponsored by such an entity. This brings up the question of who should develop the software that enables the simulation-in-robotics vision. Encouraging domain experts to engage in software development pursuits that have a manifest translation attribute, i.e., demonstrating new algorithms, modeling approaches, etc., would be beneficial, particularly at the onset of the initiative and when done with a mindset of generating open-source software. Ideally, these domain-expert generated software components would be recycled by more mature simulation platforms. In this ecosystem, as the field matures, one can hope that bigger and perhaps commercial entities would step in to carry the burden of adding the features of convenience that accelerate solution adoption.

#### High level 3: Simulation in robotics provides a grand challenge that has manifest societal impact.

It is this group’s belief that computer simulation can and should play an important role in smart robotics. We take our cue from the enthusiastic adoption and broad impact that computer simulation has had in other industries and endeavors—from building cars and airplanes to planetary exploration. Yet compared to CAD, computer-aided manufacturing (CAM), and CAE solutions that anchor both the product life cycle in industry and a vigorous research enterprise in academia, the simulation in the robotics field is in its infancy. Moreover, beyond lack of maturity, simulation in robotics is faced with the task of serving user groups pulling in different directions by virtue of them being engaged in different activities such as design (Will it break? Is it fast enough? Can we build it?), controls (How can we make it reconfigure? How does it climb stairs? How does it work with other robots? Will it work under limited sensing?), machine learning (learn gradually without forgetting; How do I close the sim-to-real gap?), and artificial intelligence (when and how to express empathy). Finally, by comparison with CAD/CAM/CAE, simulation in robotics poses unique and truly multidisciplinary challenges, some of them summarized in this document. While established commercial CAD/CAM/CAE solution providers, e.g., MSC.ADAMS, Siemens, Dassault Systèms, etc., are anticipated to gradually address the needs of the robotics community, our near-term hopes are pinned on nimbler and more focused platforms, e.g., Bullet (25), Chrono (36), DART (26), Drake (55), MuJoCo (35), ODE (24), and SOFA (56). These platforms are by and large open source; are plugged deeper into the research community; and have the flexibility to rapidly translate modeling, numerical methods, sensor simulation, graphics, and emerging hardware architecture breakthroughs into advances that amplify the role that simulation plays in robotics.

## Conclusions

Simulation is not a goal in itself but a means to an end. This contribution outlines some of these ends and barriers that need to be overcome for simulation to deliver on its potential in robotics. We believe that undertaking a focused, coordinated, and sustained research and software development effort in this area is timely. In the process, the simulation community will have to address research questions at the intersection of computer science, mechanical engineering, electrical engineering, cognitive science, and applied mathematics. Without a systematic, guided, and all-encompassing effort, advancing the state of the art will be marred by trial-and-error detours. The pressing question is how to jump start a robotics-simulation cross-pollination process that would speedily transition the effort from the “academic debate” phase into a “simulation-enabled building and demonstration of technology” phase. This transition can be catalyzed by a sustained decade-long financial commitment, which would ensure funding for cross-disciplinary efforts that promote collaboration, competition, and compilation of open repositories of validated models and source code. As witnessed in the aerospace and automotive industries, the paradigm shift to digital, while manifestly impactful, took decades to coalesce. Learning from this experience, the hope is that simulation will lead to breakthroughs in the design of smart robots in a matter of years rather than decades.

## Supplementary Material

Supplementary File

## Data Availability

All study data are included in this article and *SI Appendix*.
