# Supplementary material for: On the use of simulation in robotics: Opportunities, challenges, and suggestions for moving forward
Source: Proc Natl Acad Sci U S A. 2020 Dec 28;118(1):e1907856118. doi: 10.1073/pnas.1907856118 (PMC7817170; doi:10.1073/pnas.1907856118)
Supplement: Supplementary File [file pnas.1907856118.sapp.pdf]

Modeling and Simulation in Robotics Workshop

Thoughts on opportunities, challenges, and next steps from attendees gathered prior to workshop

www.pnas.org/cgi/doi/10.1073/pnas.1907856117

Modeling and Simulation in Robotics Workshop

Attendee Pre-workshop Thoughts

How Simulation can Help Robotics?

- Cheaply generate **large amount of training data** with ground truth realistic perception
- Large scale **debugging and testing**
- Experiment with **Re-enforcement learning** without crashing real vehicles
- Seamless deployment of code from **simulation to real robot**
- Establish **common platform to share algorithms and data**, compare and contrast
- Efficient **inference via simulator during runtime**
- **Shared grounding** across multiple tasks and modalities

Simulation-centric Robotics

Key Bottlenecks

Virtual Environments

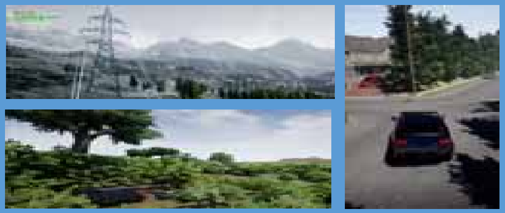

Enabling
Real-World AI
with Simulation

Sensor Suite Ecosystem

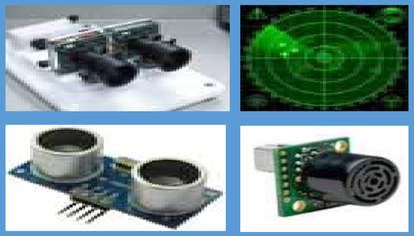

Cloud Compute,
ML Tools and Data Support

SIMULATOR

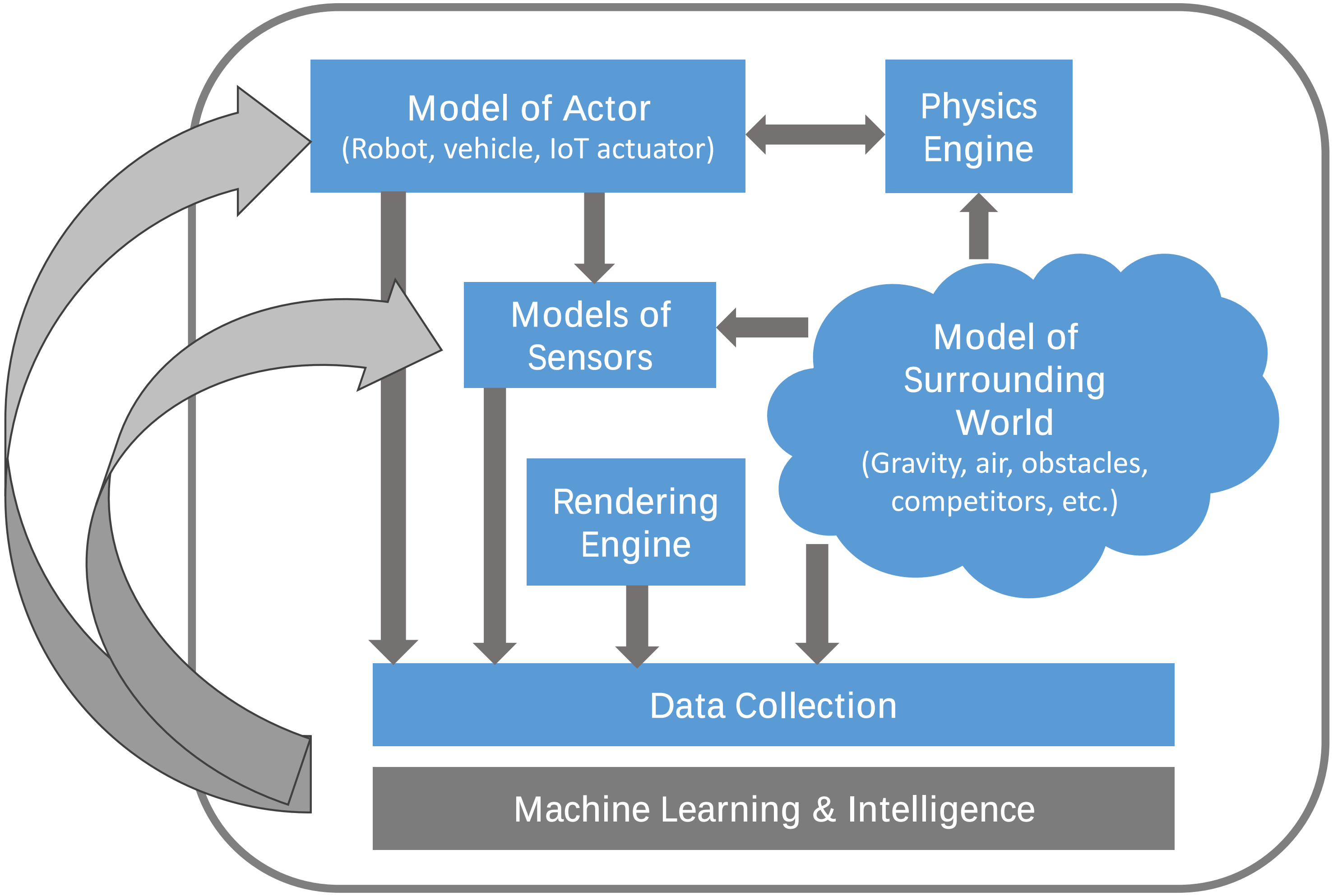

Open-Sourcing / Sharing is the key

We are trying to create reality in the virtual world.

Need to combine forces…

Modeling and Simulation in Robotics Workshop

Attendee Pre-workshop Thoughts

Opportunities

- Simulation is more scalable because it is faster than real time and multiple simulations can run in parallel.
- Simulation is safer than real robot.
- Running simulation is more cost efficient than running real robot experiments.
- Robotic controllers can be learned automatically in simulation.
- A large amount of recent work of reinforcement learning has achieved impressive results in simulation.

Challenges

- Reality gap: a policy learned in simulation does not work on real robot.
- System identification is a manual and tedious process.
- Many important aspects of robotics, such as vision system, deformation dynamics, human interaction, etc., are difficult or time consuming to simulate in high fidelity.
- It is almost impossible to simulation all situations of the real world.

Roadmap

- Develop robotic benchmarks (OpenAI gym on real robots).
- Open-source simulation and hardware platform.
- Build more rich simulation environments: support publications of simulation datasets: digitization of our world (3D maps, indoor scans, etc.).
- Gain better understanding of the reality gap and prioritize its causes.
- Automatic system identification and data-driven physics simulation.

Modeling and Simulation in Robotics Workshop

Attendee Pre-workshop Thoughts

Understanding Opportunities

- Solid (soft and rigid) body simulation
  - Use of models that capture relevant physics of robotic systems with contact accurately, lead to more accurate state estimations and therefore controllers with higher fidelity.
  - Develop robots with greater capability for locomotion and dexterous manipulation

Reality Check

- Users don’t know which model to use or how to tweak them when they fail.
- We need model validation physical against benchmark systems.

Call to Action!!

- We need to identify domains of applicability of various simulation tools – similar to suites of ODE solvers – for example, ChronoEngine is best for problem class A and MuJoCo is best for problem class B.

Modeling and Simulation in Robotics Workshop

Attendee Pre-workshop Thoughts

Understanding Opportunities for Modeling/Simulations

[a time to dream]

- Enable testing/debugging of actual control algorithms/software (make simulation control identical to robot control)
- Much faster/safer for exploring learning/optimization algorithms
- Massively parallel exploration
- Useful for non-experimentalists (although deceiving)
- Transfer learning from simulation to robot
- Create massive training data from simulation and variations of simulations (simulate sensors, environments, robots)
- Transfer learning between different simulators
- Close to realistic physics in simulator -- how much is needed?
- Learning high level feature representations/embeddings from simulator to bootstrap features/embeddings for real robots?
- Transfer learning between robots of different morphologies
- Discover an abstract high level representation that generalizes across robots of different morphologies

What's Stopping Us from Getting There

[the reality check]

- General high fidelity physics simulators for robots in arbitrary environments
- Mismatch between real robot and simulators needs methods to
  - Patch mismatch by additional learning layers
  - Combine simulation and real robot data with appropriate "weighting"
  - Abstraction towards a "general robot feature vector" (do not know what this could really be)
- Tradeoff between computational speed and fidelity of simulation
  - How much does closed loop control allow sacrificing simulation accuracy
  - How accurate can we simulate/model the environment anyways?
- Make it as easy as possible to setup simulation/models – easy sharing of "plug-ins" would be great
- Software engineering effort: it appears that only a bigger well-funded (non-academic) entity can create the right tools

Pragmatic Suggestions for Moving Forward

[what funding organizations, the robotics community, other vested parties can/should do]

- Convince people to work on a common tool, abandoning their own tools
  - This is real hard, as many robotics labs have so much investment in their simulation/modeling tools
- Fund people to come together with such simulation/modeling tools
- Support by research industry and national funding agencies: open source, software engineering, maintenance, documentation, support, simulator/models for all robots in the market
- Fund multiple competing efforts (similar to TensorFlow, PyTorch, etc.), and let people choose what fits best
- Theory of physical simulations? Done, boring, to be further developed?

Additional Thoughts/Comments You Might Have

- Compare tools available, try to rank pros and cons
- Complexity of worlds people wish to model/simulate
- How important is graphics visualization?
- Avoid ROS-like software library dependencies and OS release dependencies
- What can academic institutions contribute? E.g., are such modeling/simulation efforts suitable for Ph.D. students (worth a Ph.D. thesis)?
- Are federal funding agencies willing to fund such “system/tool development” efforts?

Modeling and Simulation in Robotics Workshop

Attendee Pre-workshop Thoughts

Understanding opportunities (a time to dream)

- Moore’s Law: cheaper, faster, abundant compute power
  - Computation as the feature driver
  - More and more advanced software intelligence (adaptive, autonomous) in robotics
  - Fast, accurate, realistic simulations, sufficiently rich environment models are possible
- Metcalfe’s Law: fast and reliable wireless network/internet connectivity
  - Novel distributed architectures increasingly becoming possible
    - Example: flying quadrotor with a router and a camera beaming internet connectivity to a ground robot and beaming video of the ground robot back to a base station
  - Connected adaptive and collaborative autonomous systems
    - Share what one robot learns with the entire fleet/team, collaborate with other robots and humans
- Post-deployment system integration
  - Modeling and simulation a key platform to try out the actual functionality
    - To develop, test, perfect new functionality
    - Train machine learning programs with synthetic data

What’s stopping us from getting there (a reality check)

- Many open questions
  - Adaptivity
    - How to limit learning only to safe behaviors?
    - How to avoid interpretation edge cases?
  - Autonomy
    - How to model non-trivial yet safe interaction with humans?
    - How to ensure sufficient richness in environment models (available or learned via SLAM)?
      - SLAM: Simultaneous localization and mapping
  - Connectivity
    - How to interpret data safely (in presence of malicious entities)?
    - How to adapt at run-time to degraded network connectivity? Impact on the ensemble performance?
  - Collaboration
    - How to gracefully enter and exit an ad hoc collaboration?
    - Runtime distributed consensus, conflict resolution, and control synthesis
    - System vs. ensemble tradeoffs (“price of anarchy”)

Pragmatic suggestions for funding organizations and the Robotics community

- Application-specific researcher collaboration networks
  - Bring together expertise from various areas working towards a common application
- Common or interoperable software ecosystems and toolchains
- Repositories for sharing
  - algorithms, tools, realistic datasets
  - benchmarks, challenge problems
- Access to hardware and compute platforms, testing facilities as well as models of such facilities
- Metrics of success for research projects
- Grand challenges

Modeling and Simulation in Robotics Workshop

Attendee Pre-workshop Thoughts

Military Medical Purpose:
To support medical care delivery in dispersed and complex environments through futuristic technologies:

Why?
- Unique need to push trauma surgeon and physician level clinical expertise and capability to remote geographic locations and provide field care for long periods without more medical personnel, resources, or opportunities for medical evacuation
- Unique challenge of limited network capabilities and bandwidth dedicated to medical sustainment which will likely remain or worsen in military settings in remote geographic locations
- On-demand Simulation required to respond to current and geographic specific threats

Medical Robotics and Autonomous Systems
- Develop semi-autonomous/autonomous robotic surgical, emergency procedures and ICU capabilities for use in field care

Virtual Health
- Exporting medical expertise to the deployed medical forces and improved clinical outcomes for the wounded, ill and injured service members

Medical Device Interoperability
- Technical architectures and standards to support autonomous medical devices interaction

Medical Simulation and Training
- Increase patient safety and quality of care through simulation-based technologies

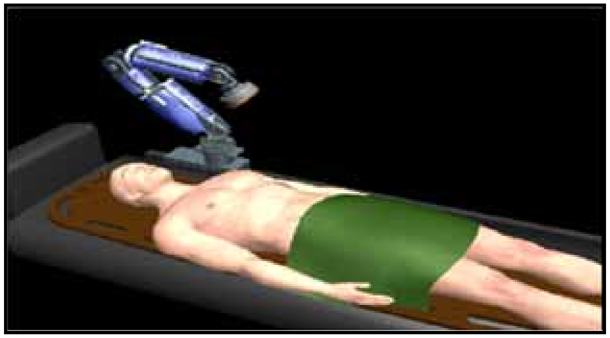

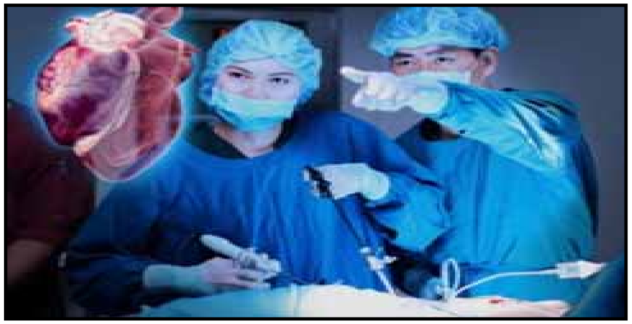

Understanding Opportunities

Research Questions

- ❖ What models, approaches, and ***existing*** concepts can be leveraged effectively to meet basic medical research objectives?
  - *Autonomous, closed loop control for diagnosis and treatment in critical care to reduce preventable harm*
  - *Unmanned systems for pre-hospital transport/medical resupply*
  - *Tele-surgical robotics in semi-autonomous or autonomous operation*
- ❖ What models and concepts may differ to meet patient safety/regulatory requirements?
- ❖ Can models and simulations identify, exploit or mitigate uncertainty and risks (known unknowns and unknown unknowns of the future environment)? How will we know or test?

What holds us back?

- Medically relevant, standards based reference architectures and conceptual frameworks for robotics (open source, open standards, reference implementations, etc.)
- Interoperability of medical devices, systems, data, platforms (systems of systems)
- Cybersecurity, secure platform requirements for connected medical devices and connected systems of critical care (embedded systems)
- Appropriate test environments

Collaboration Opportunities

- Development of Reference Architectures, Models, Running Code
  - Int'l and Industry Standards Development for Medical Applications in Autonomous Systems/Robotics
  - Public/Private Partnerships with Standards Development Organizations, Industry, Interagency Work Groups on Frameworks Development
  - Conformance, compliance, certification testing regimes
- Medical Simulation Environments/Platforms/OSs

Modeling and Simulation in Robotics Workshop

Attendee Pre-workshop Thoughts

Understanding opportunities

- Create integration points for simulation with existing CAD software
- Simulation as continuous integration tool
- Cloud-based simulation to support parameter search, Monte-Carlo experiments, predictive control
- Integration of simulation with common software development tools (IDEs, debuggers)

What’s stopping up from getting there

- Existing simulation tools do not seamlessly fit into common engineering work flows.
- Simulation uptake hampered by lack of understanding of what simulation can do and why simulation is important.
- More users across domains drives feature requirements.
- Modeling sensors and actuators accurately is difficult.
- Large scale simulations (spatial size and complexity) quickly hit computational limitations.

Pragmatic suggestions for moving forward

- Reduce learning curve, and improve accessibility through user testing & studies, and identify the needs of mechanical, electrical, and software engineers.
- Create guides to choosing the right simulator, and tutorials on how to use each simulator.
- Crowd source model and environment creation.
- Establish modeling format(s) that support sharing of resource across different simulators.
- Look toward distributed simulations to support large scenarios.

Modeling and Simulation in Robotics Workshop

Attendee Pre-workshop Thoughts

Understanding Opportunities

[a time to dream]

- The control of contacts and friction remain a fundamental issue yet great opportunity for robotics manipulation and locomotion. I would love to have models that go beyond rigid contact models and yet are simple enough to be used in receding horizon control. This would allow the control of complex non-rigid mechanisms and objects.

- If we could generate large amounts of sensory-motor data in simulation that would match the (multi-resolution) dynamics, noise, softness, etc. of sensors and actuators during complex behaviors including frictional contacts, we could systematically study the sensory-motor space of robotic manipulation and locomotion, i.e. find relevant sub-manifolds, study the effect of sensor placement and redundancy on performance, softness, etc. => make robotics a data science.

- We could use this data to learn: 1) control policies and 2) simple yet rich predictive models for receding horizon control that would directly transfer on real robots

What’s Stopping Us from Getting There

[the reality check]

- Contact models are either computationally heavy which prevents the ability to generate large sets of data quickly or they are (too) simple phenomenological models that fail to capture reality (no ability to exploit complex interactions, softness, etc.)

- Simulation rarely comes “out-of-the-box” and necessitates expert knowledge to produce results that are physically plausible and transferable to a real machine (optimization or learning algorithms tend to exploit any physical inconsistency in the simulation). Simulation “hates” flexibilities and softness while robotics should be soft and exploit softness.

- Simulation results (even with expert knowledge) necessitate additional work to get things to work on real, complex machines. It is very difficult to perform system identification for discontinuous dynamics (i.e. to calibrate simulators).

Pragmatic Suggestions for Moving Forward

[what funding organizations, the robotics community, other vested parties can/should do]

- What about using simulators from other fields of mechanics / physics that are more precise, coupled with cluster/cloud computing? Can this scale?
- Can we use real-world data to “calibrate” our simulators? What would be a roadmap to do this systematically?

Modeling and Simulation in Robotics Workshop

Attendee Pre-workshop Thoughts

thoughts of how computer modeling and simulation can help robotics

- Better design and requirements generation for future robotic systems
- High speed simulation game changing for decision and path planning.
- Reachability, Viability set calculation of trajectories and behaviors.
- Fast and cheap verification.
- Reduced power from computing.
- Will help in co-design optimization

the reality check

- Robotics systems are complex and their simulation requires large numbers of computation.
- Computation is not free and it comes at the sacrifice of high power and heat dissipation requirements (GPUs on dynamic systems).
- Complexity of simulation methods does not scale well with complexity or number of system components.
- The majority of methods of simulation may have non-deterministic time in completion (i.e., it is unknown how long the simulation will take to complete).

pragmatic suggestions for moving forward

- Stop relying on unlimited computing power and consider it in the design of simulation methods.
- Look at methods of better model abstraction and use of mathematics to understand what you loose in choosing a specific approximation.
- Decide on what is important to know about the system and see if it can be analyzed through low order invariants without simulating a high accuracy model.
- Always strive to make simulation methods as close as possible to deterministic-time in solution (for embedded systems mostly).
- Look toward low loss fast computation processor technologies.

Modeling and Simulation in Robotics Workshop

Attendee Pre-workshop Thoughts

Understanding Opportunities

[a time to ~~dream~~ complain]

- World's first unplugged legged runner
  - U. Saranli, M. Buehler, and D. E. Koditschek, "RHex: A Simple and Highly Mobile Hexapod Robot," *The International Journal of Robotics Research*, vol. 20, no. 7, p. 616, 2001.
  - J. D. Weingarten, G. A. D. Lopes, M. Buehler, R. E. Groff, and D. E. Koditschek, "Automated gait adaptation for legged robots," in *Robotics and Automation, 2004. Proceedings. ICRA'04. 2004 IEEE International Conference on*, 2004, vol. 3, pp. 2153–2158.
- World's first legged vertical climber
  - M. J. Spenko, J. A. Saunders, G. C. Haynes, M. A. Cutkosky, A. A. Rizzi, R. J. Full, D. E. Koditschek, "Biologically inspired climbing with a hexapedal robot," *Journal of Field Robotics*, vol. 25, no. 4–5, pp. 223–242, 2008.
  - G. C. Haynes, A. A. Rizzi, and D. E. Koditschek, "Multistable phase regulation for robust steady and transitional legged gaits," *The International Journal of Robotics Research*, vol. 31, no. 14, pp. 1712 – 1738, Dec. 2012.
- Spent a ***lot*** of time tuning parameters ☹

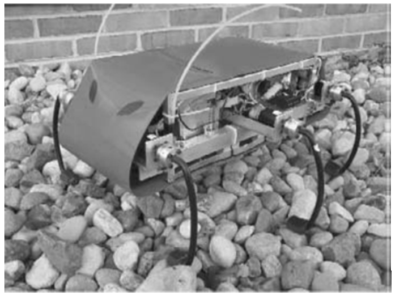

Table 1. Summary of Published Performance Reports: Hexapedal Robot

Name	L (m)[b]	M (kg)[b]	V (m/s)[b]	V/L
Case Western Robot II (Chiel et al. 1992)	0.5	1	0.083	0.16
Dante II (Bares and Wettergreen 1999)	3	770	0.017	0.006
Atilla[a] (Angle 1991)	0.36	2.5	0.03	0.083
Genghis[a] (Angle 1989)	0.39	1.8	0.038	0.097
Adaptive Suspension Vehicle[a] (Pugh et al. 1990)	5	3200	1.1	0.22
Boadicea (Binnard 1995)	0.5	4.9	0.11	0.22
Sprawlita (Clark et al. 2001)	0.17	0.27	0.42	2.5
RHex[a]	0.53	7	0.55	1.04

a. Power autonomous.
b. L = body length, M = robot mass, V = maximum speed.

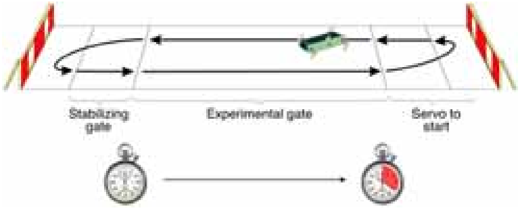

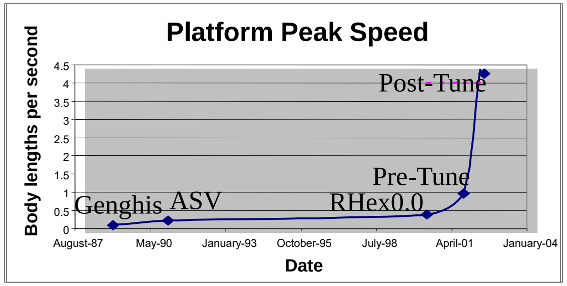

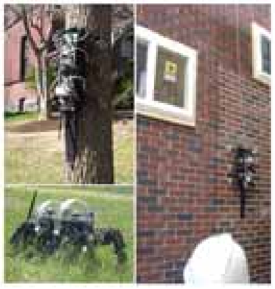

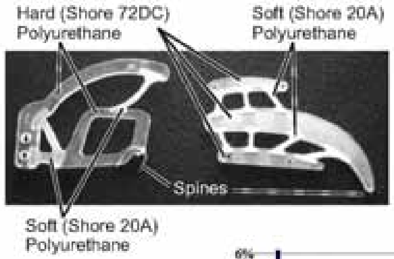

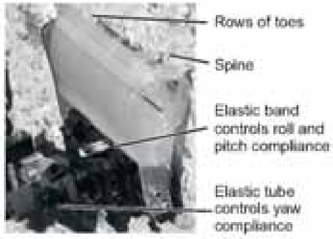

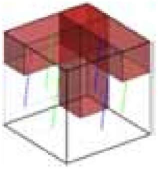

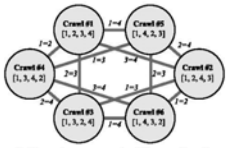

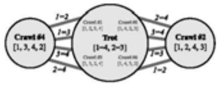

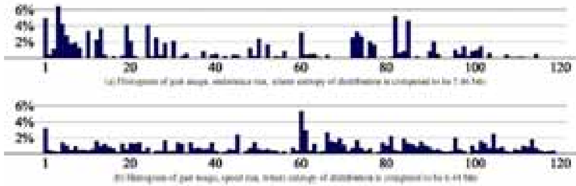

What’s Stopping Us from Getting There

[the embodiment ~~reality~~ check] *“Simulations are doomed to succeed”* – *Louis L. Whitcomb, c. 1988*

- Switched Contact Environments (Hybrid Dynamics)
    - A. M. Johnson and D. E. Koditschek, “Toward a vocabulary of legged leaping,” in *Robotics and Automation (ICRA), 2013 IEEE International Conference on*, 2013, pp. 2568–2575.
    - A. M. Johnson, S. A. Burden, and D. E. Koditschek, “A hybrid systems model for simple manipulation and self-manipulation systems,” *The International Journal of Robotics Research*, vol. 35, no. 11, pp. 1354–1392, 2016.

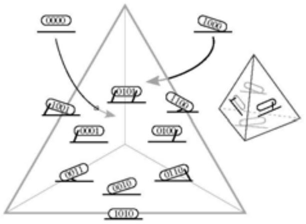

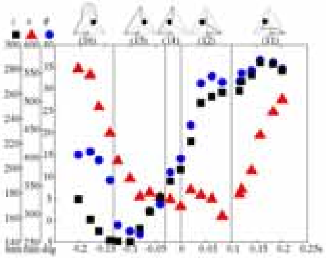

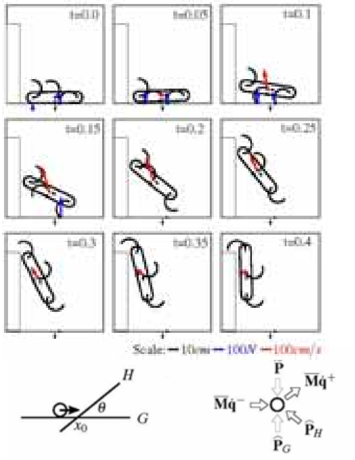

- Single Contact Environments (Granular Mechanics)
    - C. Li, P. B. Umbanhowar, H. Komsuoglu, D. E. Koditschek, and D. I. Goldman, “Sensitive dependence of the motion of a legged robot on granular media,” *Proceedings of the National Academy of Sciences*, vol. 106, no. 9, pp. 3029–3034, 2009.

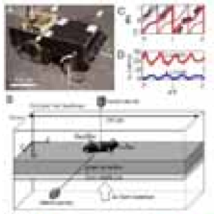

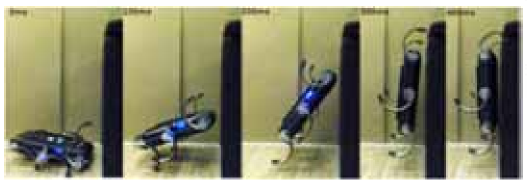

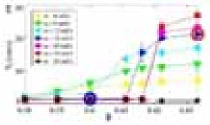

- No-Contact Environments (Rigid Body Mechanics)
    - L. L. Whitcomb, A. A. Rizzi, and D. E. Koditschek, “Comparative experiments with a new adaptive controller for robot arms,” *IEEE Transactions on Robotics and Automation*, vol. 9, no. 1, pp. 59–70, 1993.

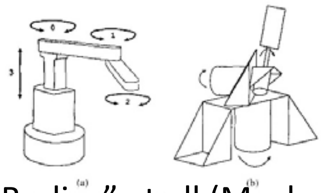

$$m\ddot{x} = f(x, \dot{x})$$

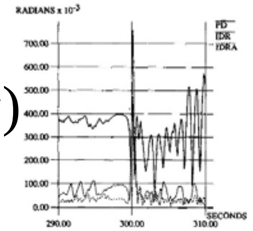

- No “Bodies” at all (Mechanical Circuits)

“Each part will typically participate in or contribute to several functions … (solid, electricity, heat …) …therefore highly coupled …”

- D. E. Whitney, “Why mechanical design cannot be like VLSI design,” *Research in Engineering Design*, vol. 8, no. 3, pp. 125–138, 1996.

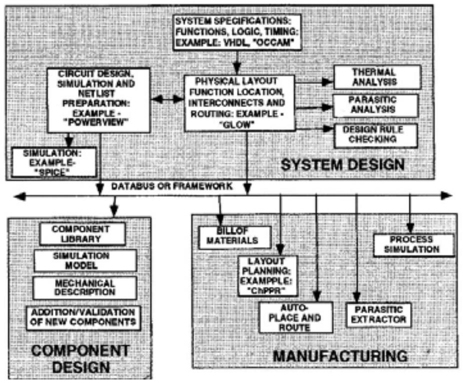

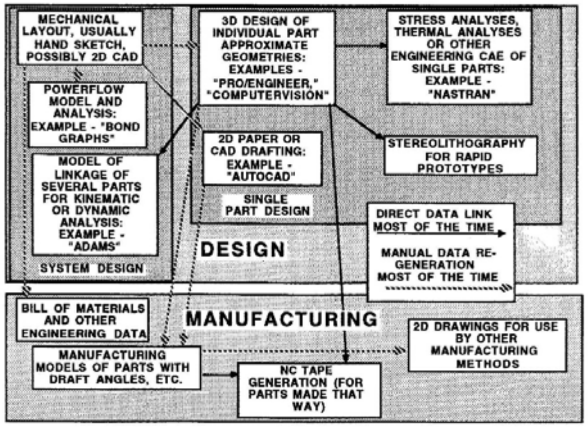

Pragmatic Suggestions for Moving Forward

[what funding organizations, the robotics community, other vested parties can/should do]

- Physical Testbeds & Benchmark Tasks
- “Forced” collaboration between modelers, builders, controllers and learners
- Careful study and recapitulation of past history and neighboring domains
  - Spice -> analog circuits triumph
  - VLSI -> computer engineering triumph
  - Analog (“neuromorphic”) Systems -> still awaiting scalable design theory
  - Synthetic biology (harder still than robotics)

Additional Thoughts/Comments You Might Have

- Add here any thoughts that perhaps don’t belong to the previous three slides
  - Fundamental problem: Whitney’s Dilemma (embodiment entails multiple coupled flows)
    - robotics lacks appropriate abstractions to organize structure/function hierarchies and compositions (*cf.* much simpler CS setting of gates/bits -> ops/registers -> programs/machines)
    - abstractions delimit “levels” of appropriate modeling detail & accuracy
    - abstractions govern model/controller/behavior composition

- Include here any suggestions that you think might make our meeting more productive
  - thanks for taking on this fundamental problem!

Modeling and Simulation in Robotics Workshop

Attendee Pre-workshop Thoughts

Understanding Opportunities
[a time to dream]

- Use physics simulation to create infinite number of scenarios and physical designs for training robot control policies.

- Use physics simulation to model human behaviors for robot-human interaction through physical contacts.

- Use physics simulation to provide infinite number of training samples for state estimation or enhancement.

What’s Stopping Us from Getting There
[the reality check]

- Lack of computation power (algorithms mostly CPU-bound)
- Lack of good system identification methods
- Lack of good methods to combine data with analytical models

Pragmatic Suggestions for Moving Forward

[what funding organizations, the robotics community, other vested parties can/should do]

- Grand challenge: Simulating X!
- Need to involve more graphics people in passive simulation community
- Need to develop benchmarks for simulators

Modeling and Simulation in Robotics Workshop

Attendee Pre-workshop Thoughts

Understanding Opportunities
[a time to dream]

- Risk assessments and safety intervention for robot-related human injury incidents
- Improvement of collaborative and co-existing robot systems
- Design of human-robot interface and communication systems
- Development of training for humans interacting with industrial or service robots

What’s Stopping Us from Getting There

[the reality check]

- Limited knowledge of human behaviors and cognitive performance interacting with robots

- Limited science-based requirements and thresholds for safe human-robot interaction

Pragmatic Suggestions for Moving Forward

[what funding organizations, the robotics community, other vested parties can/should do]

- Research on biomechanics, anthropometry, and human behaviors associated with robotics technology
- Improvement of human models
- Improvement of simulation technology for human subject experiments (e.g., virtual reality)

Additional Thoughts/Comments You Might Have

- Expanding applications of modeling and simulation for small and medium-sized businesses using robotics

Modeling and Simulation in Robotics Workshop

Attendee Pre-workshop Thoughts

How can modeling and simulation help robotics?

- Virtual prototyping
  - Cf. EDA
- Testing fault conditions
  - Cf. Prototyping
- Reduce hacking
- Enable in-the-field updates

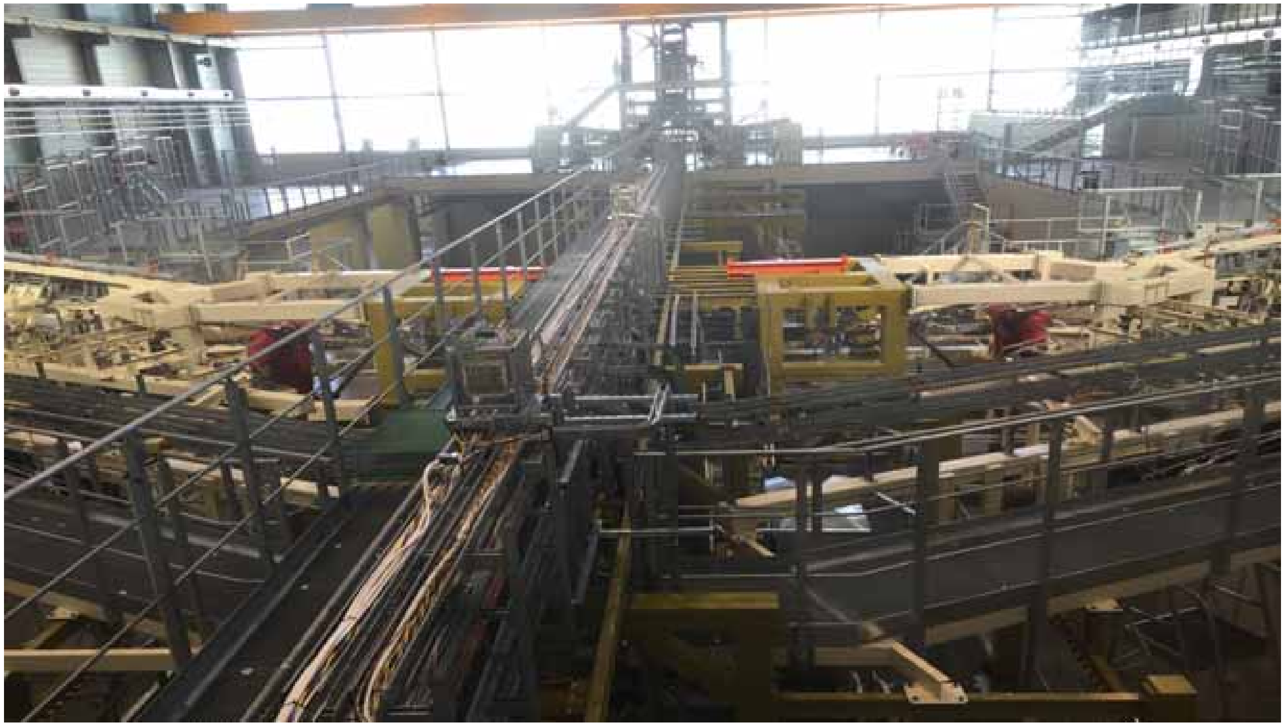

“Iron Wing” model of an Airbus A350, Toulouse, France

What holds us back?

- Confusing the map and the territory
- Confusing scientific vs. engineering models
- Failure to recognize the value of deterministic models
- Comodeling cyber and physical
- Prototype-and-test (hacking)

In *science*, the value of a *model* lies in how well its behavior matches that of the physical system.

In *engineering*, the value of the *physical system* lies in how well its behavior matches that of the model.

A scientist asks, “Can I make a model for this thing?”
An engineer asks, “Can I make a thing for this model?”

Pragmatic steps

- Research cyber-physical modeling
- Invest in open-source modeling tools
- Strive for *simple* vs. comprehensive models
- Layer models
- Compositional abstractions

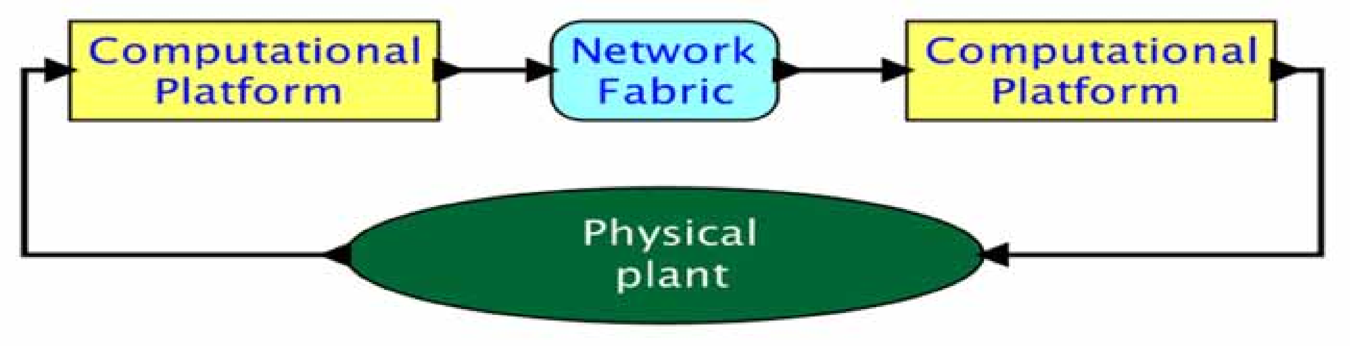

Best-of-class cyber models and best-of-class physical models are incompatible today.

Modeling and Simulation in Robotics Workshop

Attendee Pre-workshop Thoughts

Understanding Opportunities
[a time to dream]

How can computer modeling and simulation help robotics ?

- Help a better design of robots powered by multi-physics behavior: chemistry, electric, biophysics, new materials, deformations,…

- Provide "a virtual & realistic ground truth" for all possible kind of optimization (planning, machine learning, shape optimization, …) and more particularly when ground truth data are difficult to obtain/recreate: **surgical robotics**, search and rescue scenarios, space robotics...

- Computerize the process from definition of the problem to automatic control of a robot in the environment (design, simulation of the environment, generation of the control laws)

What’s Stopping Us from Getting There

[the reality check]

- Help a better design of robots powered by multi-physics behavior: chemistry, electric, biophysics, new materials, deformations,…
  - Complexity: from the user point of view, just to create the adequate simulation, in particular for the choice (not automatic) of what should be modeled or not… and how...
  - When adding too many features on a simulation platform, it becomes rapidly too complex, too difficult to maintain and validate.
- Provide “a virtual & realistic ground truth” for all possible kind of optimization (planning, machine learning, shape optimization, …) and more particularly when ground truth data are difficult to obtain/recreate : **surgical robotics**, search and rescue scenarios, space robotics...
  - Lack of realism: optimizations have the tendency to exploit the failures of the simulations
  - Surgical robotics: particular difficulty to get realistic behaviors of soft-tissues and interaction with surgical instruments. A lot of existing results but we are not there yet…
- Computerize the process from definition of the problem to automatic control of a robot in the environment (design, simulation of the environment, generation of the control laws…)
  - Poor integration of ”real world problems” (sensor noise, actuator limits, realistic friction...) inside the simulations
  - Same difficulty (mentioned above) of having “too complex” software

Pragmatic Suggestions for Moving Forward

[what funding organizations, the robotics community, other vested parties can/should do]

- Make the community “robotic simulation” more structured/visible ?
  - More tutorials / demos / teaching / workshop on modeling and simulation for robotics in the main robotics conferences
  - Today not sure that “simulation” and ”modeling” is seen as a very important topic by the robotics community (today, everyone is talking about modeless approaches…)
- Specific for surgical robotics: There is maybe a possible synergy on ”surgical simulation”: what has been originally done for training residents could today be used by machine learning for doing automatically some parts of the surgery.
- Develop means of validation / comparison between existing simulation software
  - Build validation test benches
  - Organize a contest ?

Modeling and Simulation in Robotics Workshop

Attendee Pre-workshop Thoughts

Understanding Opportunities

[a time to dream]

- Recent advances in deep networks coupled with reinforcement learning offer exciting new possibilities for autonomous robotic systems to learn in simulation, and carry the learned skills across the reality gap to the real world. Can such systems continue learning in the real world with a comparative dearth of data (as compared with the simulated world)?

- Generative Adversarial Networks have proven effective in generating realistic images and a few specific other types of potential sensor data for robots. Can GANs be used to generate more sophisticated realistic data environments for training and testing robots, such as simulated scenarios involving realistic physics?

- A key component on an "intelligent" system is that it must be able, at some level, to learn from its mistakes. But on-line learning presents a challenge for verification of autonomous systems. How can we design systems that can both learn from their mistakes and be verifiable?

What’s Stopping Us from Getting There

[the reality check]

- Many of the most successful techniques (such as deep Q learning) rely upon an assortment of ad hoc techniques to avoid getting stuck. Also, the design of deep networks themselves appears to be extremely ad hoc. We need a more principled approach to deep learning, and to reinforcement learning with deep networks.

- We need a better understanding of what types of data representations (or exemplars) a GAN can model, and what types it cannot. Also, we need to better understand the brittleness of GAN *G-network* models.

- Approaches to verification of autonomous systems are in their infancy. Approaches to verification for systems that learn (mostly offline) are in their infancy. Approaches to verification of autonomous robotic systems that learn on-line are essentially non-existent. In general we lack principled and effective means for verification of autonomous systems, and the situation is even worse for autonomous systems that learn.

Pragmatic Suggestions for Moving Forward

[what funding organizations, the robotics community, other vested parties can/should do]

- Invest in more research in high-fidelity simulation, deep reinforcement learning (RL), and training systems (through RL) that can cross the reality gap. Funding agencies may include NSF, NIST, DoD (DARPA, ONR, ARL, AFRL, OSD), DOE, NASA.
- Invest in more research in applying to GANs in a wide variety of modeling tasks. Funding agencies may include NSF, NIST, DoD (DARPA, ONR, ARL, AFRL, OSD), DOE, NASA.
- Develop verification techniques for autonomous systems in parallel with industry and military standards and guidelines. Extend these verification techniques first to systems that learn offline (data collected on-line may be used for offline learning in simulation). Next, extend these verification techniques to on-line learning, perhaps through parallel on-line simulation or look-ahead capabilities. (NIST, DoD, NASA)

Modeling and Simulation in Robotics Workshop

Attendee Pre-workshop Thoughts

Understanding Opportunities

[a time to dream]

- Bio-inspired robots will be able to help us in many important applications if they can move well in complex terrain, e.g.:
  - Move through building rubble filled with irregular debris to do search and rescue
  - Move through forest floor filled with leaf litter, fallen branches, rocks and boulders, etc. to do environmental monitoring
  - Move beyond flat Martian surface (which rovers are limited to) and into rocky Martian terrain to study interesting planetary science, e.g. geology, chemistry, evidence of life, etc.

- Recent research in the new field of terradynamics has demonstrated that, once rigorously validated by experiments, new models of locomotor-terrain interaction of complex terrain and simulations based on such models are powerful at predicting locomotion in complex terrain
  - Enable robot design prediction to move well in complex terrain
  - Enable robot feedback control in response to terrain changes

What’s Stopping Us from Getting There

[the reality check]

- Difficulty in writing down equations using dynamic systems theory approach for such complex locomotor-terrain interaction problems

- Lack of contact mechanics models and validated simulation tools for arbitrary shaped objects interacting with each other to make predictions of robot locomotion in complex terrain
    - e.g., a robot (or animal) with complex body/leg shape interacting with irregular building rubble
    - In such cases, we don’t know well how physical parameters like coefficient of restitution is defined or how to measure it when we are considering an object of arbitrary shape colliding with another one

- Lack of established experimental procedures to validate such models and simulations

Pragmatic Suggestions for Moving Forward

[what funding organizations, the robotics community, other vested parties can/should do]

- Closer interaction and collaboration between experimentalists and model/simulation tool developers
- More recognition from research community and more support from funding agencies on less thought about, but important, emerging topics
  - Much of terrain modeling so far has been on granular media or similar terrain that are relatively uniform and simple compared to lots of real world terrain (e.g., building rubble, forest floor, large rocks on Mars) which we also need robots to move through
- Closer interaction and learning from each other between researchers studying robot locomotion and researchers studying robot manipulation
  - Both require understanding of physical contact

Modeling and Simulation in Robotics Workshop

Attendee Pre-workshop Thoughts

Understanding Opportunities
[a time to dream]

- How do we build **trust** in simulators?
  - Improve the accuracy of their predictions -> Data-enhanced simulators.
  - Reduce our confidence in their predictions -> Stochastic simulators.

- Simulation makes it **easier** to design and optimize machines.
  - Reliable simulation will make it possible to design more complex robotic systems.
  - Affordable simulation will make it possible for more people to design robotic systems.

  If we have both, the sky is the limit.

What's Stopping Us from Getting There
[the reality check]

- Experience says that the models of frictional contact we use in robotics are noisy.
- Intuition says that, in practice, it is not possible to develop very accurate predictive models for frictional interaction. It would require knowing state and geometry very precisely (microscopic features).

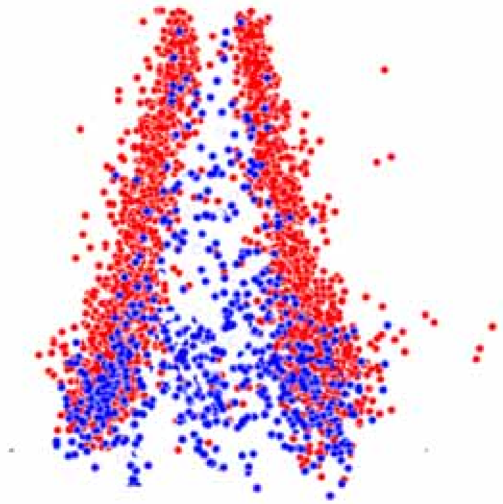

Experimental friction cone. (blue) Contact forces that lead to sticking. (red) contact forces that lead to sliding. It looks like a cone, but very noisy.

Pragmatic Suggestions for Moving Forward

[what funding organizations, the robotics community, other vested parties can/should do]

- Noise should be an integral part of a simulator. We should establish best practices for where and how to introduce noise in a simulation.
- Simulators should be validated against standard (stochastic) benchmarks.
- We should have easier access to professional simulators and stop thinking that using untested simulators is a good idea.

Modeling and Simulation in Robotics Workshop

Attendee Pre-workshop Thoughts

Understanding opportunities

- Modeling plays a critical role in several areas of robotics besides simulation
  - System design
  - Dynamics and control
  - Autonomous planning & execution
  - Dealing with uncertainty
  - Handling run-time structural changes
  - Real-time adaptation
  - Human interfaces
  - …
- For simulations, the focus is on the model doing one thing, i.e. state propagation well, with emphasis on fidelity and speed.
- In contrast, for robotics, modeling solutions need to be versatile and capable of supporting a broad range of queries from kinematics, dynamics, controls, planning, resource usage, geometric, constraint satisfaction etc. under varying conditions.
- Thus we need to think of modeling in the large for robotics - not just modeling for simulation - to truly address robotics needs.

What holds us back

- Modeling for robotic systems is challenging
  - Models can span multiple domains – dynamics, sensors, geometry, environment and interactions etc. and require broad expertise
  - Demands on fast & real-time computational performance
  - Complex physics and mathematics
  - Changing structure – constraints, interactions, varying scenarios
  - Uncertainty and incomplete knowledge of data
  - Reduced order models for control (ZMP, etc.)
  - …
- In the absence of a coherent approach, this leads to fragmented and point solution models for specific applications
  - End up with expensive and custom solutions, that are difficult to scale, mature, improve & share

Suggestions for moving forward

- Lack established curriculum to train personnel to work in this area
- Challenges are similar to system engineering – good modelers need to straddle multiple areas from software skills to expertise across multiple domains
- Currently modeling is viewed as a secondary capability to the main robotics engineering thrust and does not get the attention or talent to do it right
  - E.g., few sessions at ICRA in the area of modeling or M&S
- Need to consider embedded models for onboard software
  - Extra requirements in versatility and real-time responsiveness
  - Need to address collision free path planning, whole-body constraint, stability, estimation, constrained motion, precision needs, human interfaces etc.
- Define needs for different robotics domains so can develop & mature capabilities
  - While they are have a lot in common, they also have specialized needs
  - Focus on architectures that can help reuse and scale models (true reuse, not just forking)
- Develop benchmark problems to allow assessment of quality of solutions
- Need to develop modeling architectures that can be used for studies and research, but also have the breadth and depth to scale up for complex, real-life robotics applications

Modeling and Simulation in Robotics Workshop

Attendee Pre-workshop Thoughts

Understanding Opportunities
[a time to dream]

- Reduction of risk to workers in dangerous occupations

- Eliminate repetitive motion trauma and musculoskeletal overload

- Reduced fatalities and injuries from motor vehicle incidents

What’s Stopping Us from Getting There
[the reality check]

- Lack of trust (fear)
- Developing AI
- Adequate sensor technology

Pragmatic Suggestions for Moving Forward

[what funding organizations, the robotics community, other vested parties can/should do]

- Assure use of prevention through design (PtD) principles
- A priori risk assessment
- Thorough laboratory testing prior to deployment – avoid unforced errors

Additional Thoughts/Comments You Might Have

- Consider how VR simulations can help develop AI and the human-AI interface
- Consideration for integration of modeling and simulation into ISO pre-deployment standards

Modeling and Simulation in Robotics Workshop

Attendee Pre-workshop Thoughts

Understanding Opportunities
[a time to dream]

- Simulation is a way of “imagining the future” and learning or refining plans and actions without taking physical action.

- Simulation is a way of creating demand-driven data for offline and online learning

- Grounding simulation with reality would be a way of specializing simulation to immediate context.

What’s Stopping Us from Getting There

[the reality check]

- Simulations aren’t easily composable
- Simulations require us to fill in many many details which are unknown
- It is hard to choose the right tradeoff between fidelity and speed
- Ultimately requires lots of fine-tuning to get something to work

Pragmatic Suggestions for Moving Forward
[what funding organizations, the robotics community, other vested parties can/should do]

- Focus on highly composable dictionaries of objects, actions, movements, actors, etc.
- Dynamic complexity/fidelity changes as simulation progresses
- Exploit learning for both tuning and rendering in simulation
- Explore ways to couple simulation to reality on a continuous basis

Additional Thoughts/Comments You Might Have

- Add here any thoughts that perhaps don’t belong to the previous three slides

- Do one-slide lightening intros at the start – who we are and once thing we want to accomplish during the meeting

Modeling and Simulation in Robotics Workshop

Attendee Pre-workshop Thoughts

Understanding Opportunities

[a time to dream]

- It’s time to “democratize” on-time, on-demand medical expertise to every point of need across the entire military health system. This “dream” can be realized through the development of future generation human:robotic:autonomous systems.

- The foundational technologies exist today to begin the development of these capabilities to project specialized medical expertise and care to any point of need, at any time, through combination of human, robotic and autonomous systems.

- Modeling, simulation and visualization have the potential to accelerate the development of near-and-far term solutions for this capability. This capability will be greatly enhanced through the development of a unified, collaborative, multidisciplinary approach.

- These systems will also become a foundational tool (autonomous intelligent mentoring) for the initial, sustainment and readiness training of current and future military healthcare providers.

What’s Stopping Us from Getting There

[the reality check]

- A unified, collaborative, multidisciplinary medical modeling, simulation and visualization environment (MSVE) for future research, development, testing and evaluation is not truly realized at this time.
- A lack of objective human performance data regarding clinical procedural skills likely to be performed by robotic and autonomous systems.
- The need for exacting fidelity in computer vision sensing systems to allow for the identification of anatomic structures, medical devices and instruments in cases of normal (relative), abnormal and disrupted structures related to human illness and injury.
- Security, safe-guards, communications, connectivity, and the ability for complete autonomy of systems in low/no communication environments. Portability, power, and operational sustainment of suggested systems are also significant design challenges. Simply stated a significant amount of technology maturity and systems integration is still required.
- Policy, regulatory and trust are also significant considerations that must be addressed as technical research and development proceeds.

Pragmatic Suggestions for Moving Forward

[what funding organizations, the robotics community, other vested parties can/should do]

- Invest, design, develop and implement a unified, collaborative, multidisciplinary military medical MSVE for future medical robotics and autonomous systems development.
- Invest in the development of a curated library of objective measurement of human performance (3D time-space modeling of human performance) in healthcare to provide foundational “learning content” for future generations of robotic and autonomous systems.
- Develop highly-collaborative, multi-disciplinary research communities to pursue future research and development efforts.
- Engage the wider community to begin research priorities, policy, regulatory, and funding discussions for these future systems.

Additional Thoughts/Comments You Might Have

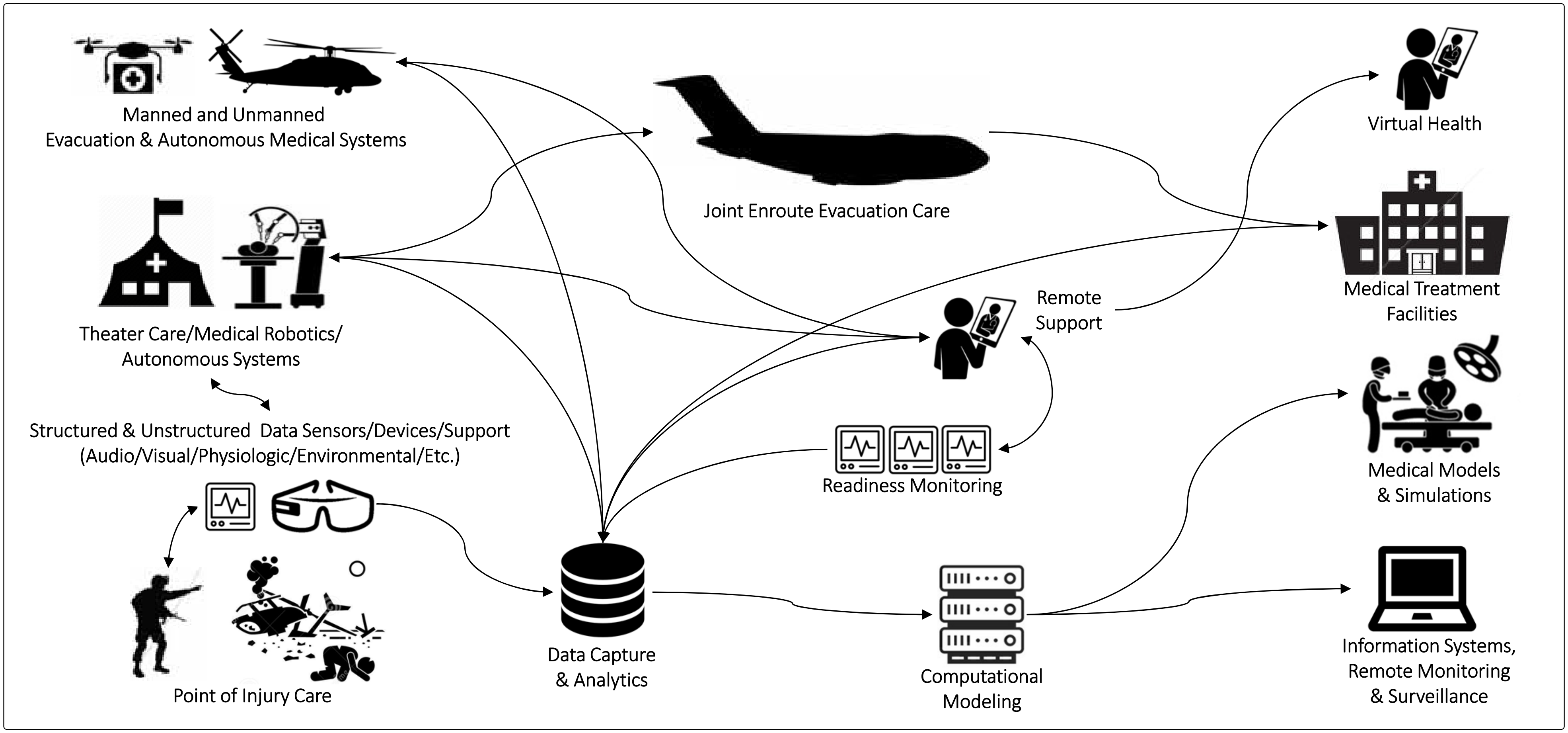

DRAFT Concept Diagram: Future Unified Tele-presence, Un-manned and Robotics systems Environment (FUTURE) for Military Medical Care

Modeling and Simulation in Robotics Workshop

Attendee Pre-workshop Thoughts

Understanding Opportunities

[a time to dream]

- Wanted: library of models of varying fidelity, with the following provided for each model:
  - Excellent documentation for how well (quantitatively) the model can capture phenomena (*a la* Wriggers' *Computational Contact Mechanics*)
  - Research pointing to how easy/difficult it is to identity or estimate system parameters for each model, (b) excellent documentation for what phenomena
  - Computational expenses for using the model in various contexts (e.g., estimation, simulation, control).

What’s Stopping Us from Getting There

- Focus on speed before accuracy
- No pipeline between libraries (CAD -> simulation -> deployment)

Pragmatic Suggestions for Moving Forward

- Using not-fully-vetted models ⇒ "Simulation is doomed to succeed"
  Fund creation of simple models that have been cross-verified and validated

- *Robust modeling and simulation software for robotics is a keystone technology*. Building it should be a national priority (think: NASTRAN, LAPACK)

- Academia needs to train students better to do the requisite numerical work.

Additional Thoughts

- The phrase “simulation-reality gap” needs to die.
  - Its use betrays a misunderstanding about how simulations are used
  - Simulation is limited by the fidelity of the models and the quality of the inputs
  - No one talks about a simulation-reality gap in the context of weather prediction

S. Zapolsky and E. M. Drumwright. Particle traces for detecting divergent robot behavior. In *Proc. HUMANOIDS*, 2016.

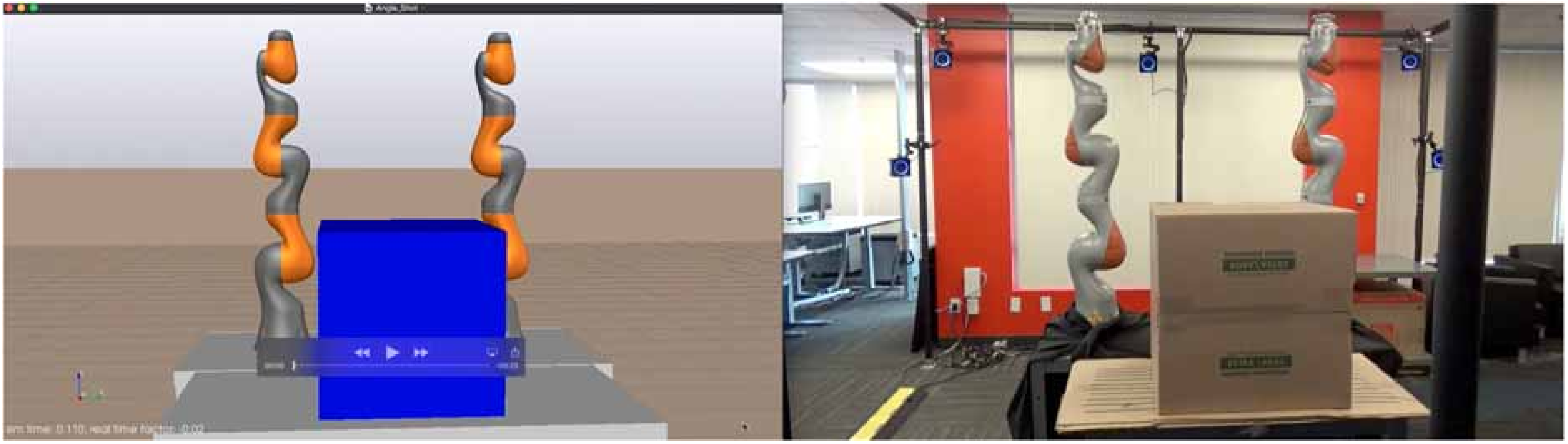

Modeling and Simulation in Robotics Workshop

Attendee Pre-workshop Thoughts

Understanding Opportunities

[a time to dream]

- Only path to on-demand statistically-relevant datasets that can begin to explore several variations of events (very relevant if you’re developing a closed-loop control technique rather than an open-loop perception technique)
- Provides a perfect platform for learning concepts that may be hard to manually encode, for example: spatial awareness and reasoning, social awareness, causality, etc.
- Make it sufficiently game-like to trick people into training your algorithm to be, e.g. spatially-aware, through imitation learning, inverse reinforcement learning, etc.
- Human-in-the-loop policy learning: identify edge case, drop several variations into simulation, turn teams of people loose on showing how to get out of trouble, use demonstrations to shape reward or seed stand-alone simulation and refine, iterate as needed

What’s Stopping Us from Getting There

[the reality check]

- Procedural scenario/environment generation (relevant questions: (1) how to generate scenarios at varying scopes/complexities; (2) when will generator be “sufficient” for allowing all possible scenarios that we may actually encounter; (3) is the generator “unbiased”)
- Making complex simulators (e.g. with dynamic environment, crowd simulations, adversaries) is really hard: lots of competing technologies fractured across a variety of simulators, technologies tend to be bimodal (stale former grad student code or proprietary black-box)
- User interactivity: simulator needs to act more like a game engine to make people comfortable interacting with it, but serious simulators are clunky and games are tailored to entertainment not serious simulation
- Still a rough path to scaling up (though efforts like CloudSim help)

Pragmatic Suggestions for Moving Forward

[what funding organizations, the robotics community, other vested parties can/should do]

- Autonomous cars: government (e.g. NHTSA) could establish a mid-level procedurally-generated simulator (focused on situations not perception per se) that vehicle autonomy vendors must successfully pass to statistical relevance; this could short-cut all talk of ridiculous "trolley car" scenarios
- Either add serious simulation to game engines or make simulators more game-like:
  - Microsoft AirSim is a welcome effort in this regard but is limited (only quads or autonomous cars, no laser simulators) – expanding this effort to better meet needs of larger robotics community would be valuable, could be led by DoD (to serve, e.g. Synthetic Training Environments Cross-Functional Team, Operation Overmatch, or the Distributed Collaborative Intelligent Systems Technologies CRA)
  - Gazebo would need to be made more interactive (game-like), possibility exists since it will be used as simulator for DARPA Subterranean Challenge
- Could possibly be part of business model for cloud simulation: you get a working complex simulator but it's open enough that you can get under the hood and tweak it to fit your needs – vendor will take on task of collecting and curating the disparate technologies (crowd simulation, dynamic, adversaries, etc.)
  - Ideally dual-mode availability: use it on cloud and pay-as-you-go (academia), can also be deployed to government or corporate cloud for operational or commercial use
  - Emphasis on vendor as integrator not rent-seeker
- Procedural scenario generation could fit well into research portfolios of many government organizations (e.g. NIST, DoD, NSF) and commercial research labs (e.g. Waymo, Apple, Uber)
- Tap into current Deep RL fervor by offering up environment that gets at mid-level robotics concepts (spatial awareness, social awareness, etc.) wrapped up in a nice set of tasks that are as easy to use as Atari

Modeling and Simulation in Robotics Workshop

Attendee Pre-workshop Thoughts

Opportunities

- Easy, accurate simulation of robots is driving robotics research.

- The simulator(+goal) defines an optimization problem:
  - **Classic robotics/control:** System ID, estimation, data smoothing, low-level controller design.
  - **Model predictive control:** Optimize one trajectory. Fast, well-understood. However: requires informative objectives (shaping), state representation is limited to the simulator’s.
  - **Simulation + learning + neural networks:** Sample many trajectories. Scalable (parallelizable), general, robust. Sim-to-real and imitation learning hold promise.

- How to combine Model-based and Data-based?

Problems

- Hardware!
  - The **compliance** required for delicate manipulation is limited by **high-gear-ratio** transmissions.
  - Gears increase **friction**: discontinuous, jerky movement is hard to model.
  - Low-level control **firmware** limits direct access to actuators, adds superfluous complexity.

- Software & standards.
  - Reproducibility and competition are limited by lack of standard **simulators** and **benchmarks**.
  - ROS had a large but limited impact. What is the **next ROS**?

- Organizational. Hardware requires both capital and dedicated human resources

Suggestions

- Standard modeled robots:
  - Standard, printable, open-spec **hardware** (e.g. arm/quadruped).
    - Compliant **joints**, just-strong-enough **actuators**.
    - Direct access to **motors**.
    - High resolution (space+time) **sensors**: joint, IMU, cameras. Soft, sensorized **skin**.
  - Well-**modeled** and well-**identified** with standard simulation software.

- Canonical **tasks** (for above standard hardware).

- Significantly decrease the organizational barriers to entry.

Modeling and Simulation in Robotics Workshop

Attendee Pre-workshop Thoughts

Opportunities

- Simulate worlds, not just robots.
- Generate training data for machine learning.
- Work with systems that are difficult to model analytically
  - Soft robots, soft objects
- Increase development velocity by decoupling data pipelines
  - Independent development/testing of sub-systems based on simulated data.
- VR for Human Robot Interaction and collaborative robotics / Industry 4.0
- Safety testing - finding and testing edge cases
- Closer integration of experimental data and simulation
  - Data driven simulation, world generation.

Challenges

- Generalization
  - Fidelity (at a specific bandwidth and length scale) vs Abstraction (of system details)
  - Different applications lead to different design choices.
- Composing layers of stack in simulation
  - Handling 3rd party middleware: whitebox, greybox, and blackbox scenarios
  - Simulation doesn't easily compose or generalize using the same layered-composition of abstractions architecture typical in robotics. Often better to write separate simulators for each 'level' of abstraction.
- Lack of useful standards
  - For describing robots (URDF/SDF/MJCF)
  - For describing what a simulator does and does not simulate
    - Rigid body dynamics, FEM, task-level world representation…?
    - Low level control (responses accurate at 1Hz or 1kHz?)
    - High level control (e.g. vendor supplied behaviors, perception)
    - System level timing and control flow.
  - For interfacing a control stack with a simulator

Strategy + Next Steps

- Focus on models and worlds
  -
  - Improve model descriptions
    - How should robot models capture and compose different layers of ‘depth’ - micro-scale physics, macro-scale physics, low level actuator control, low level sensors, system level timing/control flow, middleware, high level controllers and tasks?
    - Tools to build, improve, and characterize these models

  - Build tools that make sense for robotics researchers and engineers to build the simulated worlds their robots live in.
    - How do their needs and resources differ from movies and games?

NSF/DOD/NIST Workshop
Modeling and Simulation in Robotics

April 17, 2018 – NIST, Gaithersburg, MD

Dan Negrut
University of Wisconsin-Madison

Acknowledgements

- University of Washington workshop co-organizers
  - Dieter Fox
  - Arun Byravan
  - Franzi Meier

- The group who instigated us
  - Reid Simmons – NSF
  - David Han – ARL
  - Jordan Berg – NSF

Acknowledgements

- We want to acknowledge all of you, who came from all over
- Thank you for taking the time and caring about this

Acknowledgements

- Our NIST friends for hosting us here
  - Elena Messina
  - Gladys Arrisueno
  - Karen Startsman
  - Crissy Robinson
  - Mary Lou Norris
  - Sandy Ann Gray
  - Dean Winning

This Will be One Long Day

- Three breakout sessions and three plenary sessions

- Some longer coffee breaks
  - Good for networking and saying hi to old friends, making new friends

- Short lunch, catered, we’ll eat here

Goal

- Fact finding meeting. Ultimate goal is to help robotics deliver on its potential
  - Breakout 1: discuss ways in which modeling & simulation does/should/can help robotics
    [a time to dream]
  - Breakout 2: discuss what’s preventing modeling & simulation to do more for robotics at this time
    [reality check]
  - Breakout 3: Anything that should/could be done so that modeling & simulation improves robotics?
    [actionable items]

What a Breakout Is

- Team of 8-9 people gets locked up in a room for 25 mins to do this:
  - Select a scribe to compile three slides (template provided)
  - Decide who will present the Team's slides in the plenary discussion
  - Set out to generate diverse/original/out-there ideas to populate the three slides
  - Stop promptly after 25 mins and re-join everybody else in the plenary room

What Team Do You Play On?

- Consult your cheat sheet…
  - You’ll find out what Team you are on
  - You’ll find out which room your Team meets in

What Happens After each Breakout

- Each Team presents its slides (has 5 mins to do so) in plenary session

- 5 Mins × 6 Teams = 30 Mins

- After 30 mins we open the floor for general discussions, of which we'll have 30 more mins
  - Ideas that were not captured in any slides
  - "Open floor discussion" runs for 30 mins

Things That Might Sink This Workshop

- Breakout Session: Debating one topic for too long
  - If you don't reach a consensus after a three minutes or so, move the topic/idea to Slide 2
  - The Teams are supposed to churn out ideas
- Plenary Session: Trying to settle open issues in arguments that pitch one person/group against another person/group
- Keeping quiet
- Running behind and not sticking to the schedule

Outcomes, Today's Meeting
[1/2]

- A report will be generated
  - Relatively easy, we will take minutes and also have your slides

- Report forwarded to task group that meets monthly in DC
  - They'll look at the information we generate and decide on a course of action

Outcomes, Today's Meeting
[2/2]

- We should decide whether a focused journal article compiling our thoughts on the topic of modeling and simulation in robotics is in order
  - Would be written in the upcoming months
  - Perhaps it would be a valuable doc to have in the community

Putting Things in Perspective…

- Contribute original ideas, make your voice heard

- Engage in constructive discussions

- Stay on time, stay focused

Great to have you here. Thank you for being part of this.

ROBOTICS@NVIDIA WORKSHOP

04/23 – 04/24 2018

NVIDIA

AGENDA: DAY 1

12:00 – 1:00	**LUNCH**
1:00 - 1:45	**WELCOME AND ROBOTICS RESEARCH PERSPECTIVE** – DIETER FOX
1:45 - 3:15	**ROBOTICS INDUSTRY AND ISAAC**
	INDUSTRY – MURALI GOPALAKRISHNA [30 MIN]
	ISAAC SDK – CLAIRE DELAUNAY [30 MIN]
	ISAAC SIMULATION – LIILA TORRALBI [30 MIN]
3:15 - 3:45	**BREAK**
3:45 – 4:45	**BREAKOUT 1** – 3 PARALLEL SESSIONS
	ROBOTICS: *HOW CAN WE MAXIMIZE NVIDIA IMPACT?* – DAVID W. / NATHAN R.
	SIMULATION: *WHAT ARE THE NECESSARY CAPABILITIES AND ROADBLOCKS?* – LIILA T. / DUNCAN M.
	DEEP LEARNING: *WHERE CAN IT HELP AND WHAT DO WE NEED TO ENABLE IT?* – STAN B. / ANKUR H.
4:45 – 5:30	**SPEED TALKS** (9 X 5 MINUTES)

Agenda: Day 2

8:00 – 8:45	**Breakfast**
8:45 - 9:00	**Welcome** – Dieter Fox
9:00 - 10:00	**Lessons from Automotive**
	Strategy – Michael Cox
	Infrastructure – Clement Farabet
	Hardware-in-the-Loop – David Auld
10:00 - 10:15	**Break**
10:15 – 10:45	**Breakout 1 Reports and Discussion**
10:45 – 11:15	**Speed Talks** (6 x 5 minutes)
11:15 – 12:00	**Breakout 2**
	**3 *x* Robotics / simulation strategy** – Claire D. / Dieter F. / Murali G.
12:00– 1:00	**Lunch**
1:00 – 2:00	**Breakout Reports and strategic Direction Discussion**

IN MEMORY OF JAN ISSAC

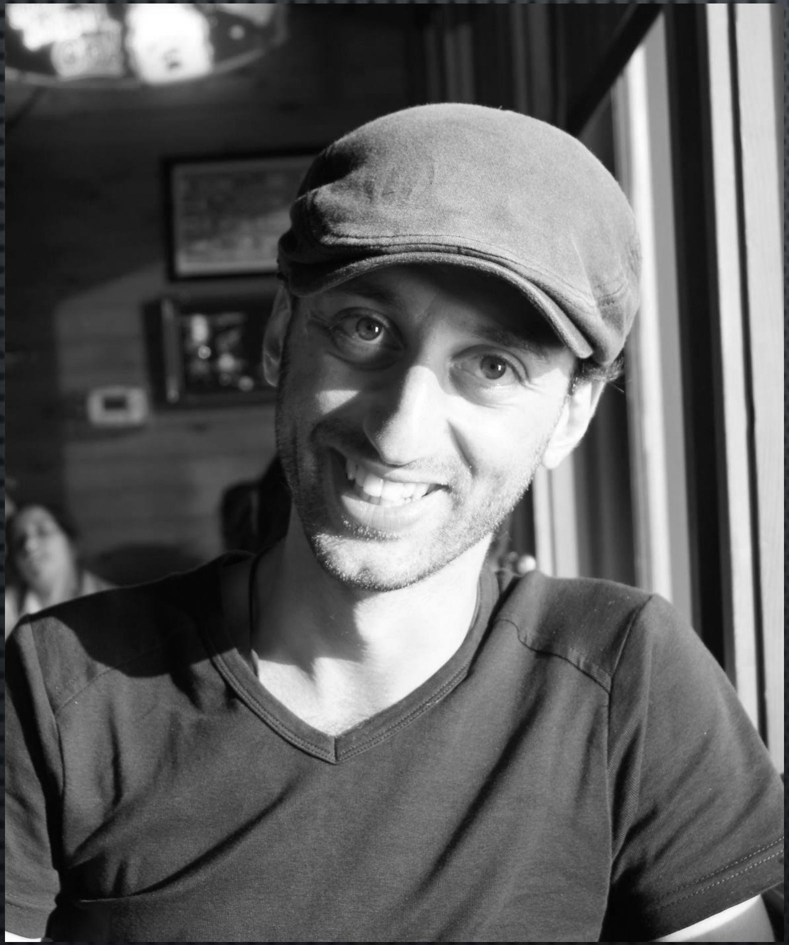

Jan was a founding member of the NVIDIA Robotics Lab Seattle.

We will remember him as a friend and an incredibly generous, funny, and hardworking member of our community.

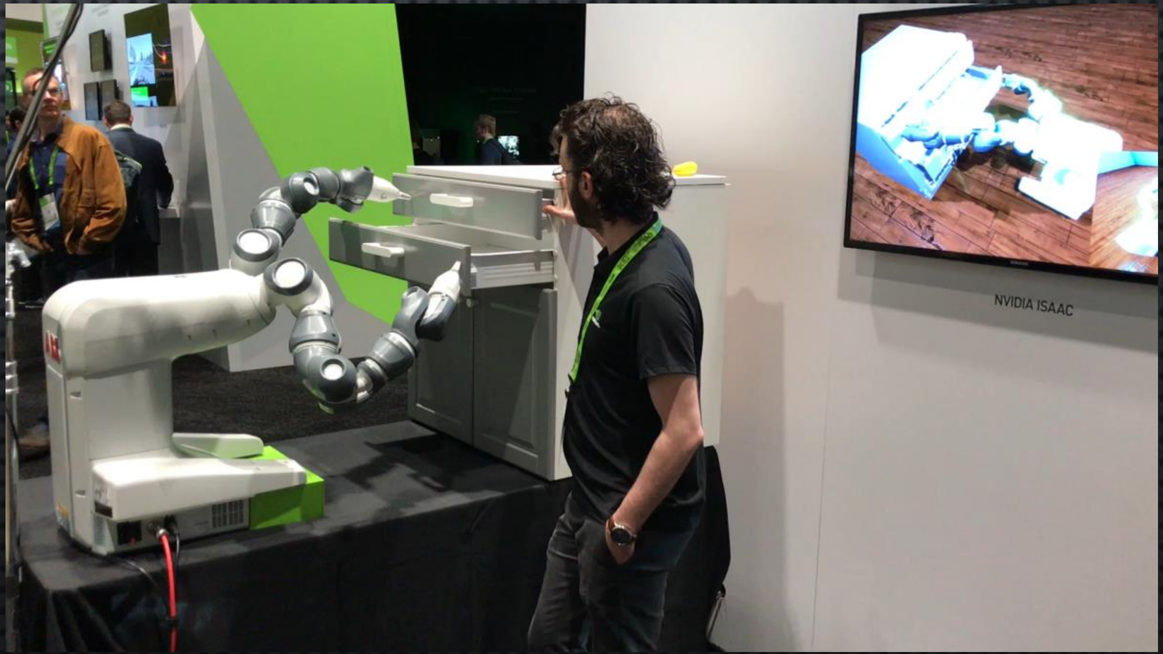

Stan Birchfield Jonathan Trembla

TODAY'S HOT ROBOTICS APPLICATION AREAS:
FULFILLMENT, INVENTORY, DELIVERY, DRIVING

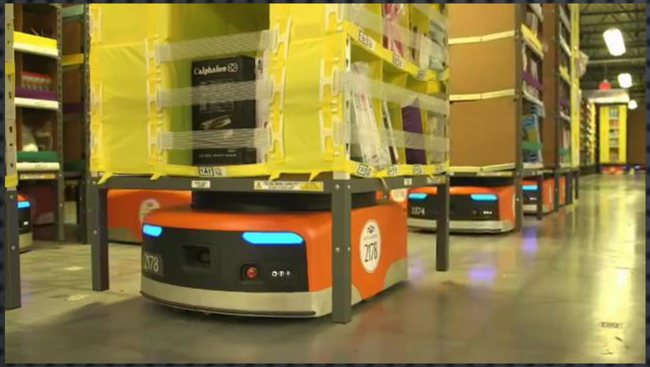

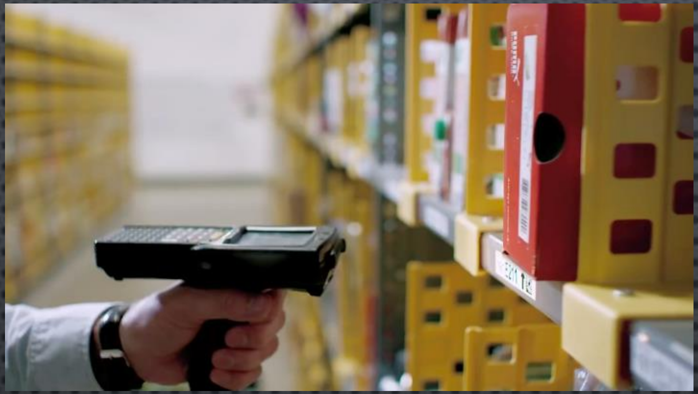

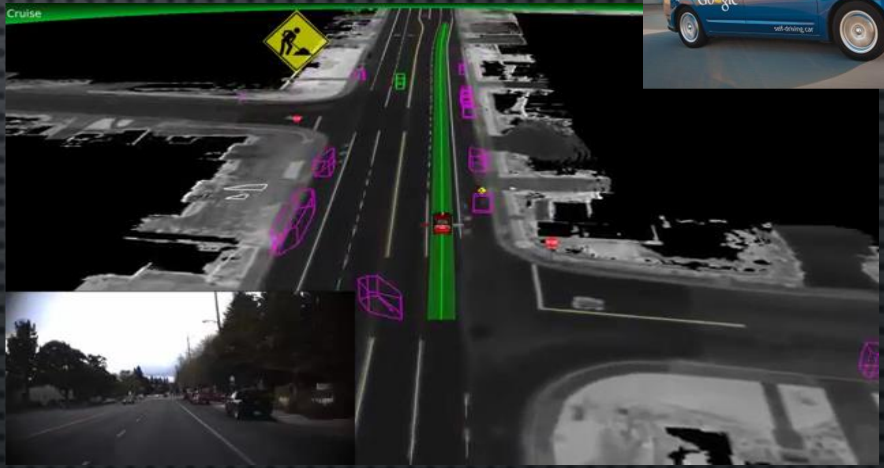

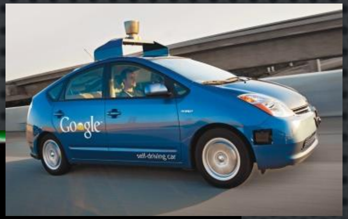

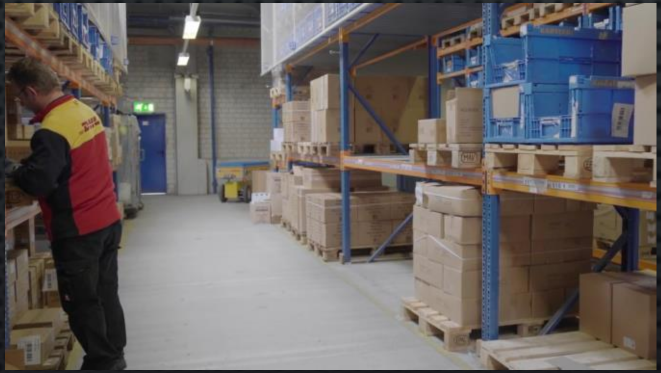

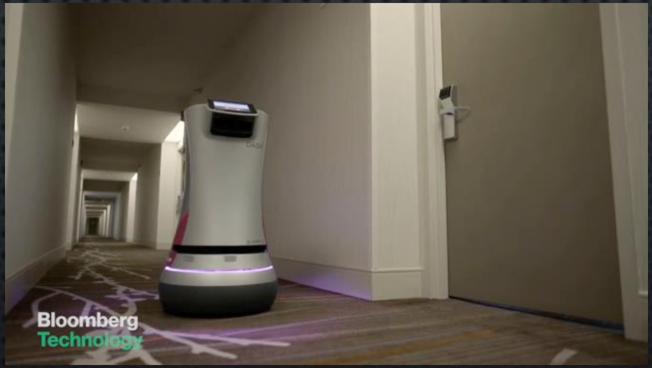

TODAY'S HOT ROBOTICS APPLICATION AREAS: FULFILLMENT, INVENTORY, DELIVERY, DRIVING

Upcoming applications of robots focus on getting from A to B without crashing into something

The next generation of robots will be about physical interaction with the environment and people

INDUSTRIAL MANIPULATION TODAY

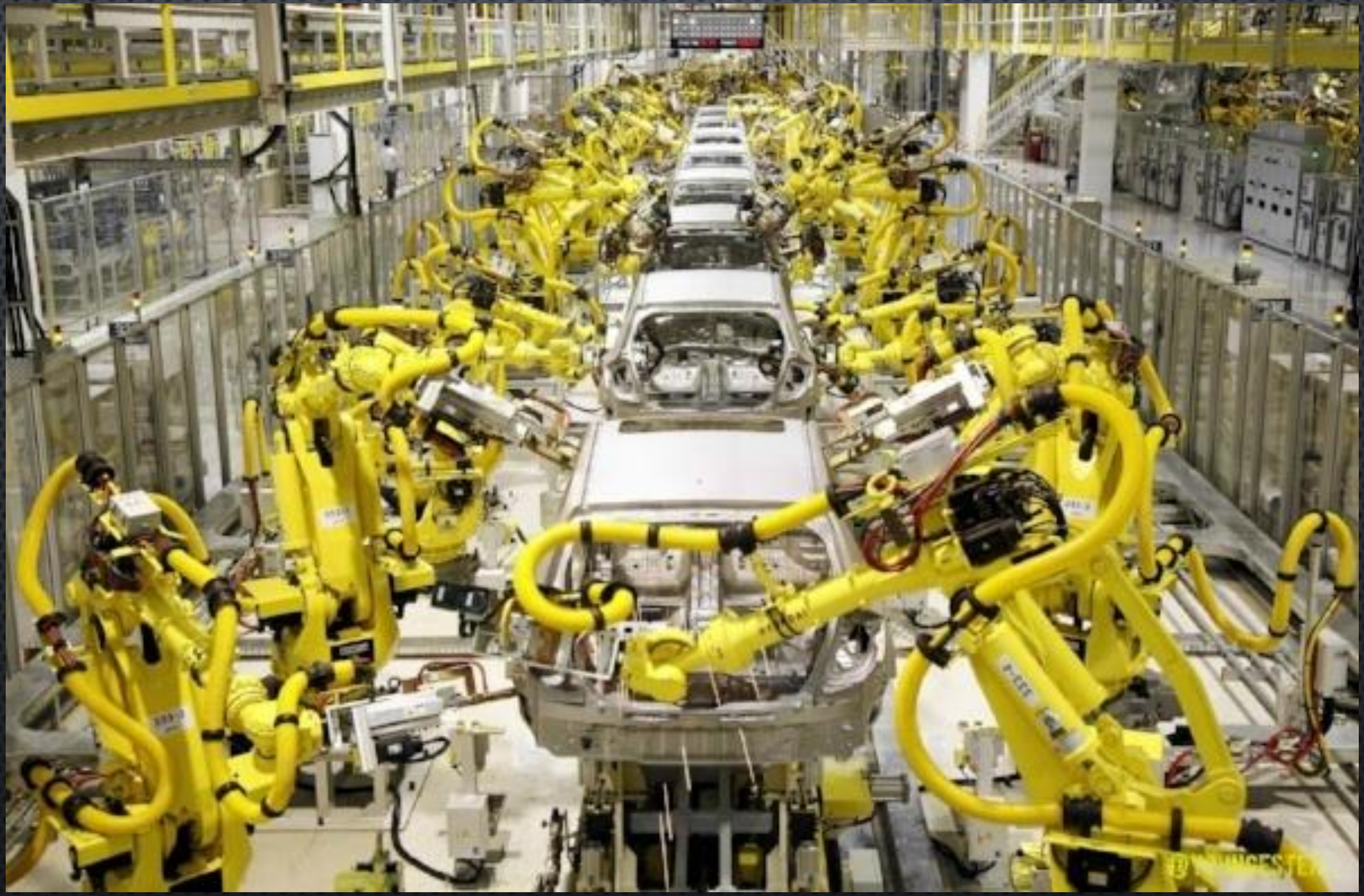

INTERACTIVE MANIPULATION

Robots that interact with people in a natural way, perform complex tasks with people, learn necessary objects, attributes, skills, tasks from people.

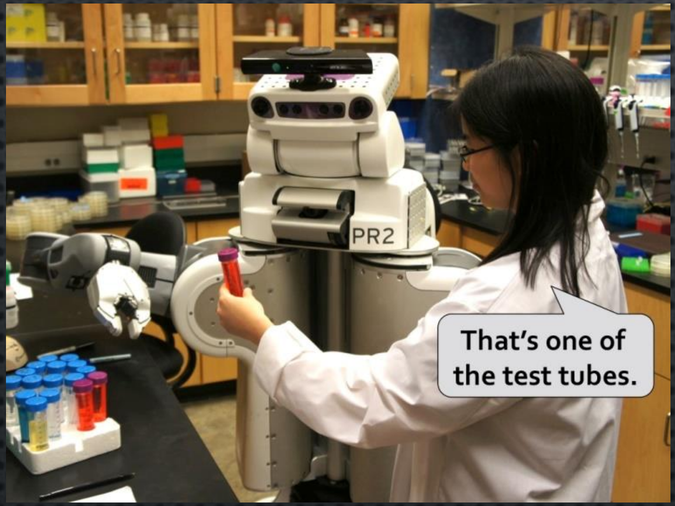

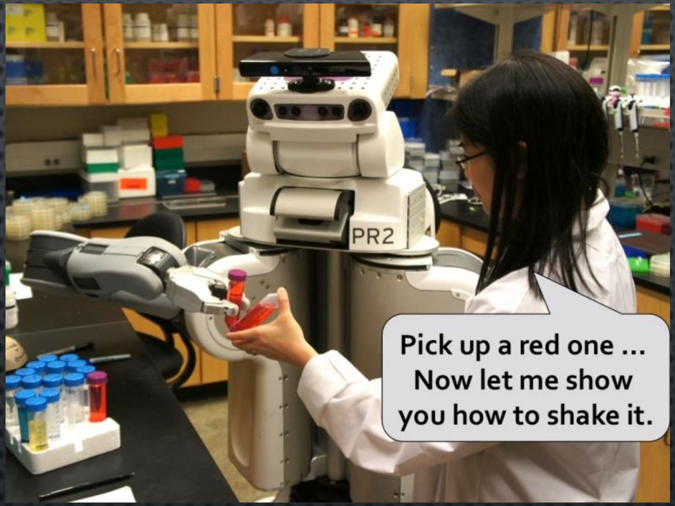

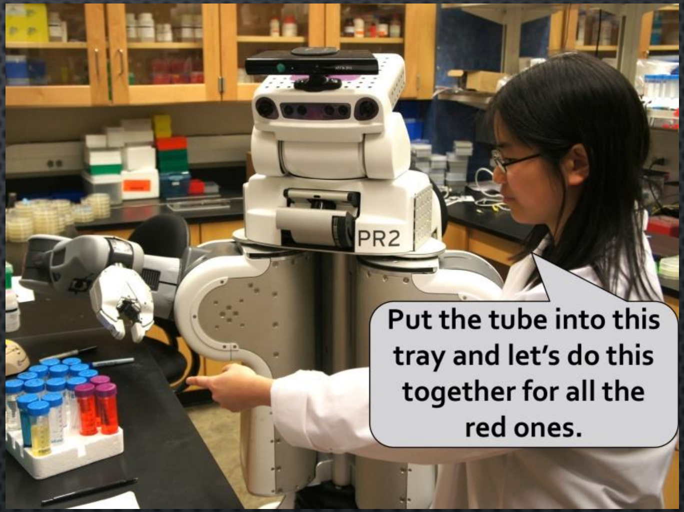

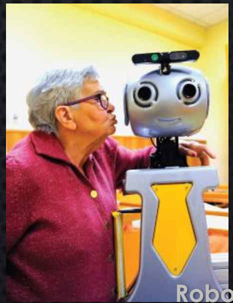

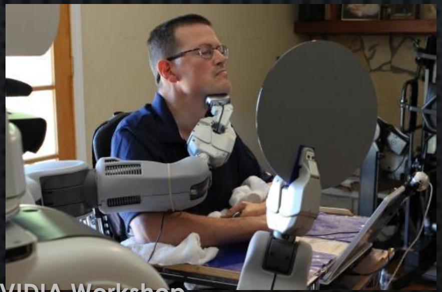

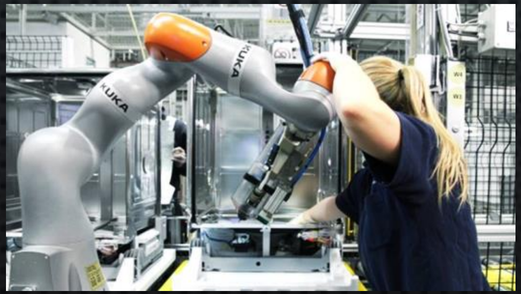

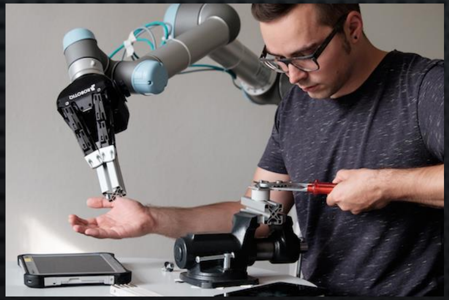

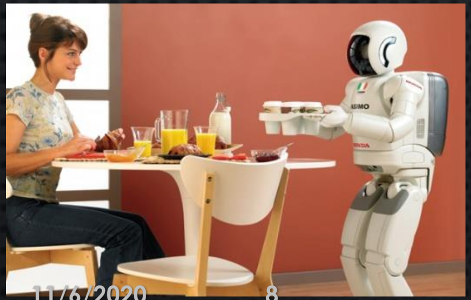

Outline

- Simulation for Development and Testing
- Simulation for Planning and Control
- Simulation for training and learning
- Kitchen as driving scenario for research
- Conclusion

Prevalent use cases of simulation in robotics

- Simulation for Development and Testing
  - Run robot control system in simulated environment
  - Realistic scenarios and sensor / dynamics models
- Simulation for Planning and Control
  - Run robot in real world, use simulator as world model
  - Incorporate perception to match state of simulator with real world
- Simulation for training and learning
  - Train robot perception and control in simulated environment
  - Less focus on system integration, specialized scenarios

DEVELOPING AND TESTING COMPLETE CONTROL SYSTEMS

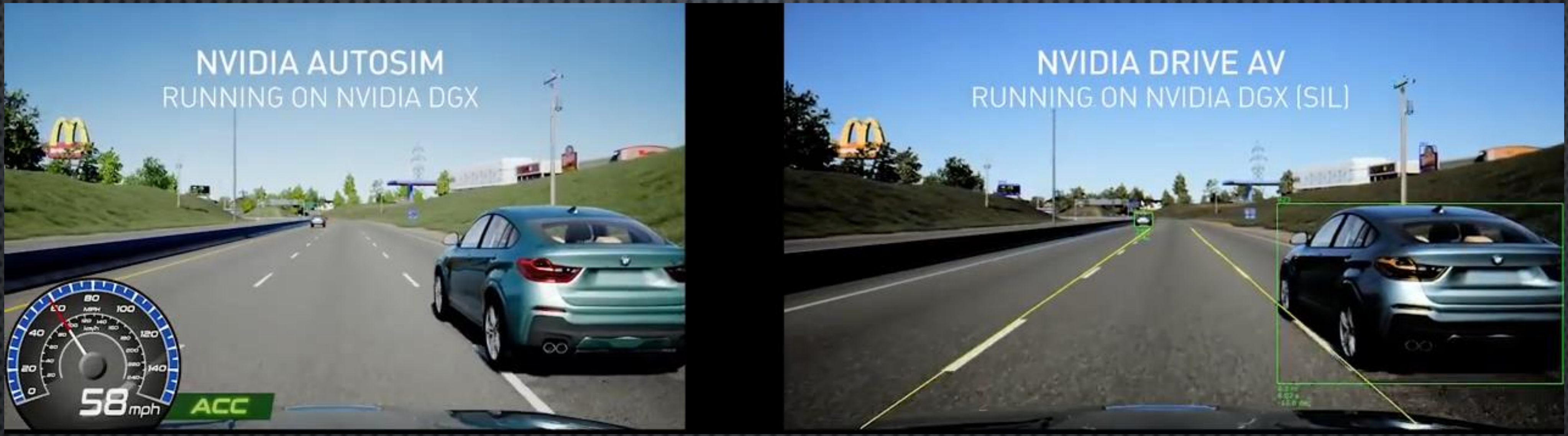

NVIDIA AUTOSIM

- Entire system sim
- Photorealistic rendering, Lidar, Radar, …
- Low-level controls
- Realistic timing
- Other agents
- Limited contact modeling

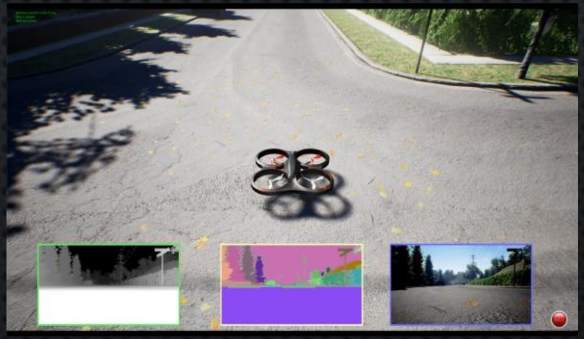

MSR AIRSIM

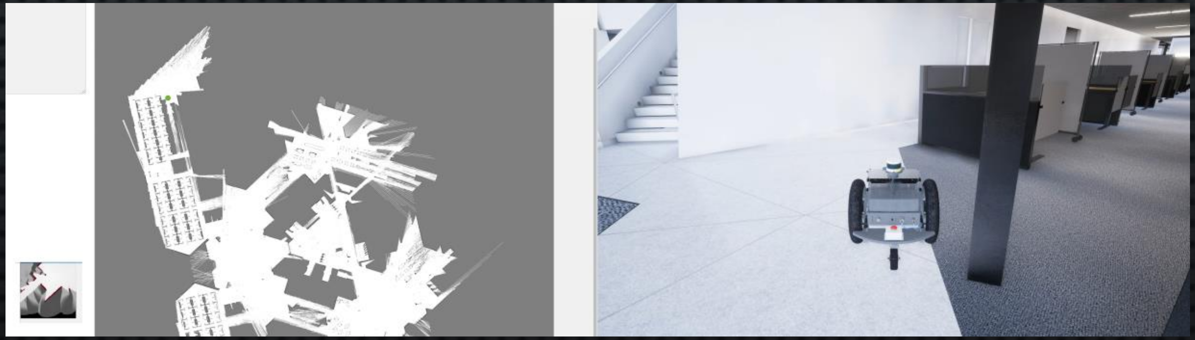

NVIDIA ISAAC

Outline

- Simulation for Development and Testing
- Simulation for Planning and Control
- Simulation for training and learning
- Kitchen as driving scenario for research
- Conclusion

SIMULATION FOR CONTROL

Contact-invariant optimization for hand manipulation

Mordatch, Popovic and Todorov
SCA 2012

[Mordatch-Popovic-Todorov] using MuJoCo

SIMULATION FOR TASK AND MOTION PLANNING

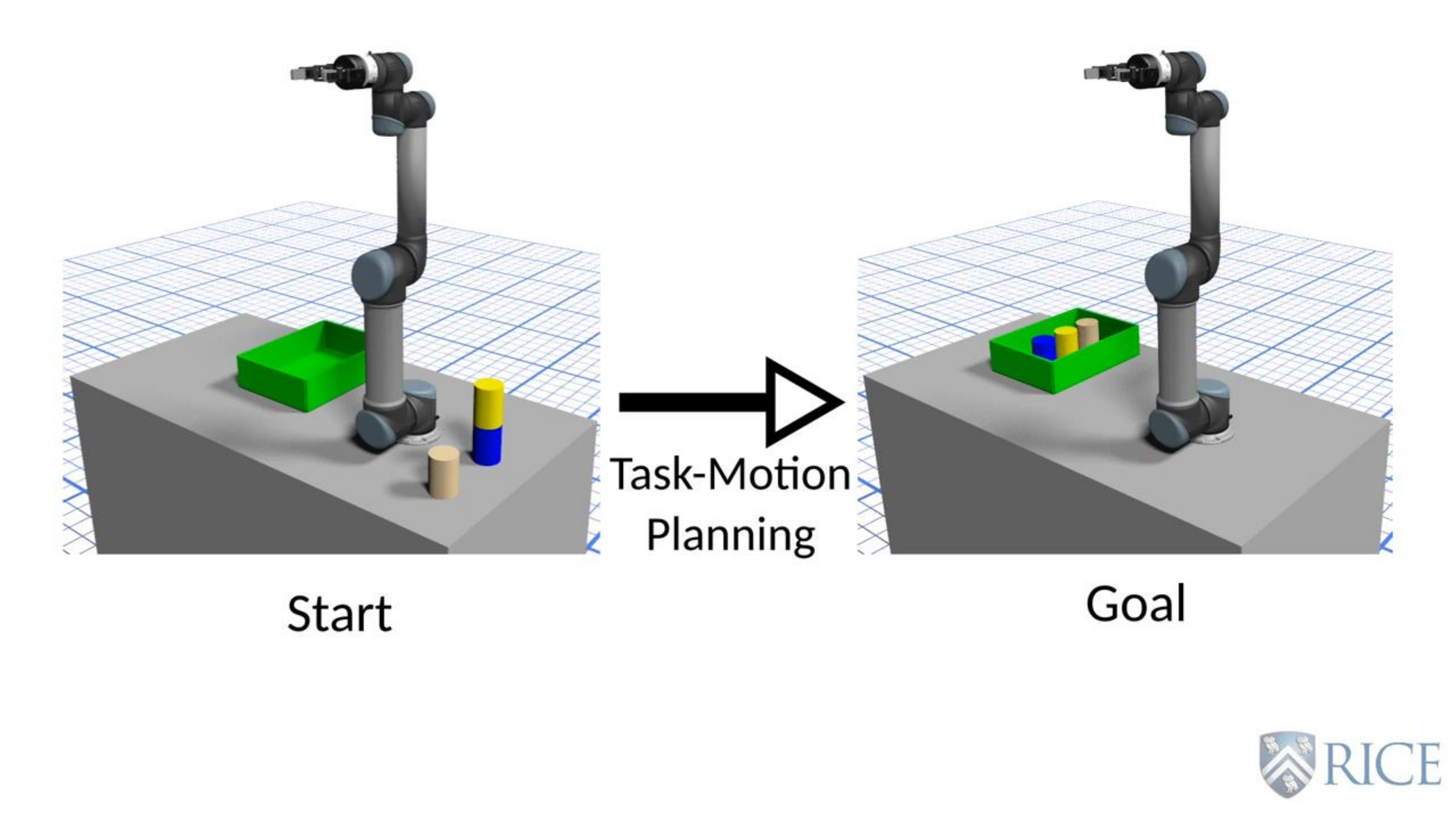

[Dantam-Chaudhuri-Kavraki]

[Schmidt-Newcombe-Fox: RSS-14, ARJ-15]

DART: CLOSING THE LOOP BETWEEN SIMULATION AND REALITY

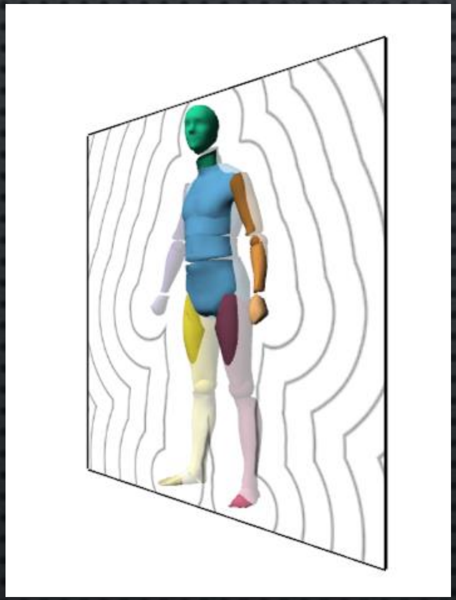

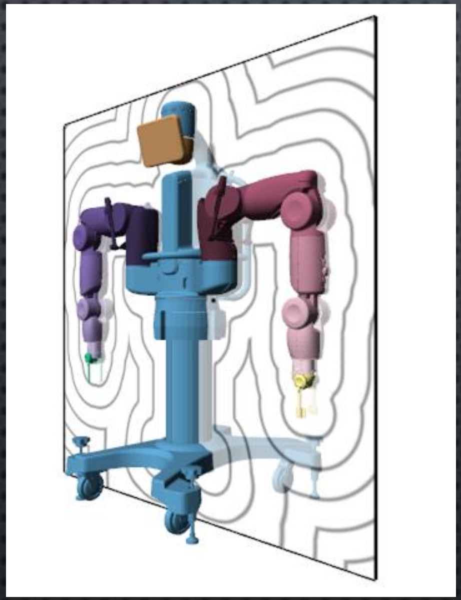

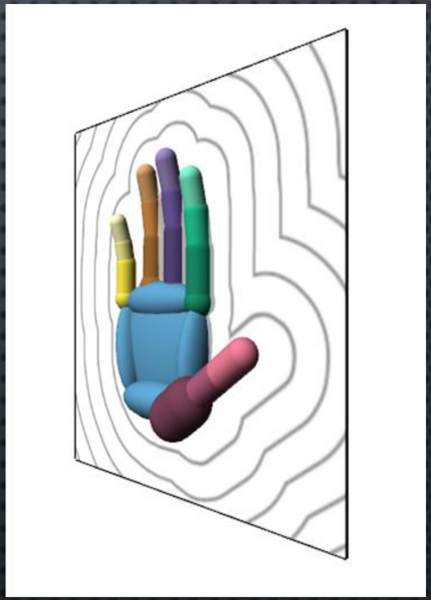

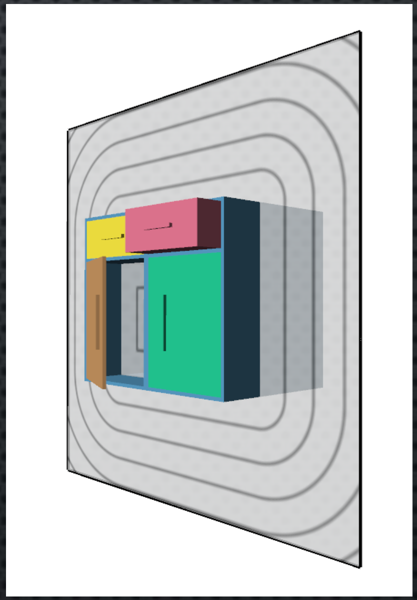

TRACKING A MANIPULATOR AND OBJECT:

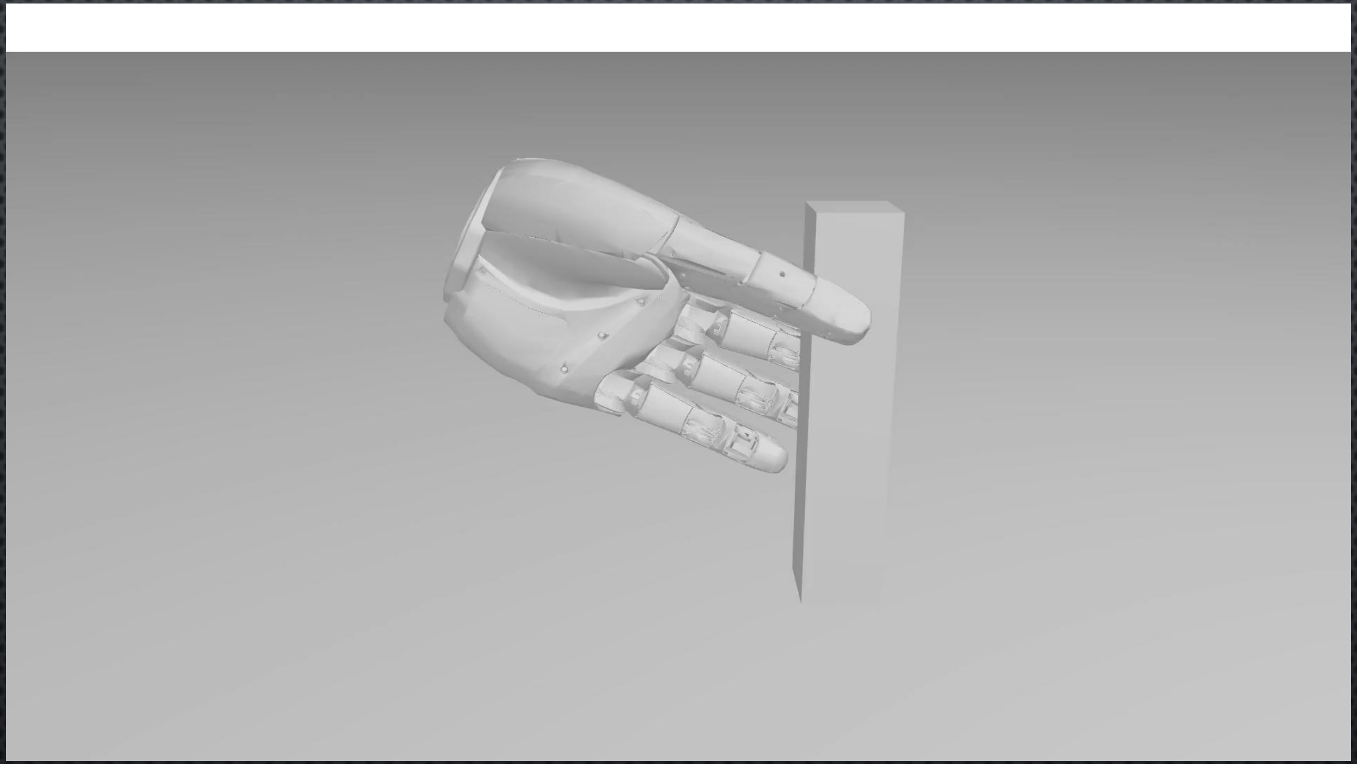

$$L_{match}(\theta) = \sum_{u} \left(SDF(u, \theta)\right)^2$$

TRACKING A MANIPULATOR AND OBJECT:

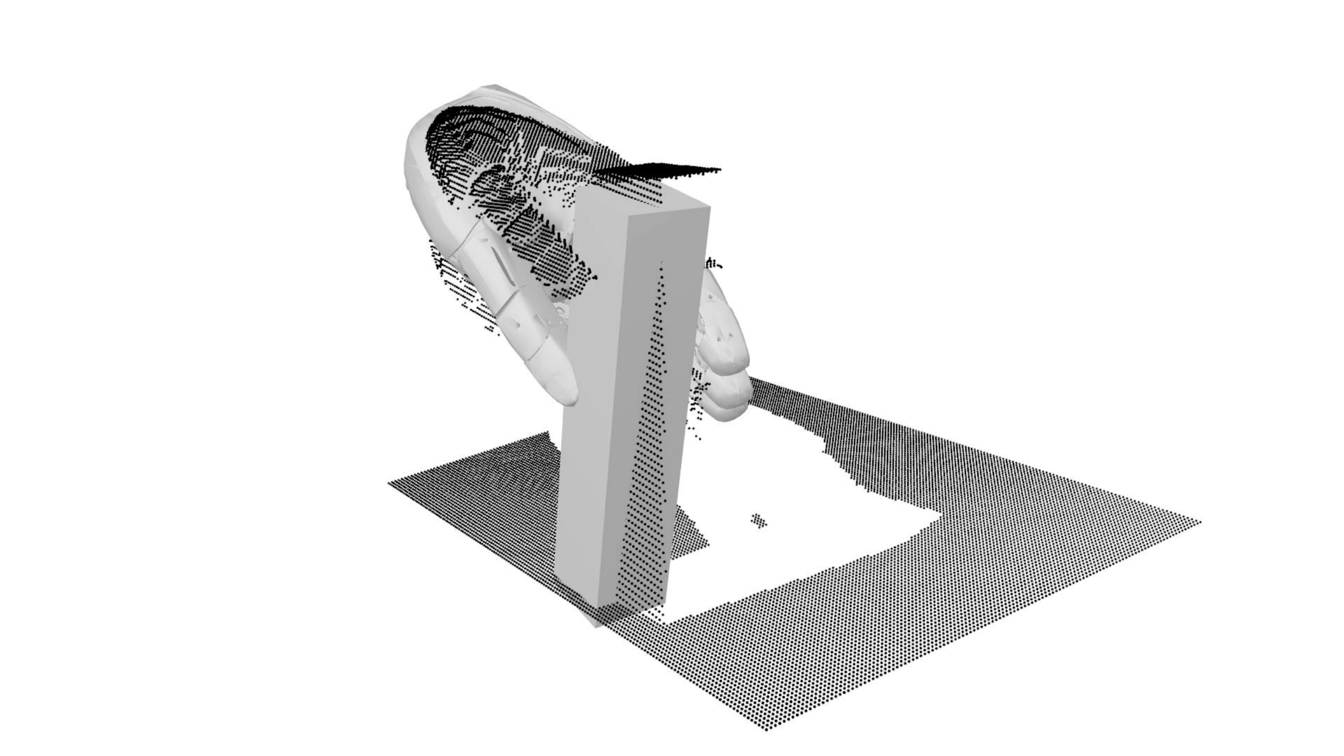

$$L_{match}(\theta) = \sum_{u} \left(SDF(u, \theta)\right)^2$$

TRACKING A MANIPULATOR AND OBJECT: TOUCH FEEDBACK

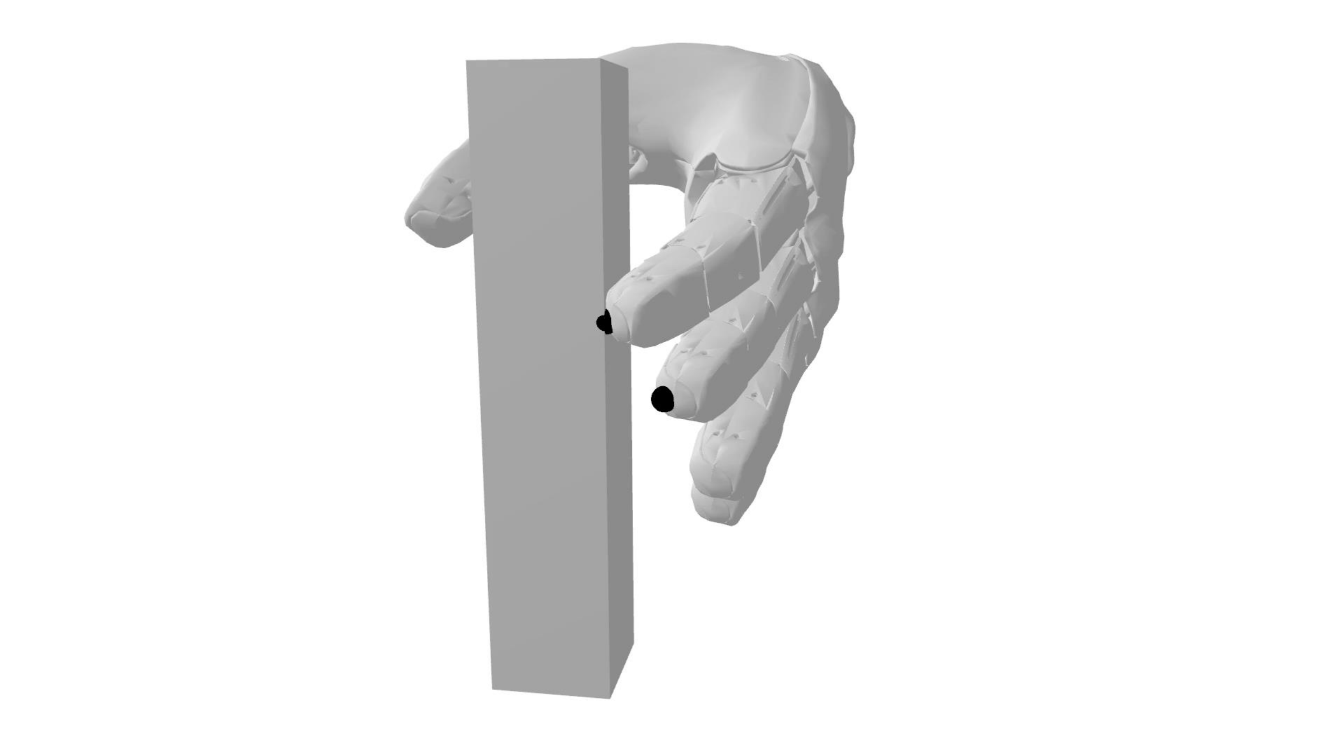

$$L(\theta) = L_{match}(\theta) + L_{cont}(\theta)$$

TRACKING A MANIPULATOR AND OBJECT: PHYSICAL CONSISTENCY

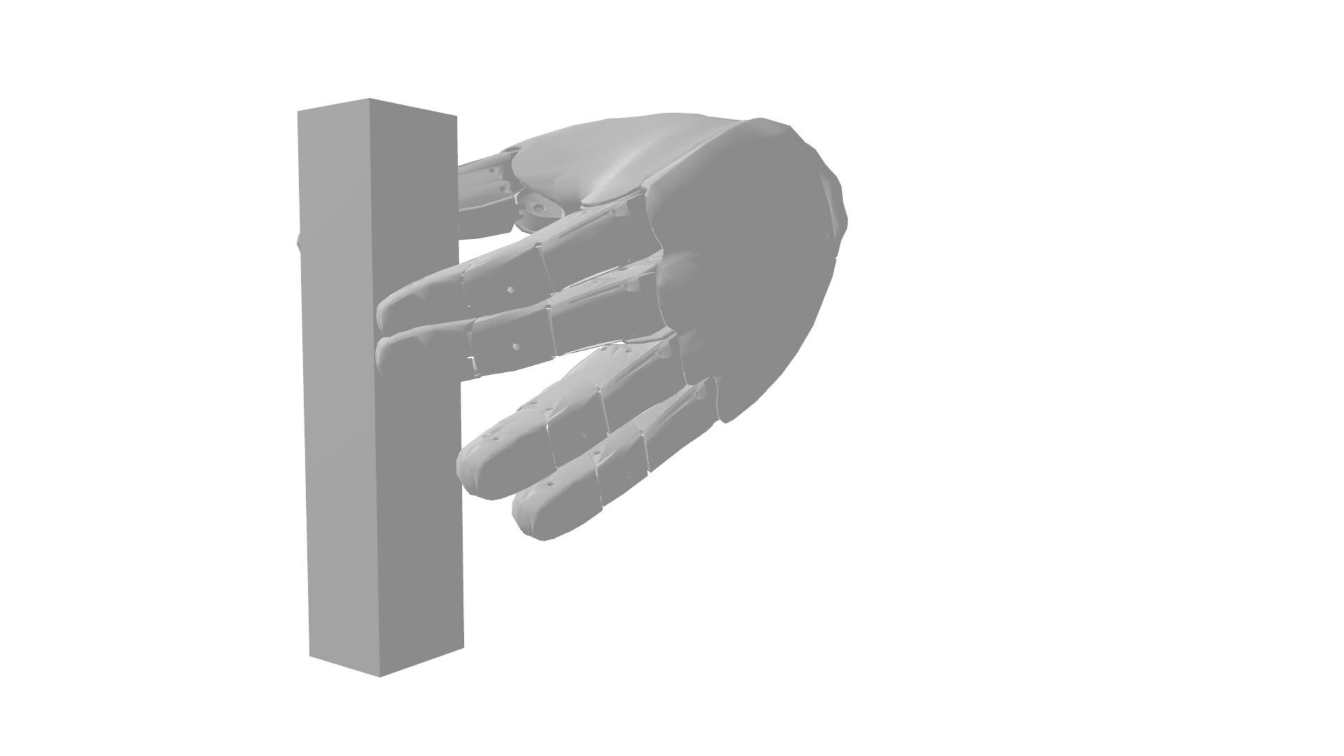

$$L(\theta) = L_{match}(\theta) + L_{cont}(\theta) + L_{inter}(\theta)$$

FINE-GRAINED MANIPULATION

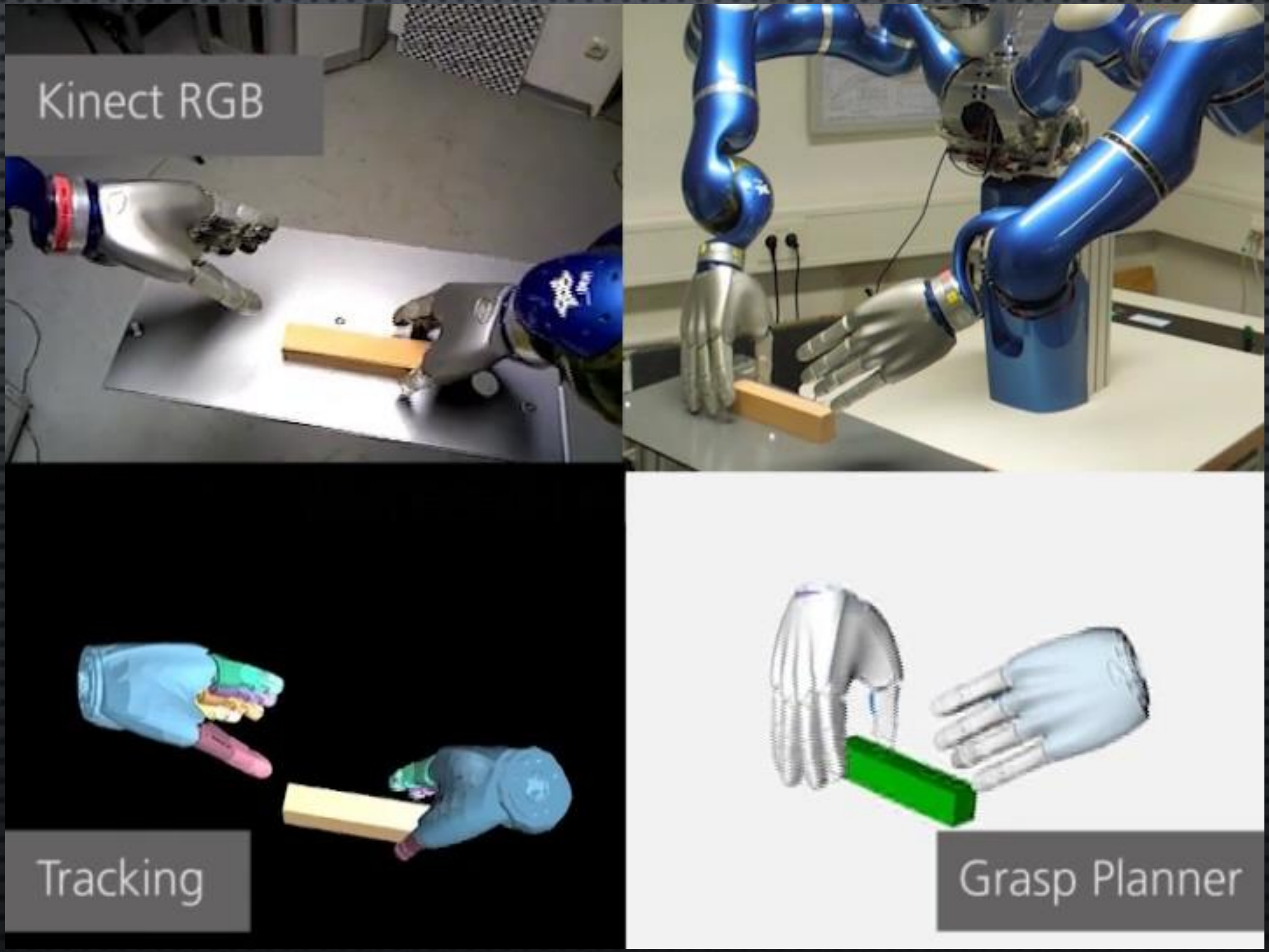

RIEMANNIAN MOTION POLICIES W/ DART

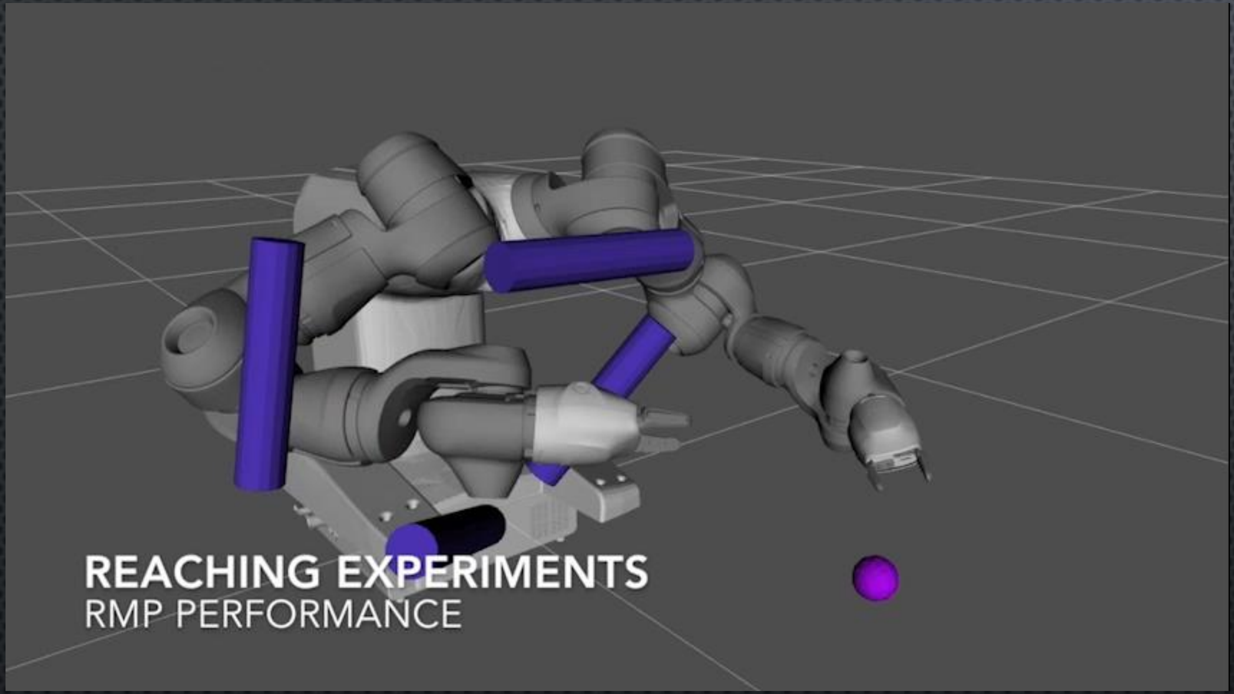

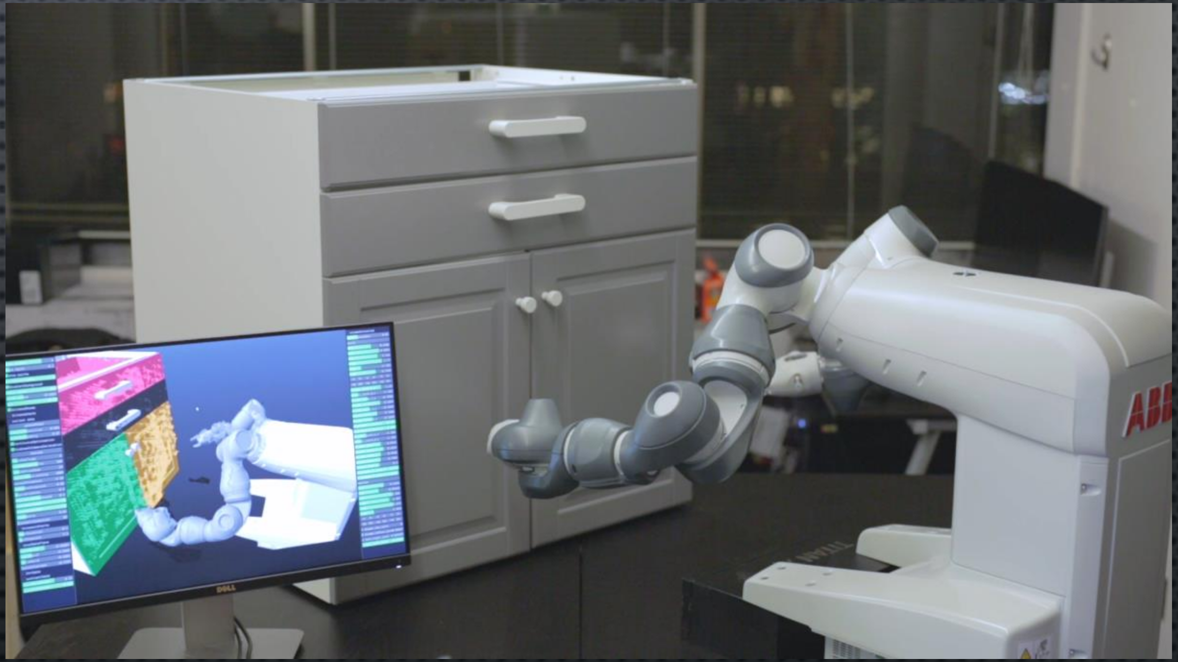

[Ratliff-Issac-Kappler-Birchfield-Fox]

[Schenck-Fox: ISER-16, RSS-17]

REASONING ABOUT LIQUIDS

- Assume we can track 3D models and know initial water amount
- Run Smoothed Particle Hydrodynamics simulation

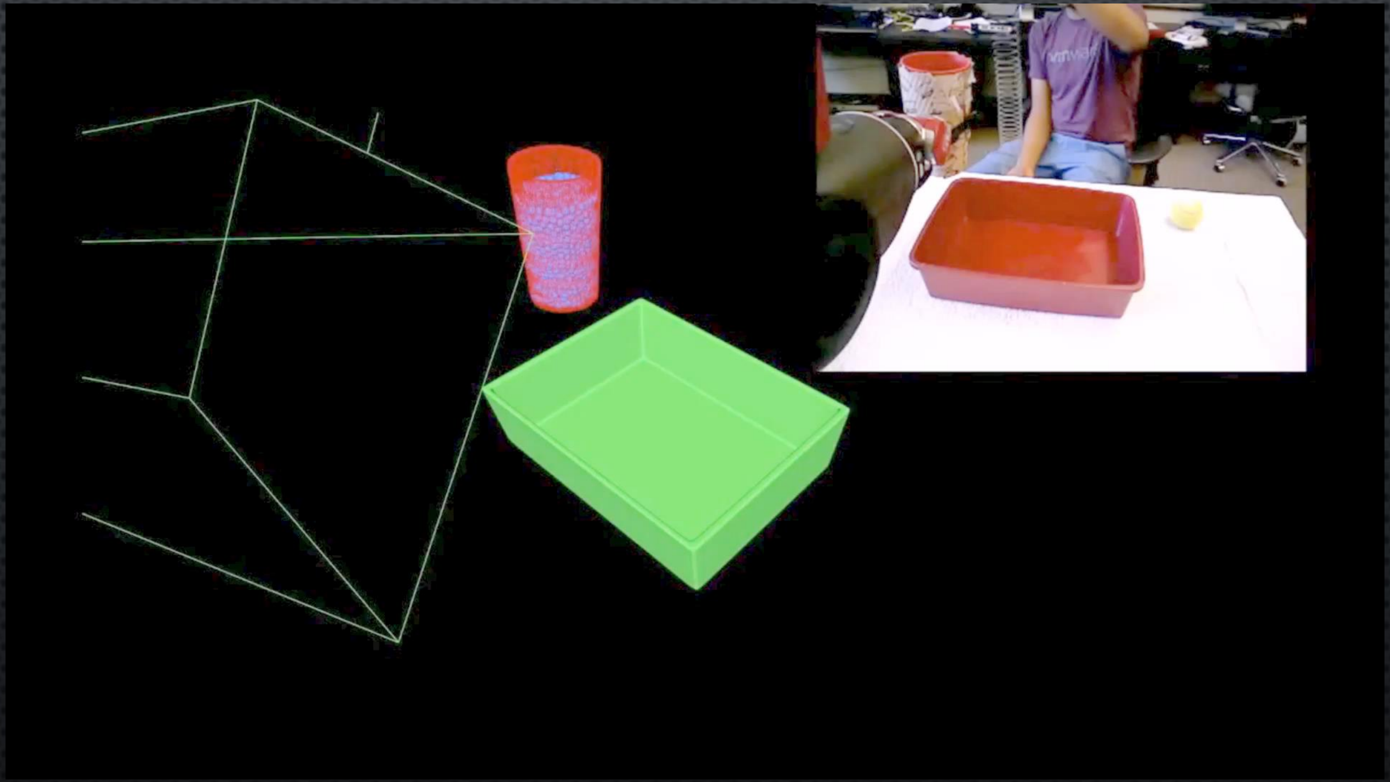

REASONING 1: UNKNOWN INITIAL AMOUNT

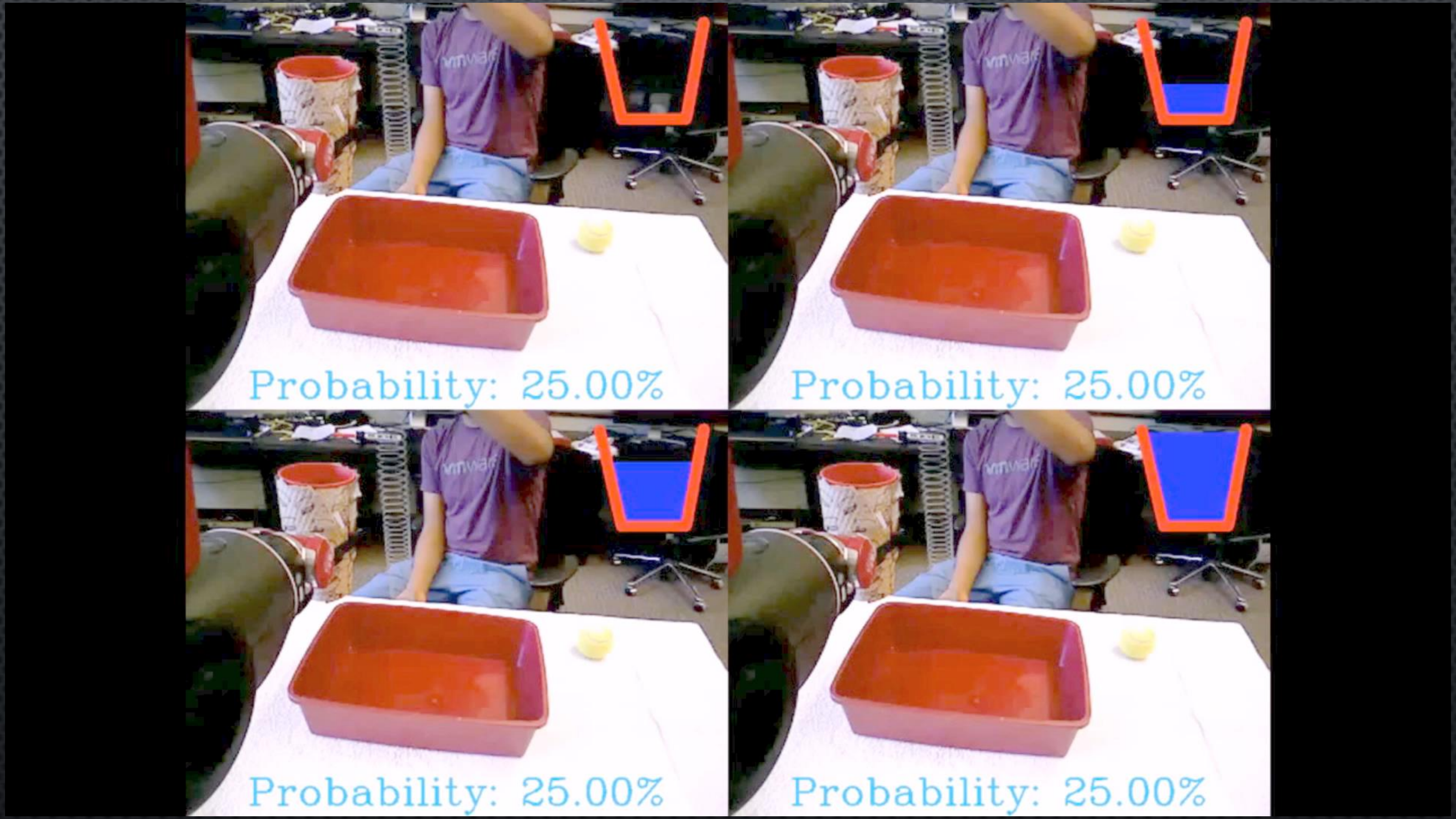

Likelihood by comparing detected water pixels with predicted pixels

REASONING 2: UNKNOWN BLOCKAGE

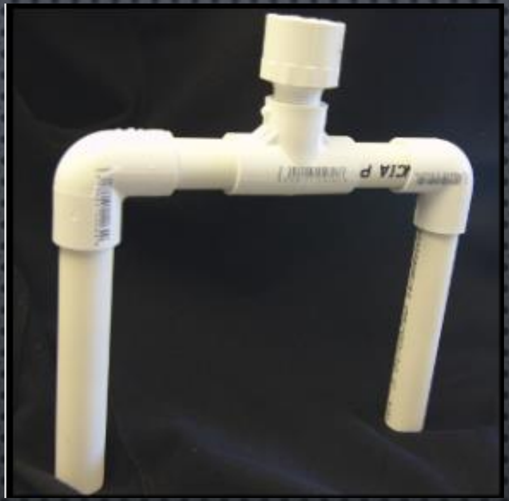

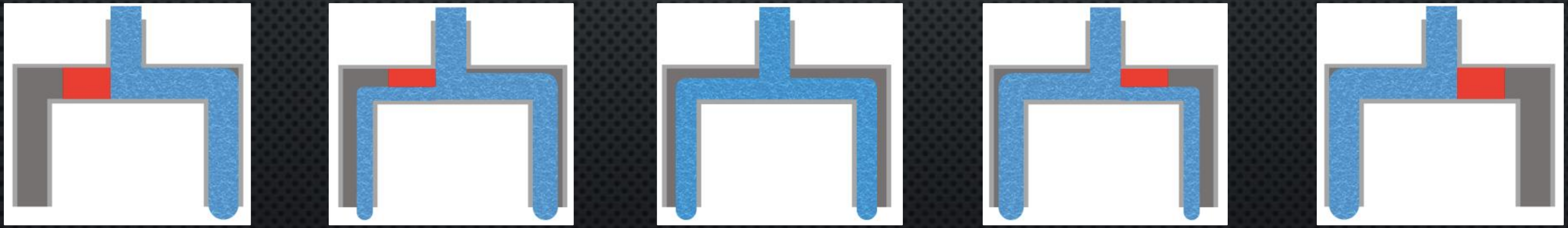

REASONING 2: UNKNOWN BLOCKAGE

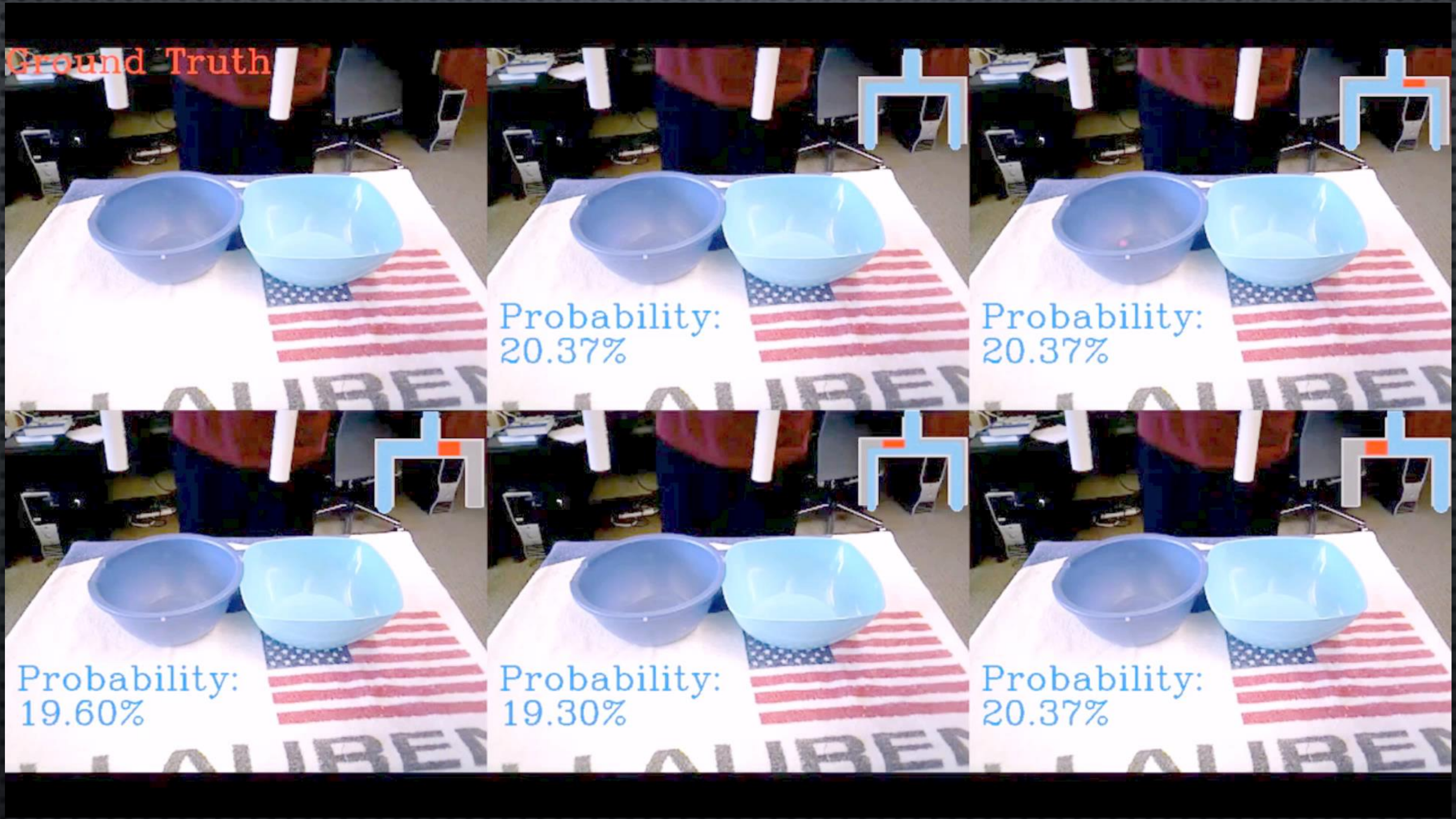

Outline

- Simulation for Development and Testing
- Simulation for Planning and Control
- Simulation for training and learning
- Kitchen as driving scenario for research
- Conclusion

TRAINING 6D POSE DETECTION: YCB-VIDEOS DATA

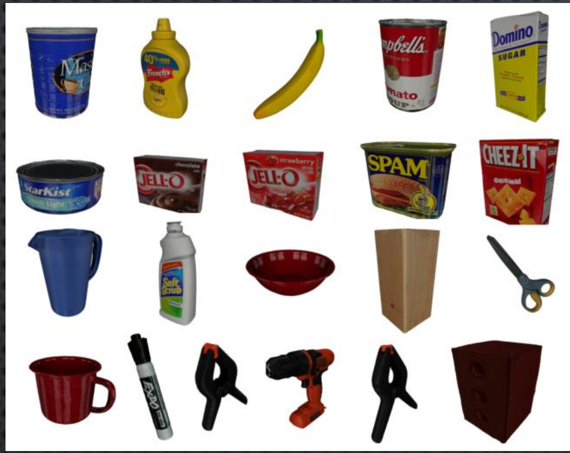

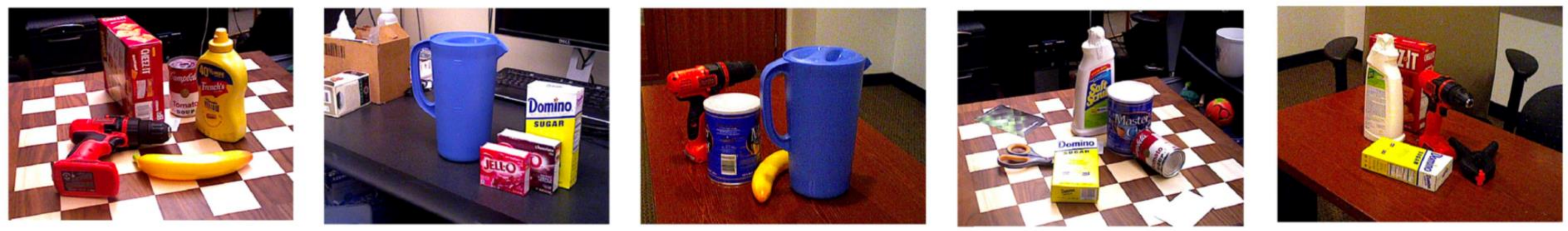

Example scenes

Number of Objects	21
Total Number of Videos	92
Held-out Videos	12
Min Object Count	3
Max Object Count	9
Mean Object Count	4.47
Number of Frames	133,827
Resolution	640 x 480

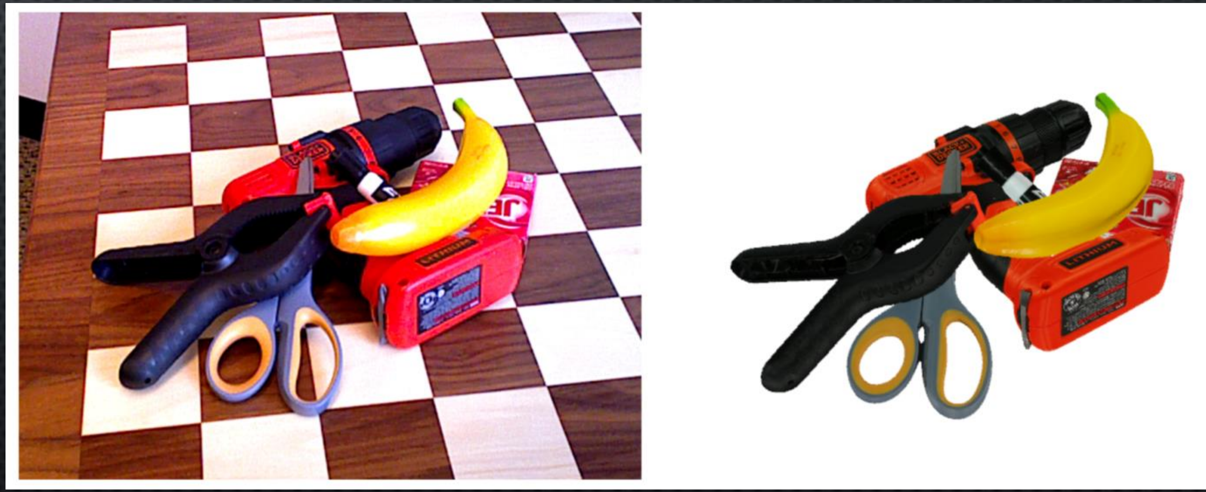

Real Rendered objects

TRAINING 6D POSE DETECTION VIA RANDOMIZATION

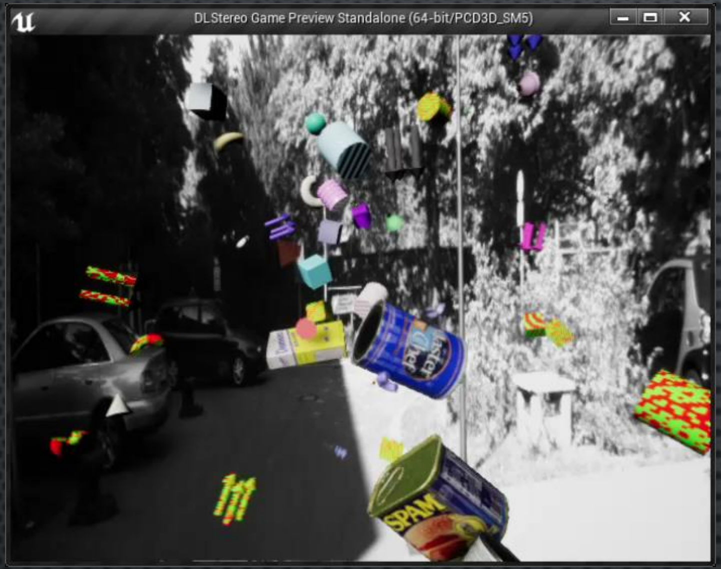

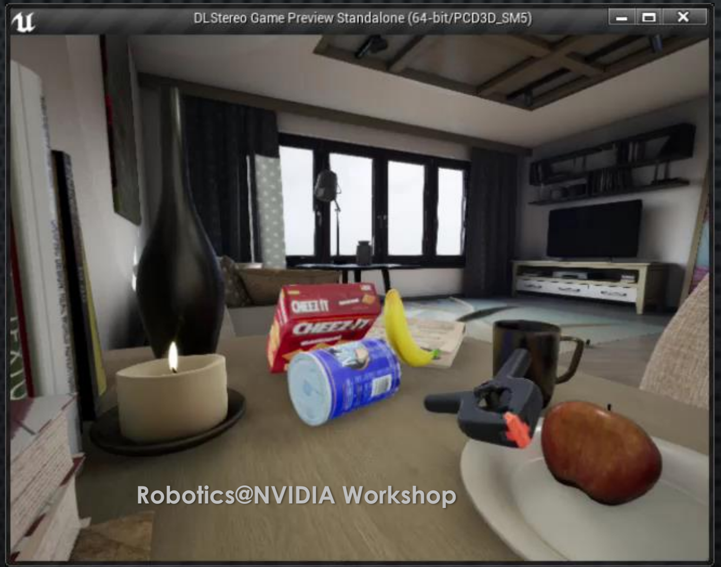

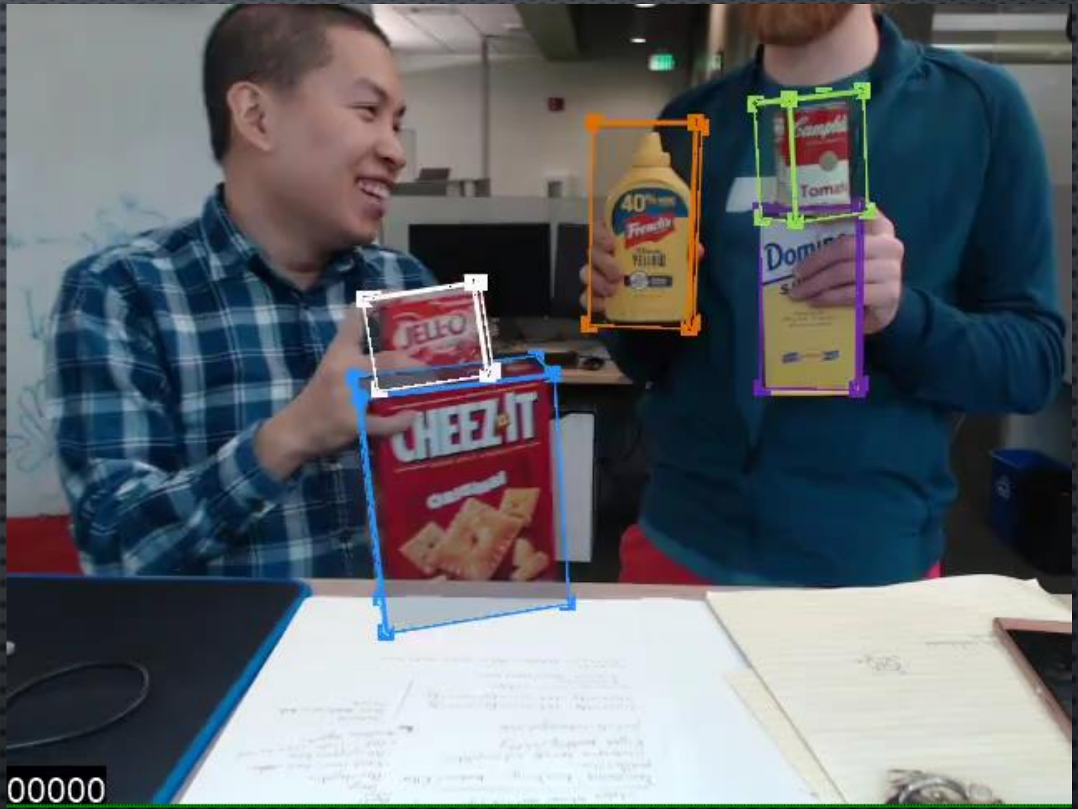

- Randomize lighting, context, location, reflectance, …
- Realistic content creation is hard problem

RL FOR LOCOMOTION, NAVIGATION, AND MANIPULATION

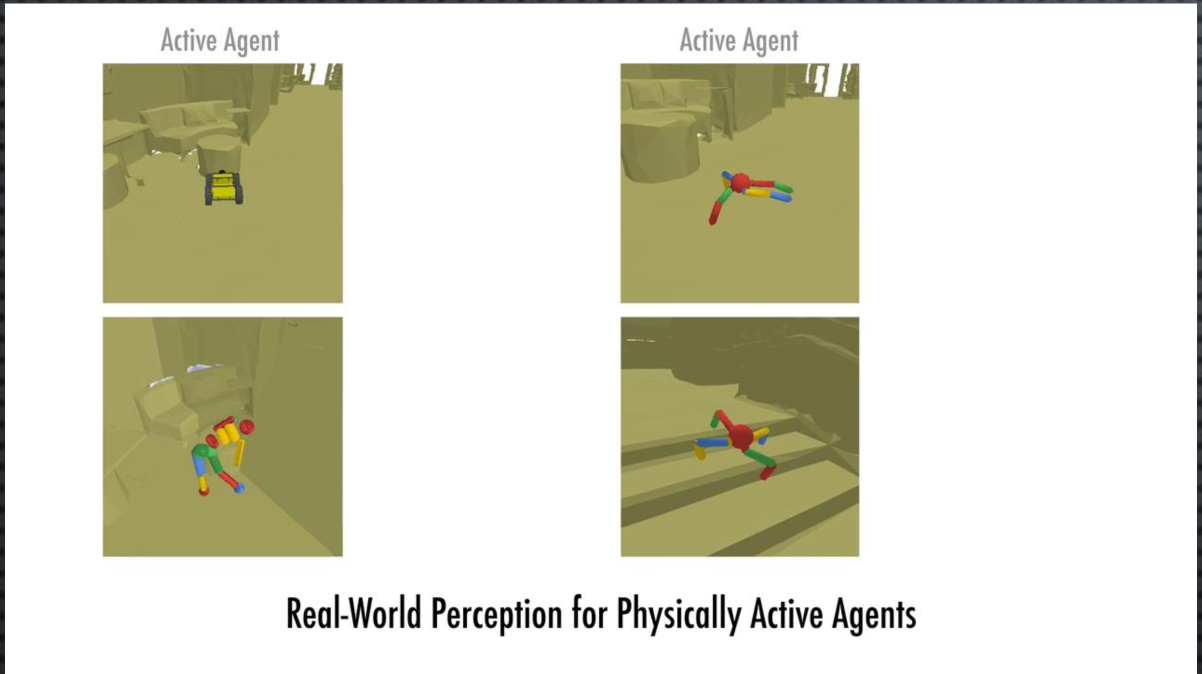

[Gibson: Zamir-Xia-He-Sax-Malik-Savarese: Stanford/Berkeley]

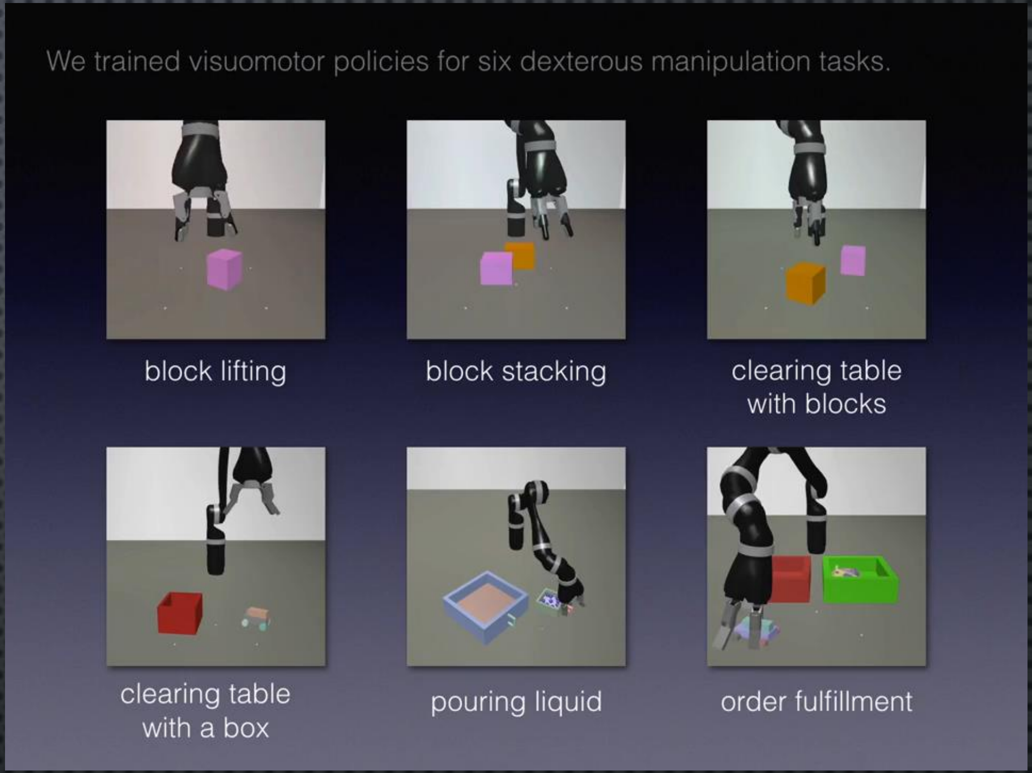

[Zhu-etal: Stanford/DeepMind]

- Existing simulation environments are still very limited
- Stand-alone systems, lacking photo-realism, touch, contact, …

VR FOR ROBOT TRAINING

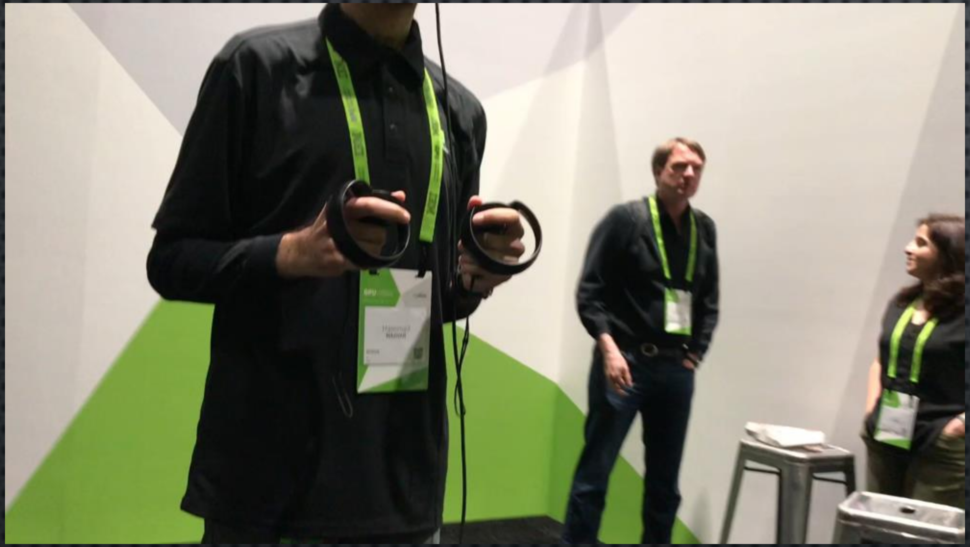

[NVIDIA ISAAC demo@GTC]

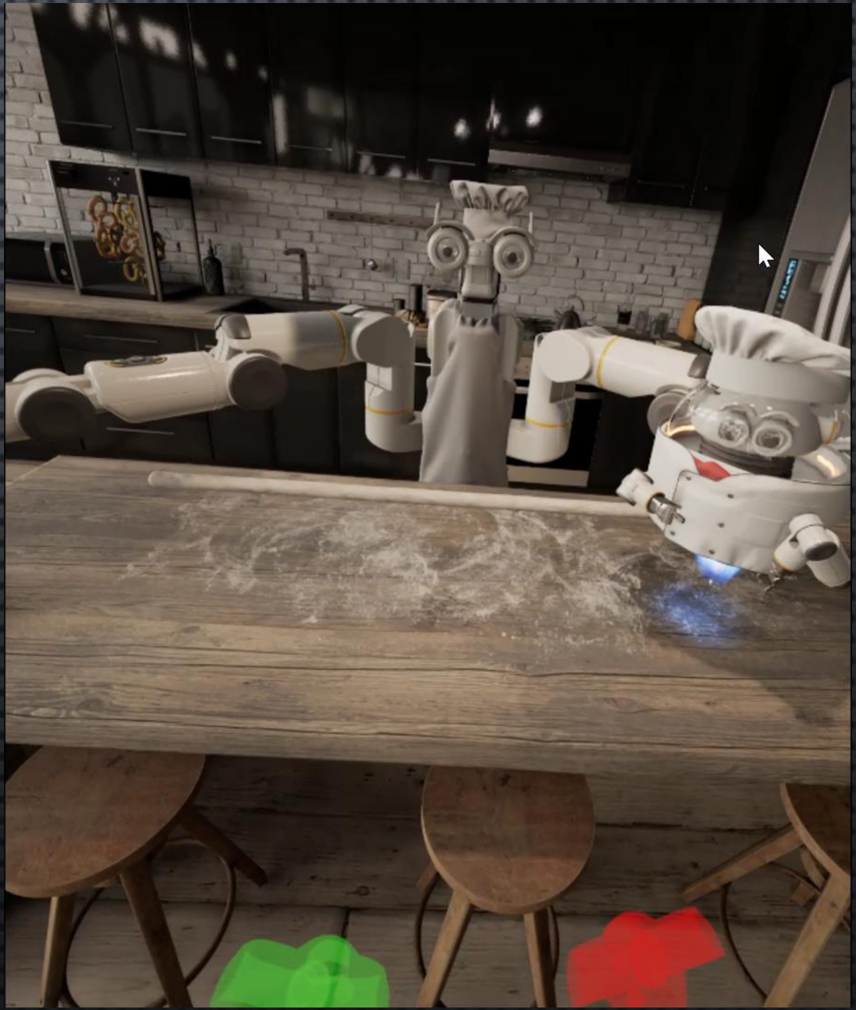

Outline

- Simulation for Development and Testing
- Simulation for Planning and Control
- Simulation for training and learning
- Kitchen as driving scenario for research
- Conclusion

WHY DO WE NEED A SCENARIO AT ALL?

- CURRENT ROBOTICS RESEARCH (ACADEMIC, INDUSTRIAL) FOCUSES ON SMALL, MOSTLY INDEPENDENT PROJECTS
- ISOLATED RESEARCH AREAS: PERCEPTION PEOPLE DO PERCEPTION RESEARCH, CONTROL PEOPLE DO CONTROL, LEARNING PEOPLE DO LEARNING
- VERY LITTLE PROGRESS IN INTEGRATED ROBOTICS ABILITIES
  - WE DON'T REALLY KNOW WHAT THE HARD PROBLEMS ARE
  - WE CAN'T BUILD ON ESTABLISHED SYSTEMS
- THE TIME IS RIPE: A JOINT PERCEPTION/CONTROL/LEARNING TEAM CAN SIGNIFICANTLY RAISE THE BAR OF WHAT'S POSSIBLE
- ONCE A BASELINE SYSTEM IS WORKING, WE CAN ENABLE THE COMMUNITY TO DO RESEARCH THAT'S CURRENTLY NOT POSSIBLE

WHY KITCHEN?

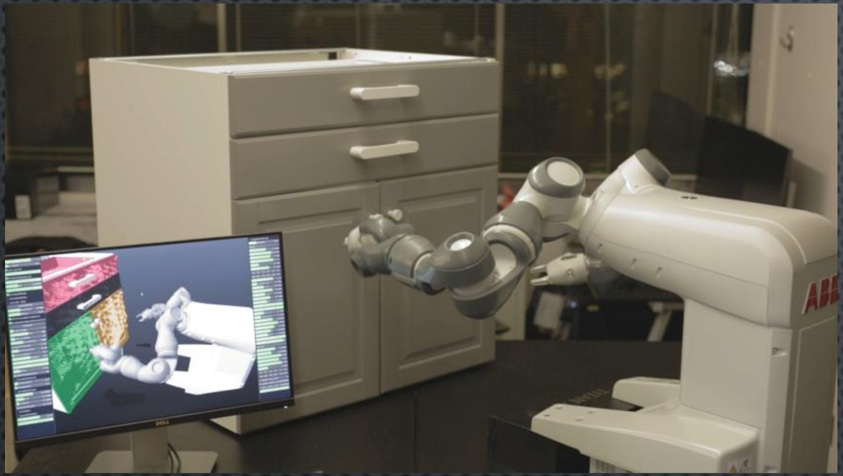

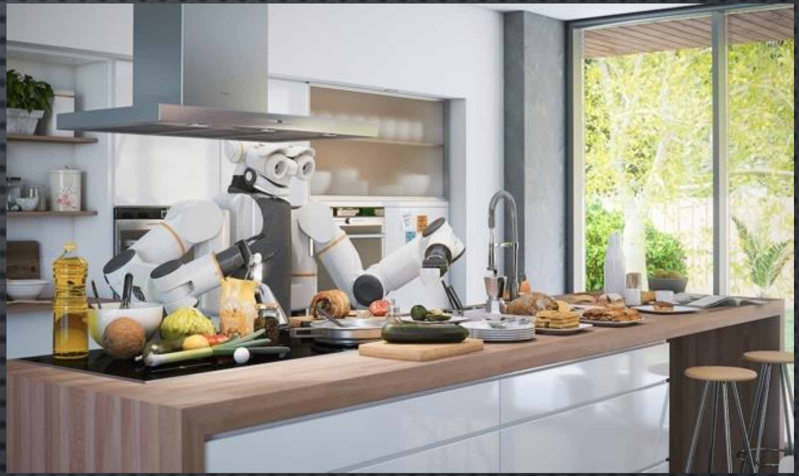

- Represents
  - Wide range of application domains: manufacturing, home, hospitals, nursing, fulfillment, laboratories
  - Most interactive manipulation research problems: grasping, control, planning, HRI, recognition, rigid objects to stuff, activity recognition, structured tasks
  - Interesting challenges for simulation, learning, representation, …
- Lends itself to progression of increasingly hard problem settings

WE'RE NOT THE FIRST ONES

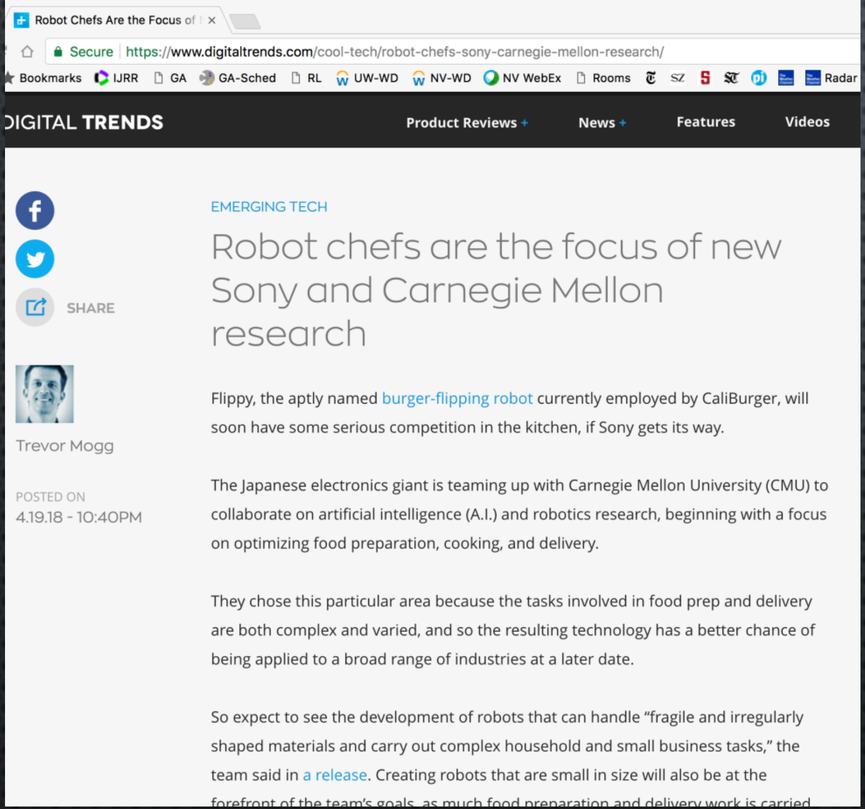

Robot Chefs Are the Focus of

Secure https://www.digitaltrends.com/cool-tech/robot-chefs-sony-carnegie-mellon-research/

Bookmarks IJRR GA GA-Sched RL UW-WD NV-WD NV WebEx Rooms Radar

DIGITAL TRENDS Product Reviews + News + Features Videos

SHARE

Trevor Mogg

POSTED ON 4.19.18 - 10:40PM

EMERGING TECH

Robot chefs are the focus of new Sony and Carnegie Mellon research

Flippy, the aptly named burger-flipping robot currently employed by CaliBurger, will soon have some serious competition in the kitchen, if Sony gets its way.

The Japanese electronics giant is teaming up with Carnegie Mellon University (CMU) to collaborate on artificial intelligence (A.I.) and robotics research, beginning with a focus on optimizing food preparation, cooking, and delivery.

They chose this particular area because the tasks involved in food prep and delivery are both complex and varied, and so the resulting technology has a better chance of being applied to a broad range of industries at a later date.

So expect to see the development of robots that can handle "fragile and irregularly shaped materials and carry out complex household and small business tasks," the team said in a release. Creating robots that are small in size will also be at the forefront of the team's goals, as much food preparation and delivery work is carried

CMU / Sony

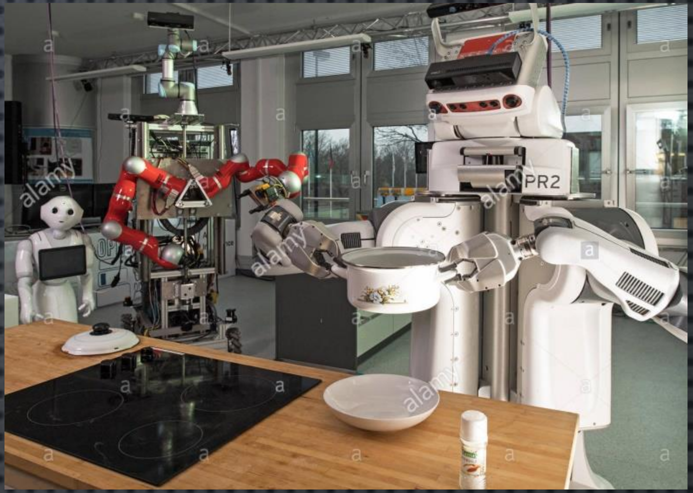

Beetz: Bremen, Germany

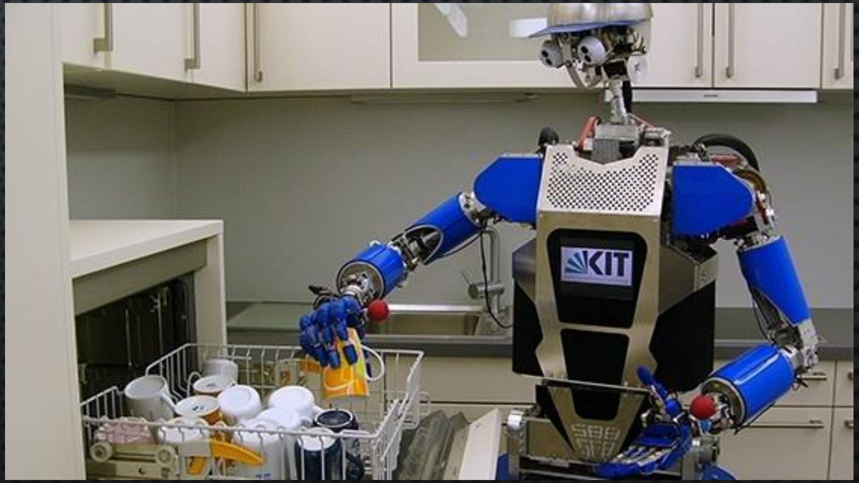

Asfour: KIT, Germany

KITCHEN: BASELINE SCENARIO [END OF 2018]

- ASSUMPTIONS
  - MODELS: 3D ARTICULATED, PHOTO-REALISTIC MODELS OF KITCHEN, ROBOT, OBJECTS
  - SENSING: RGB-(D) CAMERA AND 2D LASER ON MOBILE BASE, FORCE FEEDBACK ON MANIPULATORS, CAMERA IN ONE MANIPULATOR
- TASK: RE-ARRANGE OBJECTS INTO TARGET CONFIGURATION
  - INITIAL CONFIGURATION MIGHT BE KNOWN EXACTLY/ROUGHLY/NOT AT ALL
  - OBJECTS MIGHT BE INDIVIDUALIZED OR EVERYDAY CONFIGURED
  - TARGET CONFIGURATION COULD BE SPECIFIED VIA POINT AND CLICK INTERFACE OPEN TO PUBLIC
- KEY ABILITIES: 6D POSE DETECTION, TRACKING, TASK-MOTION-PLANNING
- RUN LONG TERM TESTS AND PROVIDE BENCHMARKS, LEARN WHAT'S HARD AND WHAT'S EASY

KITCHEN: MAKING IT HARDER

- OBJECTS
  - ADD UNKNOWN OBJECTS, REMOVE KNOWN KITCHEN ASSUMPTION
  - ADD CONTAINERS, LIQUIDS, COOKING INGREDIENTS
- PEOPLE
  - TRACK PHYSICAL STATE AND ACTIVITIES, UNDERSTAND LANGUAGE AND GESTURES
  - INTERACT, ANTICIPATE, JOINT EXECUTION
- TASKS
  - FROM PICK AND PLACE TO KITCHEN HELPER TO JOINT COOKING
  - CONTINUOUSLY IMPROVE PERFORMANCE OF KNOWN TASKS
  - LEARN NEW TASKS FROM DEMONSTRATION AND EXPERIENCE

Outline

- Simulation for Development and Testing
- Simulation for Planning and Control
- Simulation for training and learning
- Kitchen as driving scenario for research
- Conclusion

SIMULATION IN ROBOTICS

- SIMULATION FOR …
  - DEVELOPMENT AND TESTING
  - PLANNING AND CONTROL
  - TRAINING AND LEARNING
- … ALL HAVE POTENTIAL FOR HUGE IMPACT ON RESEARCH AND INDUSTRY

- SCENARIOS: NAVIGATION → MANIPULATION → PEOPLE
- STILL MANY OPEN RESEARCH PROBLEMS (TOUCH, CONTACT, CLOSE-LOOP, REPRESENTATIONS, RANDOMIZATION, SIM-TO-REAL, …)

SIMULATION IN ROBOTICS

NVIDIA IS IDEALLY SUITED TO OWN THIS AREA

EXISTING TOOLS AND WORLD EXPERTS IN PHYSICS-BASED SIMULATION, PHOTO-REALISTIC RENDERING, CONTENT CREATION, DEEP LEARNING, GPU-PROCESSING, ROBOTICS …

Robotics Research and Isaac RDK

- Leverage Isaac RDK whenever possible
- Research components become GEMs in Isaac RDK
- System helps demo benefits of GPU-based perception, control, and learning
- Enable: Raise the level of abilities across academic and industrial community
- Empower: lower the bar for commercial entry into robotic manipulation domains

ROBOTICS LAB SEATTLE TEAM

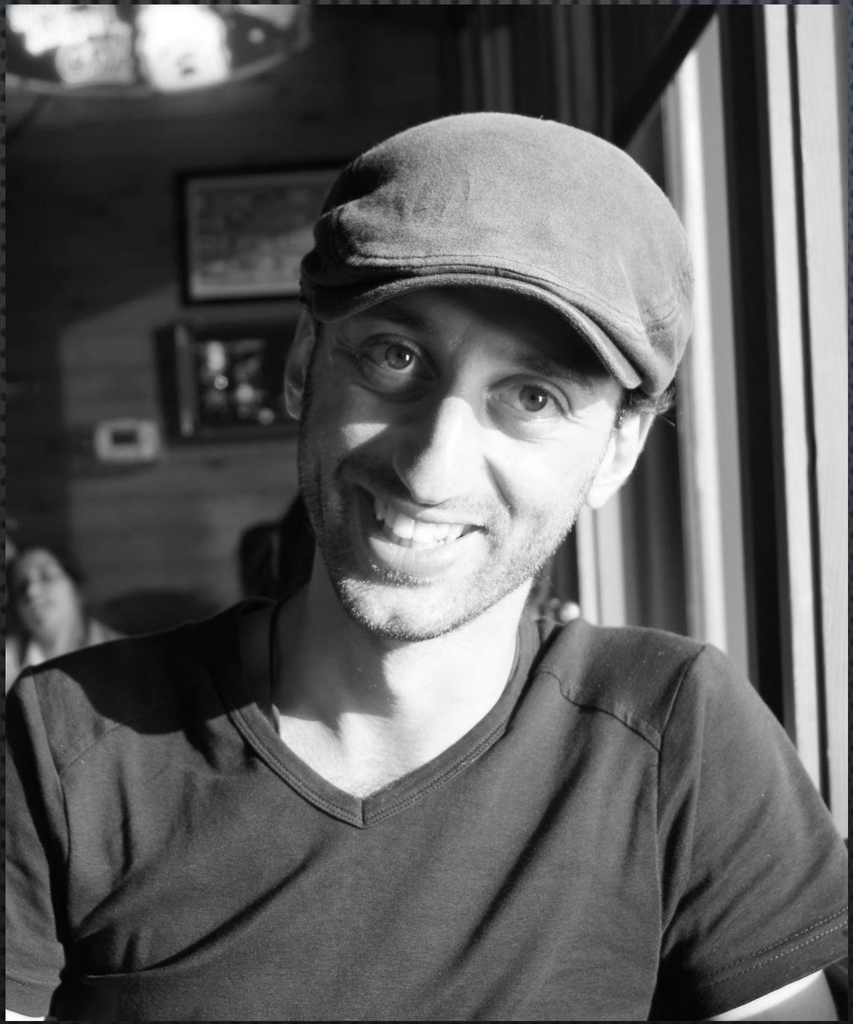

Jan Issac

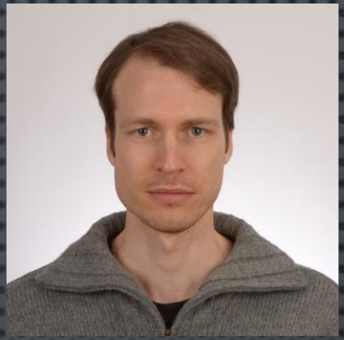

Clemens Eppner

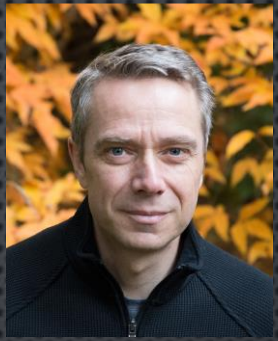

Dieter Fox

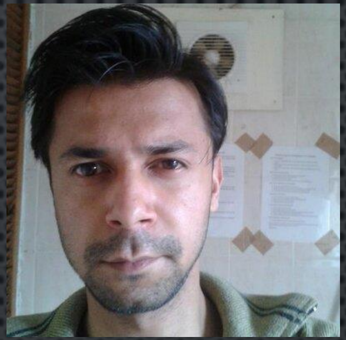

Ankur Handa

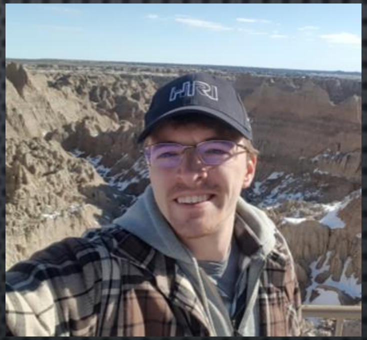

Chris Paxton

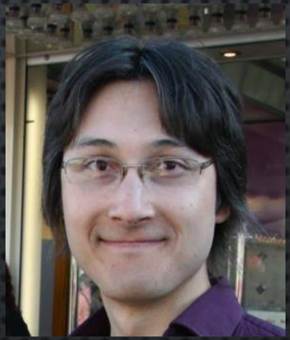

Nathan Ratliff

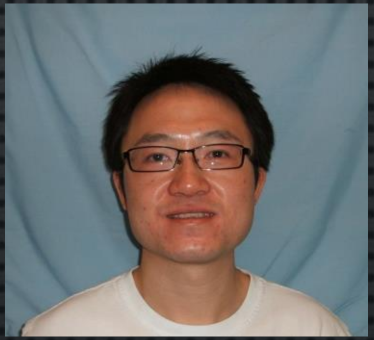

Yu Xiang

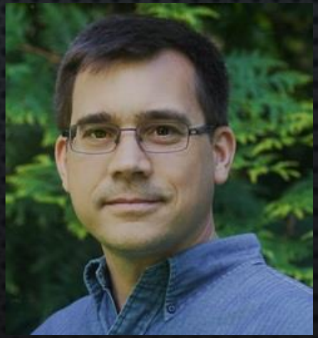

Stan Birchfield

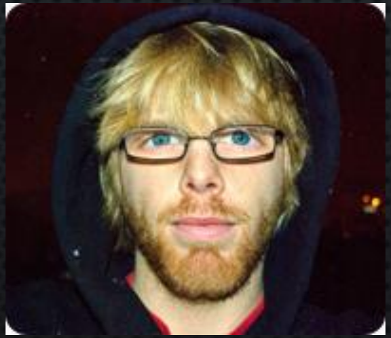

Jonathan Tremblay

Breakout Groups

- Robotics Seattle
- Robotics Pittsburgh
- Isaac SDK
- Isaac Sim
- Learning and Perception Research
- Redtail
- Robotics Industrial Connections

Modeling and Simulation in Robotics Workshop

Breakout Summary Slides

Team 1

Breakout 1

Slide 1: Consensus Thinking

- Simulation to reason about safety (constrained to important situations) in robotics systems
- To discover new behaviors to do tasks never known to be possible
- For democratization of design of robotic systems
- To provide proper approximations and guidance for developing abstractions
- For supporting co-design of robotic systems
- To generate training data for machine learning
- To benchmark and validate learning – and testing generalization of learned models
- For cheap(er) data generation

Slide 2: "Somewhat contentious" Ideas

- Enter your handful of thoughts here

Slide 3: Odds and ends, out there thoughts, fun stuff

- Enter your handful of thoughts here

Cheat Sheet Slide

- Breakout Themes, “M&S in Robotics” workshop:
  - Breakout 1: Panoramic view of opportunities
    [a time to dream]
  - Breakout 2: What’s stopping us from getting there
    [the reality check]
  - Breakout 3: Pragmatic suggestions for moving forward
    [what funding organizations, the robotics community, or other vested parties can/should do]

- <u>Breakout session</u>, things to keep in mind
  - You have 25 mins to generate your three slides
  - Select a scribe to generate your three slides
  - Decide who will present your slides in plenary
  - Do not argue within team for more than 2 mins about an idea. Move it to “<u>Slide 2</u>” and proceed
  - Generate diverse/original/out-there ideas
- <u>Plenary session</u>, things to keep in mind
  - Each team has 5 mins to present its slides
  - We seek to collect as many original ideas/points of view/opinions as possible
    - Settling contentious issues not a priority
  - Use open-floor discussion to add to what the teams have presented
  - Limit your remarks to one to two minutes. Give others an opportunity to speak. Keep it fun, keep it friendly

Modeling and Simulation in Robotics Workshop

Breakout Summary Slides

Team 2

Breakout 1

Slide 1: Consensus Thinking

- Develop in pure simulation, never touch real world
  - Can drop into real world and we know that it works
  - Rapid prototyping, tie a robotic simulator into e.g. a SolidWorks pipeline
  - Simulator validation is important aspect
  - Risk assessment, establish safety protocols within simulator
- Dream for human simulation
  - Realistic people in the simulator, important when studying human/robot interaction
    - Not just crowd simulator, but people actually doing tasks, including manipulation and cognitive models of people
    - Learn how to co-operate with people on tasks, especially on physically co-operative tasks
- Post-deployment integration
  - Capture emergent behavior of systems, tune emergent behaviors
  - Simulation play role for standardization, ex. Autonomous cars from many vendors
  - Certified libraries (from, e.g. government lab or industry standards body)
- Procedural content creation and randomization
  - Some work in the city planning community
- Human-in-the-loop simulation
  - Demonstrating how to perform things
  - Acting as adversary trying to break
  - Create and get reactions to rare/critical events
  - Mechanical turk for robot training – difficulties with hardware and internet speeds
- But the “virtuality gap” is real
  - You may not elicit the same responses from people when they know it’s not real

Slide 2: “Somewhat contentious” Ideas

- Need a Grand Unified Simulator
  - Interactions between many seemingly disjoint facets of the problem (perception, humans, deformable objects)
  - Capture uncertainty and, importantly, uncertainty of interactions between facets of the problem (e.g. multi-scale modeling)
  - Standardized environment/scenario set that are “sufficient” for covering the space of real world problems
  - 1000x real-time, enables many important applications (e.g. incorporate as part of control loop)
  - Simulator that you can dial back the complexity
  - Publically available simulators with deformable and soft surfaces
- Don’t need a Grand Unified Simulator
  - Impractical
    - Too many technologies into one place
    - Scalability
    - Radically different domains (combustion in engine, traffic in city)
  - Unnecessary
    - Actually just need mechanisms to transfer knowledge between simulators
    - You need to balance between tasks that need high fidelity and tasks that don’t
      - Controls: you just need to capture rough impact of decisions,
      - Perception: you need photo-realistic simulation?
- Design of experiment
  - Shorten the design loop for experiments, esp. in human experiments
  - Take derivatives of simulator (for controls point of view)
  - Understand structures of the problem

Slide 3: Odds and ends, out there thoughts, fun stuff

- Digital twin – important concept in industrial/manufacturing applications
- Authoring tools – game community is really good for this
- Risk assessment

Cheat Sheet Slide

- Breakout Themes, “M&S in Robotics” workshop:
  - Breakout 1: Panoramic view of opportunities
    [a time to dream]
  - Breakout 2: What’s stopping us from getting there
    [the reality check]
  - Breakout 3: Pragmatic suggestions for moving forward
    [what funding organizations, the robotics community, or other vested parties can/should do]

- Breakout session, things to keep in mind
  - You have 25 mins to generate your three slides
  - Select a scribe to generate your three slides
  - Decide who will present your slides in plenary
  - Do not argue within team for more than 2 mins about an idea. Move it to “Slide 2” and proceed
  - Generate diverse/original/out-there ideas

- Plenary session, things to keep in mind
  - Each team has 5 mins to present its slides
  - We seek to collect as many original ideas/points of view/opinions as possible
    - Settling contentious issues not a priority
  - Use open-floor discussion to add to what the teams have presented
  - Limit your remarks to one to two minutes. Give others an opportunity to speak. Keep it fun, keep it friendly

Modeling and Simulation in Robotics Workshop

Breakout Summary Slides

Team 3

Breakout 1

Slide 1: Consensus Thinking

- Robots should be modelable: we have all the parts!
- Merge uses of simulation: Classical + Data generator + Model based control.
- Hierarchical, multi-resolution simulation.
- Beyond rigid bodies: multi-physics (granular, fluid, turbulent, deformable).
- Sim-to-real transfer.
- Rich, unstructured worlds.
- Parallelization of development through compartmentalization.

Cheat Sheet Slide

- Breakout Themes, “M&S in Robotics” workshop:
  - Breakout 1: Panoramic view of opportunities
    [a time to dream]
  - Breakout 2: What’s stopping us from getting there
    [the reality check]
  - Breakout 3: Pragmatic suggestions for moving forward
    [what funding organizations, the robotics community, or other vested parties can/should do]

- Breakout session, things to keep in mind
  - You have 25 mins to generate your three slides
  - Select a scribe to generate your three slides
  - Decide who will present your slides in plenary
  - Do not argue within team for more than 2 mins about an idea. Move it to “Slide 2” and proceed
  - Generate diverse/original/out-there ideas

- Plenary session, things to keep in mind
  - Each team has 5 mins to present its slides
  - We seek to collect as many original ideas/points of view/opinions as possible
    - Settling contentious issues not a priority
  - Use open-floor discussion to add to what the teams have presented
  - Limit your remarks to one to two minutes. Give others an opportunity to speak. Keep it fun, keep it friendly

Modeling and Simulation in Robotics Workshop

Breakout Summary Slides

Team 4

Breakout 4

Slide 1: Consensus Thinking

- Simulation to reason about safety (constrained to important situations) in robotics systems
- To discover new behaviors to do tasks never known to be possible
- For democratization of design of robotic systems
- To provide proper approximations and guidance for developing abstractions
- For supporting co-design of robotic systems
- To generate training data for machine learning
- To benchmark and validate learning – and testing generalization of learned models
- For cheap(er) data generation

Slide 2: “Somewhat contentious” Ideas

- Enter your handful of thoughts here

Slide 3: Odds and ends, out there thoughts, fun stuff

- Enter your handful of thoughts here

Cheat Sheet Slide

- Breakout Themes, “M&S in Robotics” workshop:
  - Breakout 1: Panoramic view of opportunities
    [a time to dream]
  - Breakout 2: What’s stopping us from getting there
    [the reality check]
  - Breakout 3: Pragmatic suggestions for moving forward
    [what funding organizations, the robotics community, or other vested parties can/should do]

- <u>Breakout session</u>, things to keep in mind
  - You have 25 mins to generate your three slides
  - Select a scribe to generate your three slides
  - Decide who will present your slides in plenary
  - Do not argue within team for more than 2 mins about an idea. Move it to “<u>Slide 2</u>” and proceed
  - Generate diverse/original/out-there ideas
- <u>Plenary session</u>, things to keep in mind
  - Each team has 5 mins to present its slides
  - We seek to collect as many original ideas/points of view/opinions as possible
    - Settling contentious issues not a priority
  - Use open-floor discussion to add to what the teams have presented
  - Limit your remarks to one to two minutes. Give others an opportunity to speak. Keep it fun, keep it friendly

Modeling and Simulation in Robotics Workshop

Breakout Summary Slides

Team 5

Breakout 1

Slide 1: Consensus Thinking

- Role of modeling and simulation in development and testing (may be different)
  - Verification and Validation
  - Risk analysis
  - Autonomy Development vs Control Development
  - Embedded models: models used for control
  - ML for lifecycle prediction, reliability / maintainability
  - Non-temporal models, finite element models, structures analysis, load balancing
- Planning
  - gait, mobility, manipulation, multi-agent,
- HRI
  - HCI, HMI, VR, AR
- UAVs, UGV, UxV… Medical, Legged, Winged, Rotor, Manufacturing/Industrial, Home, Entertainment, Perception/Action System

Slide 2: "Somewhat contentious" Ideas

- Augment analytical models with empirical models – extent
- Maintain virtual model of robot (digital twin)
- Require metrics for safety (how do I do this?)
- XAI – interpreting/analyzing "black box" models, guarantees

Slide 3: Odds and ends, out there thoughts, fun stuff

- What is the framework for understanding when you need high/low fidelity for models
- Is a single simulator reasonable? How do you integrate multiple simulators/models?
- Assured learning

Cheat Sheet Slide

- Breakout Themes, “M&S in Robotics” workshop:
  - Breakout 1: Panoramic view of opportunities
    [a time to dream]
  - Breakout 2: What’s stopping us from getting there
    [the reality check]
  - Breakout 3: Pragmatic suggestions for moving forward
    [what funding organizations, the robotics community, or other vested parties can/should do]

- <u>Breakout session</u>, things to keep in mind
  - You have 25 mins to generate your three slides
  - Select a scribe to generate your three slides
  - Decide who will present your slides in plenary
  - Do not argue within team for more than 2 mins about an idea. Move it to “<u>Slide 2</u>” and proceed
  - Generate diverse/original/out-there ideas

- <u>Plenary session</u>, things to keep in mind
  - Each team has 5 mins to present its slides
  - We seek to collect as many original ideas/points of view/opinions as possible
    - Settling contentious issues not a priority
  - Use open-floor discussion to add to what the teams have presented
  - Limit your remarks to one to two minutes. Give others an opportunity to speak. Keep it fun, keep it friendly

Modeling and Simulation in Robotics Workshop

Breakout Summary Slides

Team 6

Breakout 1

Slide 1: time to dream

- Slide 1:
  - Agree on huge need for robot simulation

Slide 2: reality check

- Skepticism: many physical layers (each with its own complexity); no understanding of the abstraction barriers to allow to invoke the right degree of accuracy at the right level. Distinguish between Mead&Conway
- 1974 vs. Mead-Neuromorphic 1984.
- Accuracy required is a function of the robustness of controller.
- Robustness can be tested for in simulation (or it can be designed for with suitable theory).
- Sometimes can design for robustness with right abstractions (e.g. spice); as the abstraction barriers go up (e.g. VLSI) get very, very strong. Distinguish between scientific modeling vs engineering style.
- Bio-spice is an example of how hard this can be
- How to determine sufficiency in models? Question under what circumstances do we need to descend to what level of complexity? Avoid molecular dynamics. Simulation should be driven by goals – more fidelity is not necessarily better (e.g. more parameters make accuracy even harder).
- Accuracy may not be that important. But we don’t really know how and what the animals are doing when they interact with the world.
- Robustness with respect to what is important.
- ML viewpoint may be helpful – design approximators having huge numbers of parameters and gather huger amounts of data. Can we use a simulator to save the effort of collecting data? E.g. for use in MPC
- Quote: “simulations are doomed to succeed” (Louis Whitcomb, 1985) - discuss
- There are existence proofs that machines can learn on the fly (e.g. animals).

Slide 3: concrete steps forward

- A system that observes a system in the real world, build a simulation that's accurate enough for forward simulation, and to do it in real time.
- How to learn from failures of simulations;
- How to real-time choose the degree of physical fidelity + appropriate abstraction barrier.
- Analogy to drug development – how did we get the different animal models of different diseases.?
- A really accurate physical model is never going to be the way ahead:
- Take inspiration from animal brains accumulating experience over millions of years and then converges quickly in experience.
- Proper id for a change!

Issues that continue to hinder Modeling & Simulation in Robotics

(the reality check)

M&S in Robotics

- Modeling and Simulations provide a way to:
  - Prototype/design algorithms cheaply & safely
  - Generate large scale data for learning
  - Benchmark, compare and test algorithms across multiple platforms

- We expect our simulators to be:
  - Close to reality – high fidelity, photo-realistic etc.
  - General purpose – work well across environments, robots, tasks etc.
  - Easy to use – work with low effort, limited domain knowledge etc.

Issue: Model mismatch

- Non-smooth dynamics, Hybrid/Switching dynamics (friction/contact/collisions)
- Soft/Non-rigid robots/objects
- Liquids, Fluids
- Terra-mechanics, Granular media
- Sensors/Actuators
- Communication/Networking
- Human interaction, Human-Robot Interaction

Issue: Generality

- Few simulators work “out of the box” across different domains:
  - Choice of multiple models – requires expensive system ID
  - Customized representations for environment, robot and task specs

- Existing simulators try to accurately model the real-world deterministically:
  - Hard problem due to partial observability, model error etc.
  - Can work with stochastic predictions of multiple possible futures

Issue: Ease of use

- Many existing simulators are opaque and hard to use:
    - Significant learning curve for end-users; Low-level API (C/C++)
    - Hard to interface to other packages; Non-interactive (unlike game engines)

- Lack of composability, abstractions and hierarchies:
    - Abstractions delimit “levels” of appropriate modeling detail & accuracy
    - Abstractions govern model/controller/behavior composition

General issues

- Lack of (physical/simulated) **benchmarks** & shared repositories
- Lack of a "port" to **combine real-world data and human-in-the-loop** systems with analytical models and simulators
- **Poor scaling/non-determinism** of computation time
- Lack of approaches to **simulate and verify failure/edge** cases

Policy/Admin issues

- Lack of **strong collaborations** between simulation/modeling researchers and roboticists
- Lack of **established standards**: for describing robots, simulator capabilities, control/sensory interfaces etc.
- Lack of **focus on M&S** in the research community and funding agencies
  - Lack of incentive in industry/academia to work on M&S
- Lack of an established curriculum to train personnel

Modeling and Simulation in Robotics Workshop

Breakout Summary Slides

NameOfScribeHere

Team 1

Breakout 2

Group 1: Slide 1: Consensus Thinking

- Lack of funding for simulation
  - Serious software, can't be developed by students and post-docs
  - Requires staff software engineers
- How do I know the simulator is any good? (Validation)
  - How do we measure what a simulator is good at?
  - How to distinguish between a bad system ID and a bad simulator?
  - Questions to ask of a simulator?
    - Does it have to match a particular real robot (or is it just a generic test platform?)
    - Is it exploitable? Will a learning method be able to exploit a flaw in a simulator?
    - Accurate or stochastic breadth?
- Not clear whether simulated results will transfer to real world.
  - Does simulator 'accuracy' make it more likely an algorithm will transfer?
- Have ability to capture a lot more data. Not yet doing a good enough job to move data into simulation.
  - How do we build a simulator that uses both parametric and data driven modeling.
  - Not sure how to collect the *right* data for a particular robot/task.
- How to deal with vendor software?
- Noise (variance) is hard to estimate. How do we learn the correct noise model for a simulation?

Slide 2: “Somewhat contentious” Ideas

- Enter your handful of thoughts here

Slide 3: Odds and ends, out there thoughts, fun stuff

- Enter your handful of thoughts here

Cheat Sheet Slide

- Breakout Themes, “M&S in Robotics” workshop:
  - Breakout 1: Panoramic view of opportunities
    [a time to dream]
  - Breakout 2: What’s stopping us from getting there
    [the reality check]
  - Breakout 3: Pragmatic suggestions for moving forward
    [what funding organizations, the robotics community, or other vested parties can/should do]

- <u>Breakout session</u>, things to keep in mind
  - You have 25 mins to generate your three slides
  - Select a scribe to generate your three slides
  - Decide who will present your slides in plenary
  - Do not argue within team for more than 2 mins about an idea. Move it to “<u>Slide 2</u>” and proceed
  - Generate diverse/original/out-there ideas

- <u>Plenary session</u>, things to keep in mind
  - Each team has 5 mins to present its slides
  - We seek to collect as many original ideas/points of view/opinions as possible
    - Settling contentious issues not a priority
  - Use open-floor discussion to add to what the teams have presented
  - Limit your remarks to one to two minutes. Give others an opportunity to speak. Keep it fun, keep it friendly

Modeling and Simulation in Robotics Workshop

Breakout Summary Slides

Team 2

Breakout 2

Slide 1: Consensus Thinking

- **Fidelity**. We do not know what we mean by fidelity and how much we need it. It is task dependent.
- **Simulation of closed-loop behaviors**: Robotics benefits from good predictive models. In many cases this might be simpler to obtain for closed-loop behavior rather than open-loop behavior.
- **Standardization.** There is no consistency or standardization between simulators. There are also no specs or guidance as to when/which one to use.
- **Abstraction.** Abstraction comes with biases on what we intuitively think as optimal behavior. So in some sense we limit what is discoverable.
- **Trust.** How we build trust in simulators? Simulators do not output confidence: interval or distribution. They do not either output guidance as to when we should trust them.

Slide 2: “Somewhat contentious” Ideas

- **Benchmarks.** What constitutes for a good benchmark? Are benchmarks useful if they do not involve physical experiments? Do we benchmark robot simulators or robot systems?

- **Optimality and model accuracy.** Optimal behavior in the real world might require very good models. Sub-optimal might get away with worse models. Should we aim for optimality?

- **Accuracy.** Do we need better understanding of physics or better ways to simulate the physics? Partial physics might limit what is discoverable in a simulator.

Slide 3: Odds and ends, out there thoughts, fun stuff

Cheat Sheet Slide

- Breakout Themes, “M&S in Robotics” workshop:
  - Breakout 1: Panoramic view of opportunities
    [a time to dream]
  - Breakout 2: What’s stopping us from getting there
    [the reality check]
  - Breakout 3: Pragmatic suggestions for moving forward
    [what funding organizations, the robotics community, or other vested parties can/should do]

- <u>Breakout session</u>, things to keep in mind
  - You have 25 mins to generate your three slides
  - Select a scribe to generate your three slides
  - Decide who will present your slides in plenary
  - Do not argue within team for more than 2 mins about an idea. Move it to “<u>Slide 2</u>” and proceed
  - Generate diverse/original/out there ideas
- <u>Plenary session</u>, things to keep in mind
  - Each team has 5 mins to present its slides
  - The desire is to collect as many original ideas/points of view/opinions as possible
    - Settling contentious issues not a priority
  - Use open-floor discussion to add to what the teams have presented
  - Limit your remarks to one to two minutes. Give others an opportunity to speak. Keep it fun, keep it friendly

Modeling and Simulation in Robotics Workshop

Breakout Summary Slides

Team 3
Breakout 2

Slide 1: Consensus Thinking

- Gap between sim and robot community
- Simulation for design and simulation for control are different
- A systematic way to validate simulation
- Model generation is non-trivial, different methods, different hardware
- Reproduce the results using different simulators
- Simulate human reaction - many levels of abstraction of human modeling
- Reality gap (Do not know how to prioritize)
  - Flexibility of bodies
  - Contact modeling
  - Sensor noise and latency
  - Actuator model

<u>Slide 2</u>: “Somewhat contentious” Ideas

- Learn a model from real data works better in general
  - e.g. rally cars off road
- OK to overfit a model specific for the task (not seeking high fidelity general model)
- Why reality gap can be bridged?
  - It’s so hard.
  - No need to bridge and gap.

Slide 3: Odds and ends, out there thoughts, fun stuff

- Enter your handful of thoughts here

Cheat Sheet Slide

- Breakout Themes, “M&S in Robotics” workshop:
  - Breakout 1: Panoramic view of opportunities
    [a time to dream]
  - Breakout 2: What’s stopping us from getting there
    [the reality check]
  - Breakout 3: Pragmatic suggestions for moving forward
    [what funding organizations, the robotics community, or other vested parties can/should do]

- Breakout session, things to keep in mind
  - You have 25 mins to generate your three slides
  - Select a scribe to generate your three slides
  - Decide who will present your slides in plenary
  - Do not argue within team for more than 2 mins about an idea. Move it to “Slide 2” and proceed
  - Generate diverse/original/out-there ideas

- Plenary session, things to keep in mind
  - Each team has 5 mins to present its slides
  - We seek to collect as many original ideas/points of view/opinions as possible
    - Settling contentious issues not a priority
  - Use open-floor discussion to add to what the teams have presented
  - Limit your remarks to one to two minutes. Give others an opportunity to speak. Keep it fun, keep it friendly

Modeling and Simulation in Robotics Workshop

Breakout Summary Slides

Team 4

Breakout 2

Slide 1: Consensus Thinking

- The toolchain you use is the toolchain you know.
- Steep learning curve, so people don't bother with it, and see real systems as easier to use.
- What would you pay for a model that does what you want?
- The level of fidelity of what you *can* simulate needs improvement.

Slide 2: “Somewhat contentious” Ideas

- What is the role of standardization? “What does it mean to be a physics engine?” Pull of different stakeholders on what level of investment is necessary
- Does better education (of what simulation can do) lead to better use of simulators? Or more of a tie to using the things you already know?
- Some of the most effective standards are *de facto* standards

Slide 3: Odds and ends, out there thoughts, fun stuff

- What we believe hinders M&S in robotics
- How people use simulation - how people use simulators in general. Steep learning curve, so people don't bother with it, and see real systems as easier to use.
- Teaching/training tutorials/doc, better APIs, connectivity to other software, fits into standard workflows.
- What are the barriers? Physics, software, what? Maybe a bit of both. Some MEs stick to the solidworks workflow (w/out simulation). Experimenting in simulation for sensor placement, reachability, etc. are important.
- Modelica cmmty has great graphical tools, assemble them graphically, and get pretty good simulations out of it. Even with that wide availability, folks don't always use them. Robotics world, Prototype->Trying it out->Tweak params-> ship it.
- Personnel are valuable to design simulation from scratch for a particular robot. "The toolchain you use is the toolchain you know."
- Educ/outreach, broadening exposure. No standardized curriculum.
- Lack of good system ID might reduce use of simulation--if you're going to have to fix it anyway, why ever start with simulation? You can't trust what the sim is going to do, so how much effort do you put in to make it work, when you can just work with the real world?
- Would you pay $$ for a good model? What marketplaces support the level of accuracy/fidelity that you need?
- What is the area for customizable vs. special purpose?
- How to enable distributed simulation? Model the behavior that you want, and then have the simulator live up to that behavior. Borrow from things from real-time systems: build the system to behave like the model, rather than have the simulator model the real system--you get scalability.
- ARINC buses are deterministic (time-triggered), which gives you composability with guarantees of latency. The network delivers this behavior.
- Had a great simulator for motor control. However wanting to do learning algorithms for manipulation—do I work out myself how to do simulation with objects, how do I find a black-box that enables this? Combining simulators and plugins together is not easy.
- FMI - Functional Model Interfaces. There are families of simulation tools that support this kind of interface.
- The level of fidelity of what you can simulate needs improvement. Having this as a key level of your experience would be great.
- What does it mean to define a model? What level of standardization makes sense?

Cheat Sheet Slide

- Breakout Themes, “M&S in Robotics” workshop:
  - Breakout 1: Panoramic view of opportunities
    [a time to dream]
  - Breakout 2: What’s stopping us from getting there
    [the reality check]
  - Breakout 3: Pragmatic suggestions for moving forward
    [what funding organizations, the robotics community, or other vested parties can/should do]

- <u>Breakout session</u>, things to keep in mind
  - You have 25 mins to generate your three slides
  - Select a scribe to generate your three slides
  - Decide who will present your slides in plenary
  - Do not argue within team for more than 2 mins about an idea. Move it to “<u>Slide 2</u>” and proceed
  - Generate diverse/original/out-there ideas
- <u>Plenary session</u>, things to keep in mind
  - Each team has 5 mins to present its slides
  - We seek to collect as many original ideas/points of view/opinions as possible
    - Settling contentious issues not a priority
  - Use open-floor discussion to add to what the teams have presented
  - Limit your remarks to one to two minutes. Give others an opportunity to speak. Keep it fun, keep it friendly

Modeling and Simulation in Robotics Workshop

Breakout Summary Slides

Team 5

Breakout 2

Team 5: Consensus on Barriers

- Usability: large effort to build and utilize simulators, expert settings of parameters
- Composability: how to link things together, rules of construction, systemic effects, i.e. synthetic biological systems
- Validation: bound to fail; never can find appropriate model that satisfies constraints, what does it mean to be correct?
- Specifications: is current specification language rich enough? Goals in robotics may not be formally describable.
- Heterogeneity: Time and space scales, different paradigms (finite/continuous, deterministic/probabilistic, etc.)
- Observability: What is known about the environment?

- Understanding: knowing how it works is too much, but need to know when it gives garbage
- Representations and Assumptions: how to know when properly specified?
- Tradeoff between accuracy (specific) vs. adaptability (general);
- If you change parameters, will it still work?
- How much prior information is needed: real-world; is it commensurate with level of knowledge?

Team 5: Multiple viewpoints

- Improvement of simulators: end-to-end monolithic vs. modular architectures
- Will we ever get to the levels and layers of Electronic Design Automation (EDA) or Open System Interconnect (OSI)?
- Need to tie together models of sensors with environments?
- How to train or recruit people in these areas? Need for new curriculum?
- Video games lack physical models.
- Bias in robotics against modeling and simulation--cannot publish without hardware demonstration.
- How to make models/simulations to make them trusted in robotics community
- Need for Grand Challenge for this area?

Modeling and Simulation in Robotics Workshop

Breakout Summary Slides

Team 6

Breakout 2

Slide 1: Consensus Thinking

- Lack of modularity. Can independent simulators work together?
- Difficulty in choosing level of abstraction.
- Lack of appropriate training.
- Lack of benchmarks.

Breakout #3

Suggested Discussion Points

M&S in Robotics: concrete next steps. Possible breakthroughs. Speculations about disruptive technologies vis-à-vis the issue of M&S in Robotics.

- *Low hanging fruit.*
- “The long view” issues.
- Imminent landscape changes.
- Prioritization aspects. Spectacular-return-on-investment opportunities.
- The role of industry (video-games, VR, AR). What/How can we leverage? How to engage?

Let’s not just think about the end-goal
think about how to get there

Roadmap for Open-Source Benchmark Suites

- Open-source/shared repositories
  - benchmarks, challenge problems
  - robot worlds/tasks of varying complexities
  - modeling tools
  - algorithms, realistic datasets
- Evaluation pipelines
  - validate models/simulators against standard (stochastic) benchmarks and compare to other solutions
- Coordinated Physical Testbeds & Benchmark Tasks

Roadmap for Creating Modeling Standard

- Establish modeling format(s) that support sharing across different simulators.
- Create models at different abstraction/fidelity levels
  - prioritize creation of simple models
  - analyze and document what we lose with simpler models
  - analyze and document trade-off between speed and fidelity
- Push for easy-to-use models/simulators - plug-in style
- Interface that allows for data-driven methods

Build Community

Closer interaction and collaboration between roboticists, model/simulation tool developers and machine learning researchers

- Get more researchers excited and interested in modeling & simulation for robotics
  - tutorials/workshops at robotics conferences
  - add training/education about M&S to robotics training curriculum
- Create tutorials/how-to-guides
  - how to choose the ‘right’ simulator
  - create pro/cons for different simulators/models
- Organize contests
  - grand challenges

Funding Opportunities

- Funding for more meetings like this one
  - for example: get researchers with different simulator tools together
- Support by research industry and national funding agencies
  - open source simulator/models for all robots in the market
  - software engineering
  - maintenance/support
  - documentation
- Fund multiple competing efforts (similar to TensorFlow, PyTorch, etc.), and let people choose what fits best

Other Concrete Suggestions

- Combine data-driven methods with analytical models
  - Use real data to ‘identify model’ or ‘calibrate simulator’
  - Explore ways to couple simulation to reality on a continuous basis
- Combine simulators with game-engine technology
- Crowd source model and environment creation
- Utilize more precise simulators from other fields of mechanics / physics + cloud computing

Modeling and Simulation in Robotics Workshop

Breakout Summary Slides

Team 1

Breakout 3

Slide 1: Consensus Thinking

- Identify areas where M&S will have the largest impact in robotics – can we incentivize work on these areas?
  - Funding agencies
  - (Forced) Collaborations between M&S and Robotics researchers
  - Setup a virtuous cycle – encourage healthy competition across different simulators (maybe we need a “caffe” for simulators)
  - Grand challenges

- More education on multi-body dynamics and core areas for M&S
  - Not just machine learning (we should not forget physics)
  - Bridge across areas – educate software engineers on numerical methods and other relevant techniques for M&S and mechanical folks on software practices

- Rapid dissemination of knowledge across the field – models, tasks, etc.

- Can modeling & simulation alleviate ethical concerns on robotics?
  - Interpretability

Slide 2: “Somewhat contentious” Ideas

- “Modularity” can be brittle – architect your simulations to be scalable

- Learn lessons from the video game community – how to build a vibrant industry with limited tools

Slide 3: Odds and ends, out there thoughts, fun stuff

- Enter your handful of thoughts here

Cheat Sheet Slide

- Breakout Themes, “M&S in Robotics” workshop:
  - Breakout 1: Panoramic view of opportunities
    [a time to dream]
  - Breakout 2: What’s stopping us from getting there
    [the reality check]
  - Breakout 3: Pragmatic suggestions for moving forward
    [what funding organizations, the robotics community, or other vested parties can/should do]

- <u>Breakout session</u>, things to keep in mind
  - You have 25 mins to generate your three slides
  - Select a scribe to generate your three slides
  - Decide who will present your slides in plenary
  - Do not argue within team for more than 2 mins about an idea. Move it to “<u>Slide 2</u>” and proceed
  - Generate diverse/original/out-there ideas

- <u>Plenary session</u>, things to keep in mind
  - Each team has 5 mins to present its slides
  - We seek to collect as many original ideas/points of view/opinions as possible
    - Settling contentious issues not a priority
  - Use open-floor discussion to add to what the teams have presented
  - Limit your remarks to one to two minutes. Give others an opportunity to speak. Keep it fun, keep it friendly

Modeling and Simulation in Robotics Workshop

Breakout Summary Slides

Team 2

Breakout 3

- How do you establish meaningful benchmarks, fidelity?
  - Manipulation settings
  - Physical and simulation testbeds

- Fitting simulators to match benchmark problems
  - Rigid body
  - Need of modeling of touch sensors etc.

- High school competitions
  - Benchmarking Combination of tasks
  - DARPA challenge from point A to B (not switching lanes)

- Community engagement
  - Simulation grand summit (W/ standard test cases)
  - Ask for top 5-10 cool environments (tasks and settings)
  - Who would do this funding ? DoD ? NIST? Others?

- Create standardized system to include simulator as a component
  - e.g manipulation planning (centralized testbed)
  - How good is controller vs simulator (task too simple vs complex) (open loop validation)
  - Qualitative benchmark

- Composability (where does it break down?) E.g. compliance
  - Change in model = simulation is useless

Modeling and Simulation in Robotics Workshop

Breakout Summary Slides

Team 3

Breakout 3

Slide 1: Consensus Thinking and Issues

- Grand challenge: to go beyond RBD, demonstrate sensor simulation, multi-physics, etc.
  - How do we define a good challenge with clear target capabilities and clear metrics?
  - How do we define a challenge that captures attention?
  - Simulators need to predict real robot data
  - Can we have a challenge that only involves simulation (without control, etc.)
- Define appropriate levels of modeling/abstraction for different problems
- Investment in software over long time span (better communicate with funding agencies on the scientific challenges, opportunities for new capabilities, reach out to industry)
- Build benchmarks for simulation / develop standards
  - Data from real robots?
  - Verification/validation of numerical simulation: look at simple problems with known quantitative effects
- Reach-out to other fields with lots of knowledge in simulation (e.g. CFD)
  - Modeling for synthesis is different from typical modeling in science!
- Proposed modes of judgements to editors to decide whether simulation work is correct
- Write a paper together to get this vision/roadmap out

Slide 2: "Somewhat contentious" Ideas

- Enter your handful of thoughts here

Slide 3: Odds and ends, out there thoughts, fun stuff

- Modeling and simulation is harder than you think
- Modeling for synthesis is different from “typical modeling”

Cheat Sheet Slide

- Breakout Themes, “M&S in Robotics” workshop:
  - Breakout 1: Panoramic view of opportunities
    [a time to dream]
  - Breakout 2: What’s stopping us from getting there
    [the reality check]
  - Breakout 3: Pragmatic suggestions for moving forward
    [what funding organizations, the robotics community, or other vested parties can/should do]

- <u>Breakout session</u>, things to keep in mind
  - You have 25 mins to generate your three slides
  - Select a scribe to generate your three slides
  - Decide who will present your slides in plenary
  - Do not argue within team for more than 2 mins about an idea. Move it to “<u>Slide 2</u>” and proceed
  - Generate diverse/original/out-there ideas
- <u>Plenary session</u>, things to keep in mind
  - Each team has 5 mins to present its slides
  - We seek to collect as many original ideas/points of view/opinions as possible
    - Settling contentious issues not a priority
  - Use open-floor discussion to add to what the teams have presented
  - Limit your remarks to one to two minutes. Give others an opportunity to speak. Keep it fun, keep it friendly

Modeling and Simulation in Robotics Workshop

Breakout Summary Slides

Team 4

Breakout 3

Slide 1: Consensus Thinking

- 1) Benchmark models associated with real robots
  - Equivalent test bed on a real system, e.g., Robotarium at GA Tech
  - OK for cheap robots are useful but concerns about crashing for expensive ones.
  - Concrete step: Could use mixed reality for expensive robots – some robots virtual, others real, but in simulation all are ‘real’

- 2) Abstraction
  - Challenges from the real world for validation
    - How you set up the sand for your robot to walk on affects your experiment
    - Do we need different modes of how the sand operates, or is a qualitative model enough
    - Rare events, e.g., quicksand vs. sand
  - Modeling effects of hardware
    - Done in other domains, not in robotics in general. ROS does it to some extent.
  - Concrete step: Strive for sufficiency, not for perfection, for concrete next step
  - Concrete step: Leverage techniques used in other domains such as reduced order modeling

Slide 1: Consensus Thinking

- 3) Composability
  - Need a platform, or standard architecture
    - High fidelity is domain specific, how do we make it interoperable, composable, etc.
    - Framework should be able to connect varying-fidelity simulation
  - Demand-driven modeling and simulation
    - “distributed Gazebo example”, a head-simulator puts individual simulations together
    - “agent-based zones in a building example” first principles models plus data-driven models
  - Concrete step: make these more general

Slide 2: “Somewhat contentious” Ideas

- Standards for describing robot models
  - Pro: For the cases that we know what elements need to be in the model
    - Dynamics, contacts, sensors, actuators,
  - Con: For certain cases we do not know how to model

Slide 3: Odds and ends, out there thoughts, fun stuff

- Simulation is a stepping stone to data driven approaches

Cheat Sheet Slide

- Breakout Themes, “M&S in Robotics” workshop:
  - Breakout 1: Panoramic view of opportunities
    [a time to dream]
  - Breakout 2: What’s stopping us from getting there
    [the reality check]
  - Breakout 3: Pragmatic suggestions for moving forward
    [what funding organizations, the robotics community, or other vested parties can/should do]

- Breakout session, things to keep in mind
  - You have 25 mins to generate your three slides
  - Select a scribe to generate your three slides
  - Decide who will present your slides in plenary
  - Do not argue within team for more than 2 mins about an idea. Move it to “Slide 2” and proceed
  - Generate diverse/original/out-there ideas

- Plenary session, things to keep in mind
  - Each team has 5 mins to present its slides
  - We seek to collect as many original ideas/points of view/opinions as possible
    - Settling contentious issues not a priority
  - Use open-floor discussion to add to what the teams have presented
  - Limit your remarks to one to two minutes. Give others an opportunity to speak. Keep it fun, keep it friendly

Modeling and Simulation in Robotics Workshop

Breakout Summary Slides
Team 5
Breakout 3

Consensus

- Build the starting block
  - Create and distribute example environments, datasets, and models.
    - eg. Computer vision datasets
  - Create a repository for simulation datasets that are shareable.
  - Build into programs the requirement to contribute models/datasets
  - "How to" guides, tutorials, examples, simple simulations.
- What is the taxonomy of simulation data and models
  - What types of benchmarks are required for perception, control.
    - Crowdsource data generation. Filter datasets based on popularity.
- Maintenance is not fun but necessary

Contention

- Benchmarking
  - Even identical physics systems behave differently. What is benchmarking trying to achieve?
  - How to handle uncertainty?
- System Id is difficult and miserable.
  - There is no simple, automatic method for system id.
- Standardization
  - Can we have models that can be shared?
  - No incentive to do this.
  - Would be great if each robot came with a simulator or model. Even if it’s a simple simulator.
- Incentivize model development
  - How do you verify or validate generated models

Extra

- Combine data and simulation
  - New ways to collect data and large amounts of data.
  - Standardize meta-data.
- Physics models are not accurate enough
  - Learn offset models to account for incorrect models. Learn hidden state.

Modeling and Simulation in Robotics Workshop

Breakout Summary Slides

Team 6

Breakout 3

Slide 1: Consensus Thinking

- Single Framework >> Single simulator
  - Context-based/state-driven/fidelity configurable options may be part of the framework
- What can you learn from ensemble-based methods in simulations/parameters? If your robot is within the distribution, perhaps you have the correct model.
- Observation: All the interesting problems are where simulators break down

Slide 2: “Somewhat contentious” Ideas

- Model what you can, and have a simulator that is so fast that you can converge; let the simulator be wrong, but much faster than real-time
- Limit your tasks/behaviors until you have something that your simulator supports
  - Alternative: explore your desired behaviors and converge on simulation that supports it
- There is significant evidence of prediction happening in human brains.
  - In our brains are we really doing simulation or are we just using cached data?

Slide 3: Odds and ends, out there thoughts, fun stuff

- The purpose of simulation might be to develop a better robot.
- Choose the "right" simulator. Every single abstraction has one thing it's trying to address—without this understanding, it just won't be useful.
- Maybe interpreted that there is a methodology by which we can each choose the methodology that is right for them (not a single methodology for everyone)
- Even within a task, there may not be a right simulator. E.g. don't treat a full glass like you do an empty glass, it needs a different model for the same task. Same task, same robot, different level of abstraction.
- Why was ROS successful? Ease of composition through using common interfaces. Why can't we do the same things with a simulator?
- e.g., when I pick up a can w/ liquid, I don't always want to have the physics engine calculating fluid dynamics when the can isn't moving. It's OK to replan but the question is, do I need a different simulator at different times.
- A single modular framework in which we work, but not a universal simulator.
- What's the goal of what we're trying to do? What's important about our state? Putting something in a container (and moving that container) will change perhaps the perception of how something should behave.
- How does composition and distribution get to the multiple levels of abstraction. You have the high-fidelity that you trust. You run this simulation on different scenarios and then see what happens. You run the other models on the same scenario and then you see what happens. You see how closely they agree, and if they're close enough, then this model is sufficient for that task. But if you are now doing this in outer space, you might need to do it differently.
- You have a great solution that is really slow—that's the real world. However, there are safety issues, and there are reproducibility issues.
- There are problems with using real data to train, you need to generate synthetic data to prevent doing dangerous things.
- Phantom robots- 3dof robots that are like a big finger. They are so fast that if you try to use them at their limits, they stop being rigid because they start to bend. If you slow them down, they are rigid again. By changing the task/boundaries of the task, you change the resolution of what's required.
- I have a really hard problem, but if I can solve things fast enough I can just use linearization all over the place.
- Pragmatically speaking, how do you capture what are the limits of the system (or discover what appropriate limits of the system) are, so that they can be used the right way.
- Using MPC/adaptive control allows incorrect models to work over time: note, also true for any kind of feedback control, key is to get the updates.
- There is significant evidence of prediction happening in human brains. In our brains are we really doing simulation, or are we just using cached data?
- What if instead of 1 copy of a simulation, you have 10,000 copies of a simulation. What would you learn from this?
- Do you want a policy that is robust to many different kinds of worlds?

Cheat Sheet Slide

- Breakout Themes, “M&S in Robotics” workshop:
  - Breakout 1: Panoramic view of opportunities
    [a time to dream]
  - Breakout 2: What’s stopping us from getting there
    [the reality check]
  - Breakout 3: Pragmatic suggestions for moving forward
    [what funding organizations, the robotics community, or other vested parties can/should do]

- Breakout session, things to keep in mind
  - You have 25 mins to generate your three slides
  - Select a scribe to generate your three slides
  - Decide who will present your slides in plenary
  - Do not argue within team for more than 2 mins about an idea. Move it to “Slide 2” and proceed
  - Generate diverse/original/out-there ideas
- Plenary session, things to keep in mind
  - Each team has 5 mins to present its slides
  - We seek to collect as many original ideas/points of view/opinions as possible
    - Settling contentious issues not a priority
  - Use open-floor discussion to add to what the teams have presented
  - Limit your remarks to one to two minutes. Give others an opportunity to speak. Keep it fun, keep it friendly
